# Ribosome External Electric Field Regulates Metabolic Enzyme Activity: The RAMBO Effect

**DOI:** 10.1021/acs.jpcb.4c00628

**Published:** 2024-07-16

**Authors:** Jianchao Yu, Lisa M. Ramirez, Qishan Lin, David S. Burz, Alexander Shekhtman

**Affiliations:** † Department of Chemistry, University at Albany, State University of New York, Albany, New York 12222, United States; ‡ RNA Epitranscriptomics & Proteomics Resource, University at Albany, State University of New York, Albany, New York 12222, United States

## Abstract

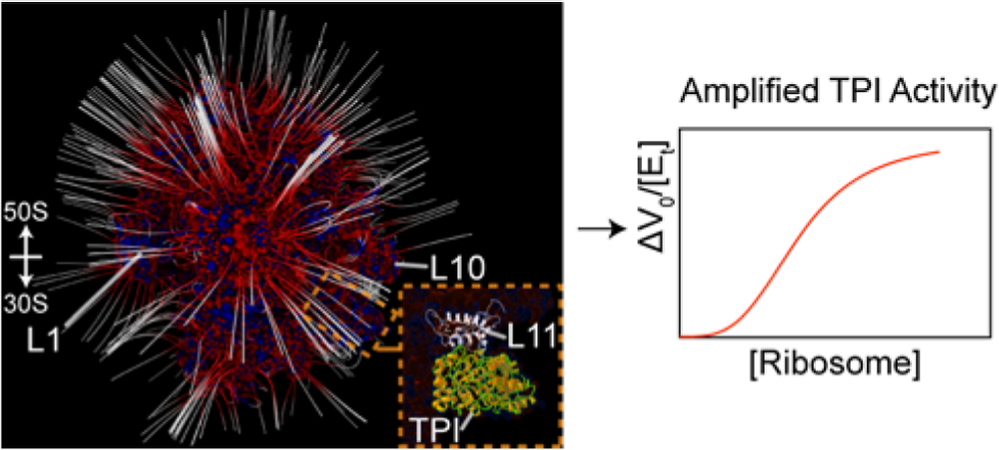

Ribosomes bind to many metabolic enzymes and change their activity. A general mechanism for ribosome-mediated amplification of metabolic enzyme activity, RAMBO, was formulated and elucidated for the glycolytic enzyme triosephosphate isomerase, TPI. The RAMBO effect results from a ribosome-dependent electric field-substrate dipole interaction energy that can increase or decrease the ground state of the reactant and product to regulate catalytic rates. NMR spectroscopy was used to determine the interaction surface of TPI binding to ribosomes and to measure the corresponding kinetic rates in the absence and presence of intact ribosome particles. Chemical cross-linking and mass spectrometry revealed potential ribosomal protein binding partners of TPI. Structural results and related changes in TPI energetics and activity show that the interaction between TPI and ribosomal protein L11 mediate the RAMBO effect.

## Introduction

Electrostatic interactions are ubiquitous in biological systems due to the omnipresence of charged biomolecules in polar mediums^1,2^ and are essential for molecular recognition, macromolecular stability, and catalysis.^1,3^ In biocatalysis, nature often adopts a strategy that ascribes catalytic activity to electrostatic stabilization of the transition state, so-called electrostatic catalysis.^4^ This effect was first proposed^5^ in the 1980s and was confirmed nearly a decade ago,^6^ revealing that enzymes harness preorganized local electric fields generated by charged residues at their active sites to catalyze reactions with bound substrates by stabilizing the alignment of the transition state dipole with an external electric field, EEF, applied at the active site. Oriented EEFs, OEEFs, have garnered considerable attention recently in synthetic chemistry as smart effectors^7^ to control the reactivity of chemical reactions based on the orientation of the EEF with respect to the reaction center.^7−10^ The reaction center is the axis^7,9,11^ that denotes the direction of electron reorganization from reactants to products. Aligning the EEF along this axis enhances catalysis, while reversing the orientation inhibits catalysis.

**Figure 1:**
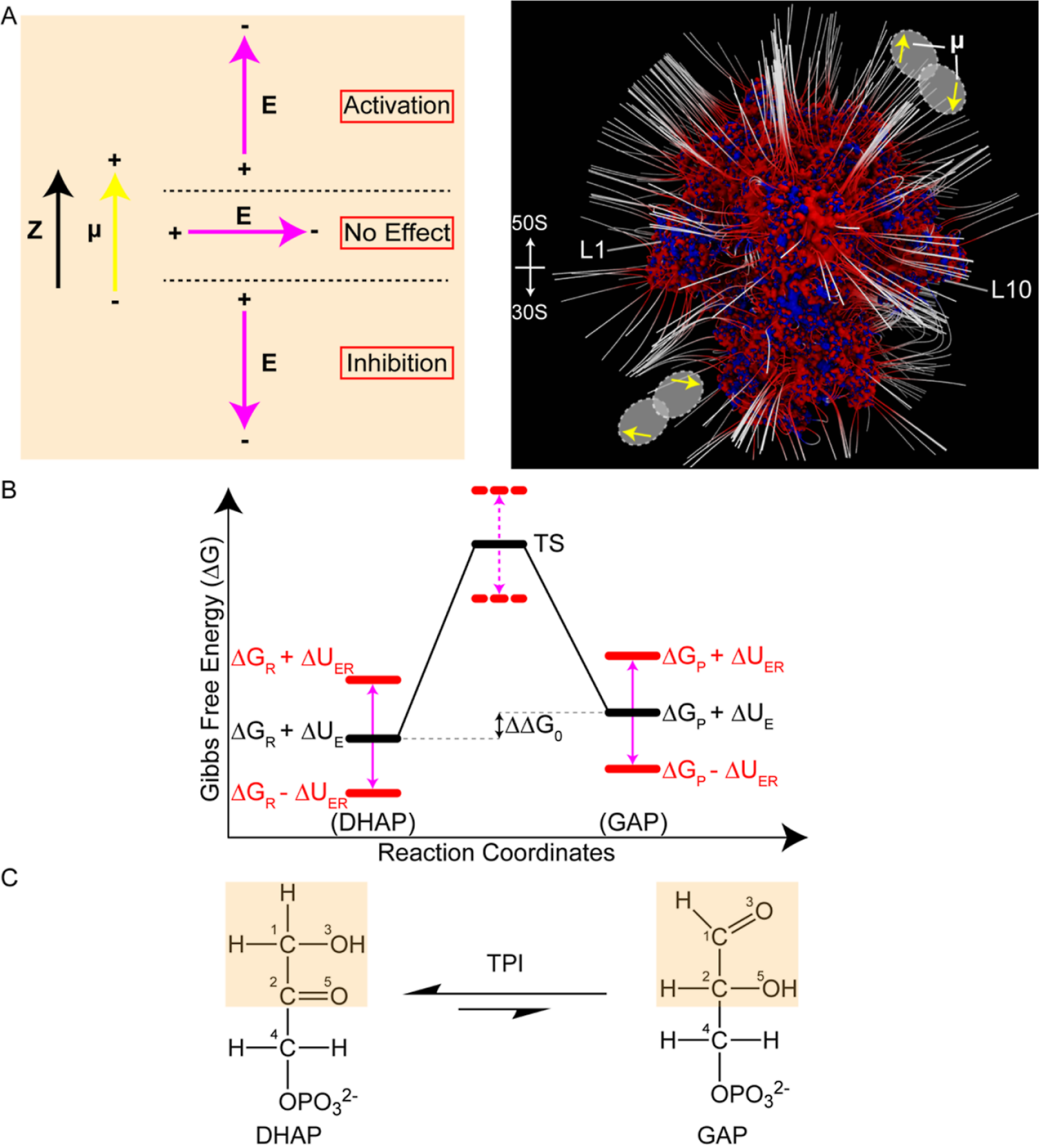
Highly negative electrostatic potential of intact ribosomes gives rise to the RAMBO effect. (A) (Left) Interaction energy generated when substrate molecular dipole interacts with ribosome electric field. The substrate molecular dipole, **μ**, oriented along the reaction axis, *Z*, can align or misalign with the ribosome electrical field, ***E***, to enhance or inhibit catalytic activity. (Right) Two randomly oriented enzyme dimers are shown bound to a ribosome (PDB entry 5UYK) with the active site substrate dipole moments, **μ**, indicated by yellow arrows. Blue and red regions correspond to positive >5 *k*_B_*T/e* and negative <−5 *k*_B_*T/e* iso-surface potentials and the electric field lines of the ribosome are in white. Note that the bound enzymes do not experience Debye screening^3^ at the ribosomal surface. L1 and L10 proteins and the 50S and 30S subunits are labeled for the purpose of orientation. (B) Free energy profile of enzyme reaction coordinates. Δ*G*_R_ and Δ*G*_P_ are the ground state Gibbs free energies of the reactant and product, Δ*U*_E_ is the interaction energy between substrate and free enzyme, and ΔΔ*G*_0_ is the difference between the ground states in the free enzyme. Enzyme binding to ribosomes results in an active site interaction energy, Δ*U*_ER_, that can increase or decrease the energy of the ground state of the reactant and product, the difference between which drives the reaction. For unimolecular reactions, the contribution from the transition state, TS, cancels out when the ratio of forward and reverse rate constants is measured, revealing only the differences in the molecular dipole-electric field interaction energies. (C) Reaction catalyzed by TPI. The image was generated using ChemDraw, with substrates labeled according to atom number following ChemDraw’s notation rules.

Intact ribosomes, which exert strong local electric fields within the cell because of negatively charged rRNAs that comprise its structure, bind to hundreds of cytosolic proteins,^12,13^ including metabolic enzymes.^13^ Chloroplast and mitochondrial ribosomes also bind to metabolic enzymes.^14,15^ Ribosomal binding alters the activities of several metabolic enzymes, providing insight into an effect of metabolic regulation called Ribosome-Amplified MetaBOlism or RAMBO.^16,17^ However, the mechanism behind this effect remains unclear. We propose that a primary determinant that gives rise to the RAMBO effect occurs when an enzyme bound to an intact ribosome through a specific interaction surface orients the substrate molecular dipole so that it can interact with the electric field of the ribosome to amplify the activity of the ribosome-bound enzyme (Figure 1A) positively or negatively. The binding results in a ribosome-dependent field-dipole interaction energy, *U*_ER_ ∼ **μE**_R_, where **μ** is the molecular dipole and **E**_R_ is the strength of the local ribosomal electric field. *U*_ER_ can be estimated to be ∼4 *k*_B_*T*, by assuming the characteristic magnitudes of **μ** and **E**_R_ to be 8 *ea*_0_ and 0.5 *k*_B_*T*/(*ea*_0_),^18^ respectively, where *k*_B_ is the Boltzmann constant, *a*_0_ is the Bohr radius, *e* is the electron charge, and *T* is temperature. *U*_ER_ can increase or decrease both the transition and the ground states of the reactant and product, the difference between which drives the reaction (Figure 1B).

Triosephosphate isomerase, TPI, a well-studied metabolic enzyme^19^ highly conserved from prokaryotes to eukaryotes,^20^ was selected as a probe to validate the RAMBO effect, due to its reliance on electrostatic effects within its active sites for catalytic function.^21−23^ Catalytically active TPI is an obligate homodimer consisting of two identical 248 residue (27 kDa) αβ barrel protein cores with active sites located in the center of the barrels. TPI catalyzes the fifth step of glycolysis, the interconversion between dihydroacetone phosphate, DHAP, and glyceraldehyde 3-phosphate, GAP (Figure 1C). Activity is regulated in part by the coordinated movement of a flexible 11 amino acid (residues 166–176) loop, loop 6, which helps with substrate binding and catalysis.^24,25^ Beyond its fundamental role in glycolysis, TPI moonlights as a biomarker of cancer, a regulator of the cell cycle, and a virulence factor for pathogens.^20,26^ This multifunctionality implies that TPI may be a promising target for cancer treatment^20^ and protozoal infection.^27^ Interestingly, potential interactions between TPI and the ribosome have been suggested by ribo-interactome analysis,^13^ although direct evidence remains elusive.

To substantiate the RAMBO effect and enhance our understanding of regulation in cellular metabolism, a phenomenological equation relating TPI kinetics to molecular dipole-electric field interaction energies was developed. This equation provides guidelines for the experiments and computational simulations performed in this work. The isomerization reaction catalyzed by TPI is unimolecular, where the reactant and product share identical transition states. The reaction kinetics can be measured independently in both directions. By comparing the ratio of rate constants for each substrate in the presence of ribosomes with the ratio in the absence of ribosomes, the energetic contribution of transition states cancels out to simplify calculations.

Nuclear magnetic resonance, NMR, spectroscopy^16^ was used to measure the kinetics of TPI in the absence and presence of intact ribosomes. Chemical cross-linking coupled with mass spectrometry,^28^ XL-MS, and molecular docking,^29^ were used to identify structurally compatible model complexes between TPI and ribosomal proteins, RPs. The molecular dipole moments of substrates bound to free and ribosome-bound enzymes were calculated using quantum chemistry software,^30^ and a model of continuum electrostatics^31^ was used to estimate the magnitude and orientation of the ribosome EEF at the active site in the absence and presence of ribosomes for each model. The results provide a basis to verify the congruence between TPI activity and the molecular dipole-electric field interaction energy and substantiate the postulate that this underlies the RAMBO effect.

## Materials and Methods

### Labeling and Purification of Triosephosphate Isomerase

TPI was purified using a published method with slight modifications.^32^ Plasmid pET-21a(+)-TPI, which confers ampicillin resistance and expresses 26.6 kDa 248 residue TPI from *G. gallus* (uniprot P00940), was purchased from Genscript and transformed into *Escherichia coli* strain BL21 (DE3) for overexpression. Ten milliliters of Luria broth, LB, medium containing 150 μg/mL of carbenicillin was inoculated with a single colony of transformed cells and incubated at 37 °C and 225 rpm overnight. The overnight culture was transferred to 1 L of LB medium containing 150 μg/mL of carbenicillin and incubated at 37 °C and 225 rpm until an OD_600_ of 0.7–0.8 was reached. Expression of TPI was induced with 1 mM IPTG, and the cells were incubated at 30 °C and 225 rpm for 18 h. For uniform ^15^N labeling, TPI was induced in 1 L of minimal medium, M9, containing 0.4% (w/v) glucose, 1.0 g/L [^15^N] ammonium chloride, and 150 μg/mL of carbenicillin. For in-cell experiments, 250 mL of the culture was pelleted and processed as published previously.^33^ The harvested cell pellet was resuspended in 20 mL of lysis buffer, 10 mM Tris-HCl, pH 7.5, containing 1 mM 4-benzenesulfonyl fluoride hydrochloride, AEBSF, as a protease inhibitor. The cell suspension was sonicated on ice for 2 min per cycle at 25% amplitude using a model 250 Digital Sonifier (Branson) with a pulse of 0.3 s on and 1.1 s off. The resulting lysate was centrifuged at 50,000*g* for 1 h at 4 °C, and the supernatant was filtered by using 0.45 μm syringe filters (Pall) before loading onto a 3 × 5 mL HiTrap DEAE-FF weak anion-exchange column (Amersham Bioscience) that had been pre-equilibrated with lysis buffer at a flow rate of 2 mL/min. Protein was eluted from the column using a linear gradient of 0–150 mM KCl in lysis buffer over 200 min. Fractions were collected using a BioLogic DuoFlow chromatography system (Bio-Rad) and checked using 12% sodium dodecyl sulfate–polyacrylamide gel electrophoresis, SDS-PAGE. Fractions containing TPI were pooled and dialyzed twice against lysis buffer. The dialyzed protein was reloaded onto a HPLC DEAE-FF column at a flow rate of 2 mL/min and eluted with a linear gradient of 0–100 mM KCl in a lysis buffer over 210 min. Fractions with over 95% purity were collected, pooled, and dialyzed into storage buffer, 25 mM Tris-HCl, pH 8.0, 0.1 M NaCl, 0.02% NaN_3_, and stored at 4 °C for later experiments.

Overexpression of plasmid pET-21a(+)-His-TPI (Genescript), which codes for C-terminal His-tagged TPI, was the same as for wild-type TPI. The harvested cell pellet was resuspended in 25 mL of His-TPI lysis buffer, 50 mM sodium phosphate, pH 8.0, 500 mM NaCl, and 20 mM imidazole containing 1 mM AEBSF and 10 mM 2-mercaptoethanol. The sonication settings were identical with those used above. The cell lysate was centrifuged at 50,000*g* for 1 h at 4 °C, and the supernatant was filtered through a 0.45 μm filter before incubation with a 5 mL bed volume of nickel-nitrilotriacetic acid, Ni-NTA, agarose resin equilibrated with His-TPI lysis buffer at room temperature, RT for 30 min. The resin suspension was poured into the batch purification column and rinsed five times with 20 mL of wash buffer, 50 mM sodium phosphate, pH 8.0, 500 mM NaCl, and 50 mM imidazole. His-TPI was eluted using 2.5 mL of elution buffer, 50 mM sodium phosphate, pH 8.0, 500 mM NaCl, and 250 mM imidazole. The elution step was repeated 12 times. Each fraction was assessed on 12% SDS-PAGE, and samples with a purity over 95% were pooled and dialyzed twice into 5 L of cross-linking buffer, 10 mM sodium phosphate, pH 7.5, 100 mM NaCl, and 10 mM MgCl_2_. The purified protein was concentrated using a 10,000 MWCO centrifugal filter device (Amicon). All TPI concentrations were determined from the absorbance at 280 nm measured on a NanoDrop 2000 instrument (Thermo Fisher). An extinction coefficient of 67,420 M^–1^ cm^–1^ was calculated using the ProtParam tool of Expasy Server.^34^

### Purification of Ribosomes

Intact 70S ribosomes were isolated from *E. coli* MRE600 at mid log phase and were purified using a previously published protocol^16^ that include a high salt, 1 M NH_4_Cl, wash, and ultracentrifugation step to remove ribosome-associated factors. The resulting ribosome pellet was resuspended in ribosome storage buffer, 10 mM potassium phosphate buffer, pH 6.5, 10 mM magnesium acetate, and 1 mM DTT, and stored at −80 °C for TPI kinetics. Ribosome purity was further assessed through protein gel analysis that showed the absence of *E. coli* trigger factor, MW ∼ 50 kDa (Figure 4A, lane 3), which indicated that the high salt wash effectively removed ribosome-associated factors. The presence of loosely associated stalk RP, L7/L12, in the ^1^H–^15^N heteronuclear single quantum coherence, HSQC, spectra of [U–^15^N]-ribosomes (Figure S1) also confirmed that the high salt wash did not compromise the integrity of the RPs. Finally, the presence of the flexible RP S1, migrating as an ∼68 kDa band in the protein gel (Figure 4A, lane 3), further supports the idea that the ribosome preparations were intact.

For 2D HSQC experiments, ribosomes were dialyzed into NMR buffer, 10 mM MES, pH 6.5, 10 mM NaCl, 0.02% NaN_3_, and 10 mM MgCl_2_. The absorbance at 260 nm was used to determine ribosome concentrations using an ε_0.1%_ of 15 mL mg^–1^ cm^–1^. Only ribosome solutions with a 260 nm:280 nm ratio of 1.96–2.05 were employed in these experiments.

### NMR Spectroscopy

Enzyme kinetics were monitored by collecting ^1^H NMR spectra at 290 K with water suppression by excitation sculpting^35^ on a 700 MHz Bruker Avance II NMR spectrometer equipped with a TXI cryoprobe. Pseudo two-dimensional, pseudo-2D, experiments were recorded with 16 transients and a 2.13 s interval between transients, 1.13 s acquisition time, and a 1 s relaxation delay. These experiments incorporated td1 = 50 time data points as the second dimension. The spectral width in the proton dimension was 20 ppm.

^1^H–^15^N HSQC, spectra of uniformly ^15^N-labeled TPI, [U–^15^N]-TPI, were recorded with Watergate water suppression.^36^ All 2D NMR spectra were collected at 298 K on a 700 MHz Bruker Avance II NMR spectrometer equipped with a TXI cryoprobe; 1024 and 128 points were acquired in the proton and nitrogen dimensions, respectively, with 208 transients. The spectral widths in the proton and nitrogen dimensions were 14 and 35 ppm, respectively.

To assess the apparent binding affinity between ribosomes and substrate-bound TPI, [U–^15^N]-TPI was dialyzed into kinetic buffer, 10 mM sodium phosphate, pH 7.5, and 0.1 M NaCl and bound to 5 mM DHAP or 1 mM GAP. ^1^H–^15^N HSQC spectra were collected as the protein was titrated with 0, 1.25, 2.5, 5, 7.5, 10, 12.5, 15, 20, 25, and 30 μM ribosomes. One and 1024 points in the nitrogen and proton dimensions, respectively, were acquired with 4096 transients at 290 K for the amide proton envelope from 6.30 to 10.54 ppm. The spectral widths in the proton and nitrogen dimensions were 14 and 35 ppm, respectively.

To record changes in the ^1^H–^15^N HSQC spectra of free and substrate-bound TPI due to quinary interactions with intact ribosomes, the final ribosome pellets were resuspended in NMR buffer and 0, 0.5, 1, and 2 μM ribosomes were titrated against 50 μM of [U–^15^N]-TPI, with or without 5 mM DHAP or 1 mM GAP, in 0.3 mL of NMR buffer containing 10% D_2_O. All spectra were processed through Topspin 3.6.2 (Bruker) and analyzed using Computer-Aided Resonance Assignment (CARA).

### TPI Activity Assay

Purified TPI was dialyzed into kinetic buffer prior to making the kinetic measurements as previously described.^16^ TPI activity was measured in both directions by using DHAP lithium salt (Sigma-Aldrich) and d-GAP (Sigma-Aldrich) as substrates. Assays were conducted in 0.5 mL of kinetic buffer containing 10% D_2_O and 7.5 mM DHAP or 1 mM GAP in the absence and presence of 1 μM ribosomes. Reactions were initiated by adding TPI to a final concentration of 1.6 nM for the DHAP saturation curve and 0.13 nM for the GAP saturation curve. The intensity of the DHAP proton peak H1 at ∼3.36 ppm was monitored to determine the rate of reactant loss or product formation. All experiments were conducted in triplicate. The NMR operation and kinetic data analysis, followed a previously published method.^16^ DHAP H1 peak volumes, exported from MestReNova 14.0.0 (Mestrelab Research), were converted to molar concentrations by normalization to a 1 mM DHAP standard. Initial velocities were calculated from the first 10 data points.

Substrate saturation curves were fit to the Michaelis–Menten equation using GraphPad Prism 9
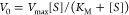
1where *V*_0_ is the initial reaction rate, *V*_max_ is the maximum velocity, [*S*] is the substrate concentration, and *K*_M_ is the Michaelis–Menten constant. The rate constant *k*_cat_ was calculated as *V*_max_/[*E*_t_], where [*E*_t_] is the total enzyme concentration. The independent sample *t*-test was used to assess the statistical significance of the kinetic parameters measured in the absence and presence of ribosomes. Cohen’s *d* measures were calculated to determine the magnitude of this effect. Cohen’s *d* measures of 0.2, 0.5, and 0.8 indicate small, medium, and large differences in the mean experimental values, respectively.^37^

### Analysis of TPI-Ribosome Binding Data

The integrated intensities of the amide proton envelope obtained from NMR titrations of TPI with ribosomes were exported from Topspin 3.6.2 (Bruker) and analyzed with GraphPad Prism 9 by using the total binding equation
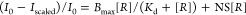
2where *I*_scaled_ is the scaled intensity of TPI in the amide proton range upon ribosome titration, *I*_0_ is the initial integrated intensity of TPI without ribosomes, *B*_max_ is the maximum specific binding, [*R*] is the ribosome concentration, *K*_d_ is the dissociation constant between substrate-bound TPI and ribosomes, and NS is the slope of nonspecific binding.

### Cross-Linking Mass Spectrometry Analysis

Chemical cross-linking experiments were conducted with 100 μM His-TPI and 10 μM ribosomes in 20 μL of cross-linking buffer, 10 mM sodium phosphate, pH 7.5, 100 mM NaCl, and 10 mM MgCl_2_, with or without 0.5, 1, and 2 mM of three homobifunctional amine-to-amine cross-linkers: bis-sulfosuccinimidyl suberate, BS_3_, space arm 11.4 Å, bis-*N*-succinimidyl-(pentaethylene glycol) ester, BS(PEG)_5_, space arm 21.7 Å, and bis-*N*-succinimidyl-(nonaethylene glycol) ester, BS(PEG)_9_, space arm 35.8 Å, respectively. Reactions were incubated at RT for 30 min and quenched by adding 50 mM Tris-HCl, pH 7.6. Cross-linking reactions were verified by capturing and eluting His-TPI from Ni-NTA agarose resins under denaturing conditions (8 M urea) following Qiagen’s standard protocol. Results were visualized by using 12% SDS-PAGE. Cross-linked TPI-ribosome bands were excised from the gels. As controls, cross-linked TPI alone, cross-linked ribosome alone, and a blank at the same molecular weight position were excised. The excised gels were minced into ∼1 mm^3^ pieces, followed by in-gel tryptic digestion as described by Xue *et al.*(38) The tryptic peptides were extracted from the gel using 50% acetonitrile plus 5% formic acid. The resulting peptide solutions were lyophilized and redissolved in 30 μL of 0.1% trifluoroacetic acid/3% acetonitrile for LC-MS/MS analysis. Cross-linking LC-MS/MS measurements were performed on a Thermo Orbitrap Velos as previously described.^16^

### Analysis of Cross-Linked Peptides

Tandem spectrum data were processed using the pLink 2 software package^39^ with modified settings: 500 Da ≤ peptide mass ≤ 6000 Da, 5 ≤ peptide length ≤ 60, precursor tolerance of ±5 ppm, fragment tolerance of ±10 ppm, and false discovery rate of ≤5%. For the cross-linker, BS(PEG)_5_, a monoisotopic linker mass shift of 302.136555 Da and a mono mass shift of 320.14712 Da were set. Cysteine carbamidomethylation and methionine oxidation were selected as the fixed and variable modifications, respectively. To maximize the outputs of potential cross-linked peptides, a parameter including either trypsin, allowing up to four missed cleavages, or nonspecific was used for tandem MS spectrum assignment. Only intermolecular cross-links with a precursor mass error within ±3 ppm were considered for the next step after searching. Fragmentation spectra were manually checked by pLabel,^40^ a software tool of pFind.^39^ The solvent accessibility of cross-linked residues from TPI and RPs was analyzed using GETAREA,^41^ with a water probe radius of 1.4 Å. Manual inspection was performed using rigid structural models in UCSF ChimeraX version 1.3.^42^ Finally pLink analysis identified solvent-accessible cross-linked peptides for each candidate (Tables S1 and S2).

### Verification of Cross-Linked Data and Molecular Docking

The PDB tool and PDB file editor in Phenix GUI v1.19^43^ software were used to prepare PDB files. TPI dimers were edited into a single chain, renumbering the second monomer residues to avoid duplication (Table S3). Individual RPs or combinations of RPs were also edited into a single chain, renumbering residues for combinations and including proximal rRNAs and RPs to minimize spatial clash. Unambiguous restraints, defined as distance restraints with an allowable C_α_-C_α_ range from 0 to 34.5 Å and an upper limit^44^ of 3 Å, were generated from intermolecular cross-links and set for HADDOCK^29^ and DisVis^45^ analysis. The analysis identified and deleted spatial violations in the cross-links initially identified for both end-to-end and side-to-side binding modes (Figure 4B) and predicted interacting residues in the TPI-RP binding interfaces that may drive the molecular docking process (Table S2).^46^ To filter out violations of these restraints, an initial coarse scan of DisVis (rotational sampling interval 15°, voxel spacing 1 Å, interaction radius 3 Å, maximum clash volume 200 Å^3^, and minimum interaction volume 300 Å^3^) was performed for each candidate RP and TPI. This process identified any nonzero accessible complexes that aligned with all restraints in a particular set. The most likely violated restraint, as indicated by its z-score value and violation percentage, was deleted, and the coarse scan was rerun. Ambiguous restraints were predicted by using the interaction analysis function in DisVis. The settings were the same as for the coarse scan, except that the rotational sampling interval was reduced to 9.72°. This process was completed after the coarse scan to determine the accessible interaction spaces that agreed with the maximum number of restraints. Next, a list of solvent-accessible residues, >40% calculated by using GETAREA, that could potentially participate in the binding interface between an RP and TPI was selected for analysis. Residues with an average interaction value of over 0.5 were considered active residues (Table S3).

The HADDOCK 2.4 web server^47,48^ was used to dock candidate RPs, either individually or in combinations, onto the TPI dimer. This process used the filtered unambiguous restraints and predicted active residues, with auto settings for passive residues. The process followed both standard (default) and modified protocols. RP inputs were fixed at the it0 stage (it0 fixed) due to the large size and slow tumbling rate of ribosomes, and the option to randomly discard 50% of interacting residues was turned off (default no discard, it0 fixed no discard) because of the limited number of predicted interacting residues in the binding interfaces. After applying four docking options, the top-ranked model (Top1) from the best cluster for each ribosome-TPI binding complex was determined (Table S4). Out of a total of 31 docked models, 29 were evaluated as high-quality based on the assessment criteria of Critical Assessment of Predicted Interactions, CAPRI,^49^ in Table S12 and the HADDOCK score (HS) of each cluster.^49,50^ Within a particular set of docking experiments (default, default no discard, it0 fixed, it0 fixed no discard), the cluster with the lowest HS and high-to-medium quality models, as determined by the HS *vs* fraction common contacts and HS *vs* interface root-mean-square deviation (rmsd) plots, was selected for HADDOCK analysis outputs. The Top4 structures from the HADDOCK outputs were further visualized using the UCSF Chimera or ChimeraX software^51^ and the MatchMaker tool under default settings with the best-alignment chain pairing to bring RPs back to their original positions in the intact ribosome. These visualizations were manually inspected for structural clashes, restraint violations, and consistency across multiple models. Finally, the binding conformation in the Top1 model was selected for subsequent electrostatic and dipole moment calculations.

### Substrate Dipole Moment Calculations

Cartesian coordinates with orthogonal axes, **a**, **c**, and **d**, originating at the C2 atom of each substrate, matrix **A** = [**a**, **c**, and **d**], were determined from models of TPI and TPI-ribosome complexes bound with DHAP and GAP. Two vectors, **C2C4** and **C2C1**, originating from carbon C2 and terminating at carbons, C4 and C1, respectively, were normalized into unit column vector coordinate files, **a** and **b**, with orthogonal axes **a**, **c**, and **d** (Table S5); **c** and **d** were determined from the cross products: **c** = **a** × **b** and **d** = **a** × **c**. The Cartesian coordinates were transformed into Q-Chem coordinates with orthogonal axes **a”**, **b”**, **c”**, and **d”**, matrix **B** = [**a”**, **c”**, **d”**] originating at the C2” atom (Table S6), and input into the Q-Chem server.^30^**a”** and **b”** are the unit column vectors of **C2”C4”** and **C2”C1”**, respectively, and **c”** and **d”** were determined from the cross products: **c”** = **a”** × **b”** and **d”** = **a”** × **c”**. A total charge of −2 were assigned to each substrate to calculate the dipole moment, **μ”**, and the Q-Chem server,^30^ accessed through the IQmol molecular viewer, was used with default settings to calculate the magnitude, in Debyes, D, and orientation of the molecular dipole moments, **μ”**, at the center of mass of each substrate. Orthogonal vectors in matrix **A** (Cartesian) and matrix **B** (Q-Chem) were used to generate rotational matrix **M** and convert **μ”** to **μ** in Cartesian coordinates using the matrix operation **M** = **A** × **B**^–1^. This operation calculates the rotational matrix, **M**, needed to interconvert matrices **A** and **B**. Finally, the dipole moment in its original coordinates, **μ**, was obtained through the equation, **μ** = **M** × **μ”**. All calculations and matrix operations were performed in MATLAB version R2020b.

### Energy Minimization and Electrostatic Calculations

The published crystal structure of *G. gallus* TPI bound to substrate analogue phosphoglycolohydroxamate, PGH, (PDB 1TPH)^52^ was edited using BIOVIA Discovery Studio (Dassault Systèmes) to replace PGH with the substrates DHAP and GAP. The edited PDB files were submitted to the YASARA Energy Minimization Server^53^ to optimize the dihedral angles and bonding networks of substrate-bound TPI. The MatchMaker tool was used to superimpose a ribosome-bound TPI dimer over the binding conformation in the Top1 models. PDB 2PQR(54) software (version 2.1.1) was used to assign charges and atomic radii to the PDB files at pH 7.0 and generate an adaptive Poisson–Boltzmann solver, APBS, (version 1.5)^31,55^ input files with AMBER as the force field. The TPI-ribosome complex was oriented within a cubic volume of ∼500 Å per side and converted to PQR format. The contribution of the substrates was not considered. Electrostatics were initially calculated using the “mg-auto” mode in a coarse grid box of 460 × 550 × 500 Å^3^ with 609 grid points in each direction to solve the PBE. The final calculation also utilized 609^3^ grid points, but in a reduced volume equal to that of the TPI-ribosome complex, to achieve a resolution of ∼0.5 Å (Figure S2B). Ionic strength was modeled as 110 mM NaCl and 10 mM MgCl_2_, based on the cross-linking buffer composition, and the solvent dielectric value was set at 78.54, with the solute dielectric value at 4.0. All other parameters of the APBS input file were kept at the default settings. The origin for each calculation was the center of mass of each substrate within the complex.

Electrostatic mappings and electric field lines were created by using visual molecular dynamics, VMD,^56^ software. An iso-surface drawing method was selected to visualize the electrostatic potentials of intact ribosomes and ribosome-TPI complexes. Blue color and an iso-value of 5 *k*_B_*T/e* were used for positive iso-contours, and red color and an iso-value of −5 *k*_B_*T/e* were used for negative iso-contours, where *k*_B_ is Boltzmann’s constant, *T* is the temperature in Kelvin, and *e* is the elementary charge. For the electric field lines, parameters included a magnitude gradient of 5.04 *k*_B_*T*(*e*Å)^−1^, a minimum length of 1 Å, and a maximum length of 125.75 Å for easier visualization. The color scale from −0.25 to 0.25 was set for the distribution of color along the field line. All resulting images were rendered directly from Tachyon, an internal, in-memory rendering component of VMD.

#### Calculations of Electric Field and Dipole-Electric Field Interaction Energy

Following the APBS calculations, electric potential data was loaded into a 75,288,843 × 3 matrix **A**, which was transformed into a 609 × 609 × 609 potential grid box, **V**, by using a custom matrix transformation program in MATLAB R2020b (Figure S3). The potential difference between **V** (*n*_*x*_ + 1, *n*_*y*_, *n*_*z*_) and **V** (*n*_*x*_ – 1, *n*_*y*_, *n*_*z*_), Δ***V*** (Figure S2B), was defined by the following equations in units of *k*_B_*T/e*
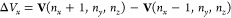
3a
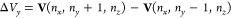
3b
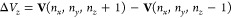
3cwhere *x*, *y*, and *z* correspond to the potential differences at the *x*, *y*, and *z* coordinates, respectively, and *n*_*x*_, *n*_*y*_, and *n*_*z*_ represent the positions in the potential grid box. The three components of the electric field, **E**, in the *x*, *y*, and *z* directions were determined by the following equations in units of *k*_B_*T*(*e*Å)^−1^
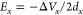
4a
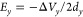
4b
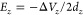
4cwhere *d*_*x*_, *d*_*y*_, and *d*_*z*_ are the grid spacings in the *x*, *y*, and *z* directions in Å. The positions in the potential grid box, **V**, were determined by the following equations
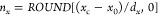
5a
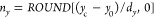
5b
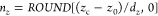
5cwhere *x*_c_, *y*_c_, and *z*_c_ are the coordinates at the center of mass of the substrates in Cartesian coordinates and *x*_0_, *y*_0_, and *z*_0_ are the new origins in the APBS coordinate system. The calculations were rounded to the nearest integer by using the *ROUND* function in Excel. The units of **E** were converted to a more commonly used unit, VÅ^–1^, with a conversion factor of 1 *k*_B_*T/e* = 0.0257 V at an absolute temperature of 298.15 K. The interaction energies, *U*, between the dipole moment of the substrates and the electric fields generated by the free TPI, *U*^TPI^, and the ribosome-bound TPI, *U*^Ribo+TPI^, were defined by the following equation^4,9^ in eV units through the conversion factor 1 eÅ = 4.8 D
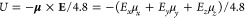
6where μ_*x*_, μ_*y*_, and μ_*z*_ represent the *x*, *y*, and *z* components of **μ**.

### Calculations of Angles between Dipole Moments and Electric Field Vectors

Both vectors were normalized to obtain unit vectors **μ**/|μ| and **E**/|*E*|. The angle, θ, between the two unit vectors was calculated by using the dot product, θ_radian = cos^–1^ (**μ**/|μ| × **E**/|*E*|). The angles, in radians, were converted into degrees by applying the equation θ = DEGREES (θ_radian). All angle calculations and conversions were performed in Excel (Microsoft).

## Theoretical Basis

### Relationship between TPI Kinetics and Molecular Dipole-Electric Field Interaction Energies

The Arrhenius equation, *k*_cat_ = *A* exp (−*E*_a_/*k*_B_*T*), where *A* is the pre-exponential frequency factor, *E*_a_ is the activation energy for the reaction, and *k*_B_ is the Boltzmann constant, was used to derive an equation describing the effect of the ribosome electric field on the *k*_cat_ of TPI-catalyzed reactions. The activation energy can be expressed as *E*_a_ = Δ*G*^†^ – Δ*G*′_DHAP_ and Δ*G*^†^ – Δ*G*′_GAP_, where Δ*G*^†^ is the Gibbs free energy of the substrate at the transition state and Δ*G*′_DHAP_ and Δ*G*′_GAP_ are the ground state Gibbs free energies of the substrates. Because both the forward and reverse reactions share the same transition state, the ratio of the rate constants is obtained by combining the Arrhenius and activation energy equations to cancel out Δ*G*^†^
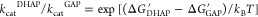
7

In this equation, *k*_cat_^DHAP^ and *k*_cat_^GAP^ are the rate constants of TPI-catalyzed reactions with substrates DHAP and GAP, respectively. The frequency factors of both substrates are assumed to be equal for simplification. The ground state Gibbs free energy for each substrate in free TPI, Δ*G*′_DHAP_^TPI^ and Δ*G*′_GAP_^TPI^, and ribosome-bound TPI, Δ*G*′_DHAP_^Ribo^ and Δ*G*′_GAP_^Ribo^, includes the EEF independent Gibbs free energy of the unbound substrate, Δ*G*_f, DHAP_ and Δ*G*_f, GAP_, and the substrate molecular dipole, **μ**, electric field, **E**, and interaction energies Δ*U*_DHAP_^TPI^ = −**μ**_DHAP_ × **E**^TPI^, Δ*U*_GAP_^TPI^ = −**μ**_GAP_ × **E**^TPI^, Δ*U*_DHAP_^Ribo+TPI^ = −**μ**_DHAP_ × **E**^Ribo+TPI^, or Δ*U*_GAP_^Ribo+TPI^ = −**μ**_GAP_ × **E**^Ribo+TPI^, when the substrate is bound to free or ribosome-bound TPI, respectively
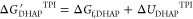
8a
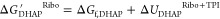
8b
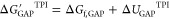
8c
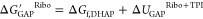
8d

Combining eqs 7 and 8a to eliminate Δ*G*_f, DHAP_ and Δ*G*_f, GAP_ yields an expression for the ratio of forward and reverse catalytic rates in terms of the interaction energies
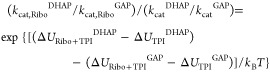
9where *k*_cat_^DHAP^, *k*_cat_^GAP^, *k*_cat, Ribo_^DHAP^, and *k*_cat, Ribo_^GAP^, are the catalytic rate constants for the substrate indicated in the absence and presence of ribosomes, respectively. This equation was used to examine the effect of the ribosome EEF on TPI activity. The left-hand term is determined by measuring enzymatic kinetics and binding affinities. Structural models are utilized to dock TPI with RPs to calculate substrate dipole moment and electric field vectors and solve for the interaction energies in the right-hand term.

Note that because subsaturating concentrations of ribosomes were used to measure the catalytic activity, the rate constant resolved is an average value, *k*_cat,avg_, resulting from free and ribosome-bound TPI
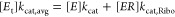
10where *k*_cat_ and *k*_cat, Ribo_ are the rates for free and ribosome-bound TPI and [*E*_t_], [*E*], and [*ER*] are the total, free, and ribosome-bound concentrations of TPI. To resolve the intrinsic catalytic rate of TPI bound to ribosomes, the distribution of free and bound TPI must be determined. Combining eq 10 with the binding equation, [*ER*] = [*E*_t_][*R*]/(*K*_d_ + [*R*]), where [*R*] is the ribosome concentration and *K*_d_ is the apparent dissociation constant between ribosomes and TPI, yields
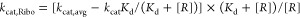
11

Rewriting eq 11 for each substrate allows calculation of the intrinsic catalytic rate for substrate-bound TPI bound to ribosomes
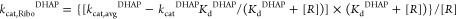
12a
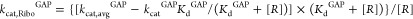
12bwhere *k*_cat_^DHAP^ and *k*_cat_^GAP^ are the rate constants measured for the substrates DHAP and GAP, respectively, in the absence of ribosomes, *k*_cat,avg_^DHAP^ and *k*_cat,avg_^GAP^ are the rate constants measured in the presence of ribosomes, and *K*_d_^DHAP^ and *K*_d_^GAP^ are the TPI-ribosome dissociation constants for DHAP- and GAP-bound TPI.

## Results

### TPI Interacts with Ribosomes

To validate the RAMBO effect, a structural interaction between TPI and ribosomes must be identified and characterized. NMR spectroscopy has proven to be a highly effective method for probing weak protein–protein interactions at the atomic level.^57−60^ In-cell NMR studies^33^ showed that ribosomes exhibit specific transient, micromolar affinity, and quinary^61−64^ interactions with proteins. Subsequent *in vitro* work^17,65^ revealed that intact ribosomes broadened protein NMR spectra in a manner similar to what was observed in-cell, providing an assay for mimicking quinary interactions under cytosol-like conditions. A mammalian ribo-interactome study,^13^ identified TPI as being associated with ribosomes, along with other glycolytic enzymes, but no direct evidence for an interaction was provided. A search of the Biological Magnetic Resonance Bank showed that among glycolytic enzymes, only phosphoglycerate kinase,^66^ phosphoglycerate mutase,^67^ and TPI from *G. gallus*(32) have had NMR backbone nuclei assigned, making TPI a good candidate for this study.

The ribosomes used in this study were from *E. coli* MRE600. While structural differences between prokaryotic and eukaryotic ribosomes are well-documented^68,69^ and protein binding to these macromolecules are likely to involve distinctly different interaction surfaces, the point of this study is to investigate the general concept of whether coupling between macromolecular EEFs and the molecular dipole of an enzymatic substrate can interact to modulate catalytic activity. Prokaryotic ribosomes provide an ideal model because more comprehensive structural information is available for prokaryotic ribosomes than for eukaryotic ribosomes, allowing for more precise calculations of the EEF.

**Figure 2:**
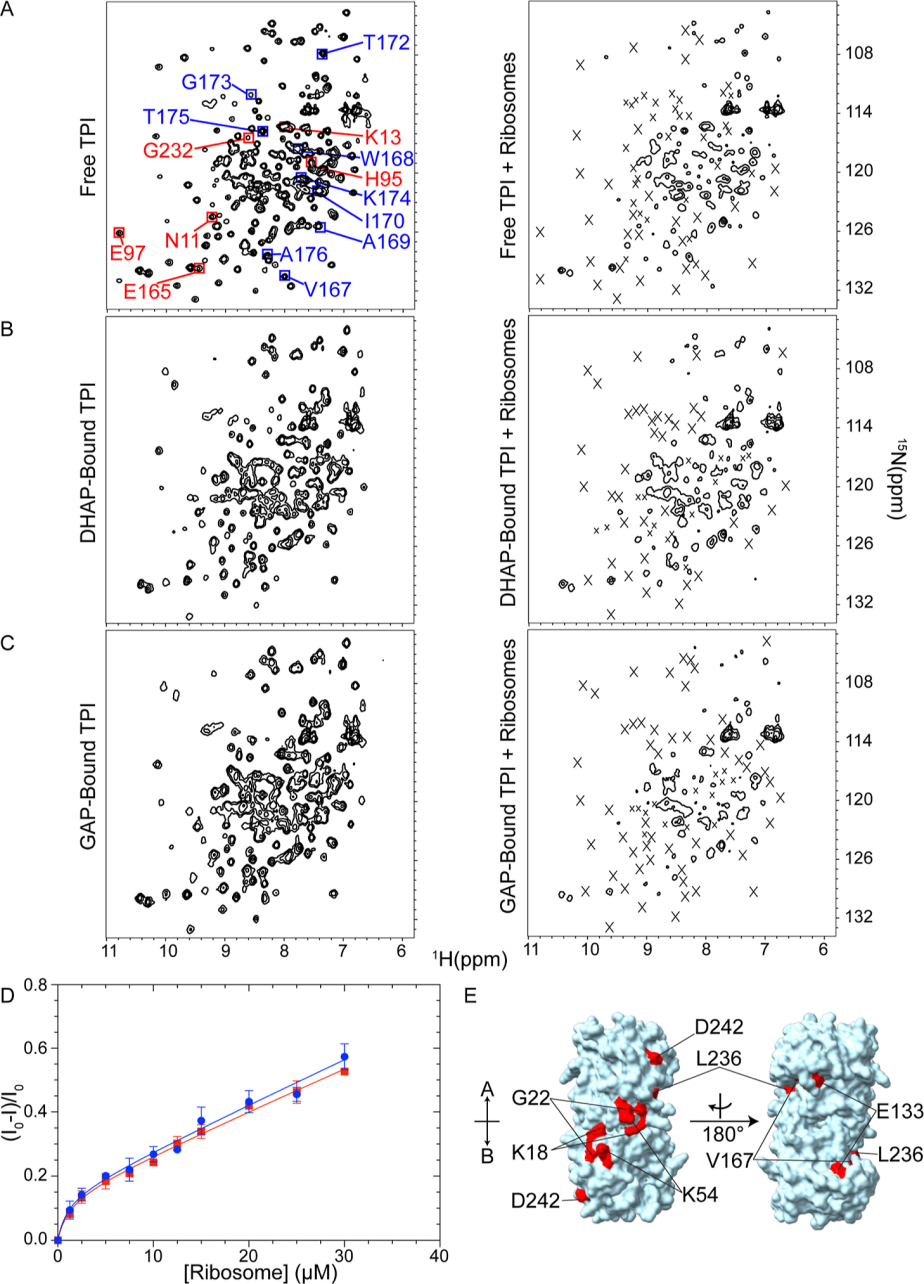
Interaction between TPI and ribosomes. (A) ^1^H–^15^N HSQC spectrum of 50 μM [U–^15^N]-TPI without (left) and with (right) 2 μM ribosomes. Left, active site residues are highlighted in red (G171, S211, and G233 were not observed), and residues of dynamic loop 6 are highlighted in blue (P166 and G171 were not observed). Assignments were made using BMRB entry 15064.^32^ (B) ^1^H–^15^N HSQC spectra of 50 μM DHAP-bound [U–^15^N]-TPI (5 mM DHAP) without (left) and with (right) 2 μM ribosomes. Assignments were based on BMRB entry 15065.^32^ Cross peaks broadened due to interaction with ribosomes are marked with an X. (C) ^1^H–^15^N HSQC spectra of 50 μM GAP-bound [U–^15^N]-TPI (1 mM GAP) without (left) and with (right) 2 μM ribosomes. Assignments were made using BMRB entry 15065.^32^ Cross peaks broadened due to interaction with ribosomes are marked with an X. All spectra were processed at the same contour level. (D) Binding curves for substrate-bound TPI. Normalized intensity difference, (*I*_0_ – *I*)/*I*_0_, of the ^1^H–^15^N HSQC spectra amide proton envelope of DHAP-bound TPI (blue) and GAP-bound TPI (red) increases with an increasing ribosome concentration. TPI was at 2.5 μM, DHAP and GAP were at 5 and 1 mM, respectively. Error bars show the mean ± standard deviation (SD) from at least three independent experiments. (E) Residues involved in quinary interactions (red) due to the presence of intact ribosomes are mapped onto the molecular surface of TPI (PDB entry 8TIM). TPI is oriented to show the front (left) and the back (right) views, with subunit A at the top and subunit B at the bottom.

Purified TPI from *G. gallus* was prepared for NMR analysis (Figure S4). Heteronuclear single-quantum coherence, ^1^H–^15^N HSQC, spectra were collected for free and substrate-bound uniformly labeled, [U–^15^N], TPI in the absence and presence of 2 μM ribosomes (Figure 2). A total of 226 cross peaks were resolved for the free TPI spectrum. Notably, these included active site residues N11, K13, H95, E97, E165, and G232, and those comprising dynamic loop 6, V167, W168, A169, I170, T172, G173, K174, T175, and A176 (Figure 2A, left).^19,32,70^ TPI cross peak intensities uniformly broadened as the ribosome concentration increased (Figure 2A, right), providing clear evidence of a micromolar affinity quinary interaction between TPI and the ribosomes. Extreme broadening of selected peaks (Figure S5) implies that those residues may be part of or close to the interaction surface.

Upon addition of substrates, the TPI ^1^H–^15^N HSQC spectra displayed extensive but similar chemical shift changes relative to free TPI (Figure 2B,C, left). This was expected given that the binding sites for DHAP and GAP are the same. There was 97% agreement (213 of 220 residues) between the DHAP-bound TPI spectrum and that of chicken TPI bound to intermediate analogue 2-phosphoglycolate,^32^ and 94% agreement (207 out of 220 residues) for GAP-bound TPI.^32^ Because the spectra represent both catalytically active (substrate-bound) and noncatalytic TPI, the near identical assignments of cross peaks for both substrate-bound spectra indicate that an equilibrium between DHAP- and GAP-bound TPI was reached during the hour long period of data acquisition. When 2 μM ribosomes were added to both free and substrate-bound TPI most of the cross peaks were extensively broadened due to the quinary interactions between ribosomes and TPI (Figure 2B,2C, right), implying that the quinary complex persisted.

To estimate the affinity of the TPI-ribosome interaction, substrate-bound TPI was titrated with ribosomes (Figure 2D). The integrated density of the ^1^H–^15^N HSQC spectra amide proton envelope from 6.30 to 10.54 ppm was used to assess the extent of binding (Figure S6). The intensity of the TPI envelope rapidly decreased as intact ribosomes were added. Analysis resolved a specific binding site with apparent dissociation constants of ∼1 μM for each substrate-bound TPI (Table 1). A monotonic decrease in intensity at higher ribosome concentrations was consistent with weaker, nonspecific interactions. Mapping the interaction surface onto the TPI structure revealed patches that were largely confined to one side of the TPI dimer (Figure 2E). Note that because subsaturating concentrations of substrates were used in the titrations, the binding affinity estimated by NMR for substrate-bound TPI reflects the overall interaction of free and catalytically active species with ribosomes and thus represents an upper limit to *K*_d_.

**Table 1:** TPI-Ribosome Binding Parameters^a^^,^^g^

substrate^b^	*B*_max_^c^	NS^d^	*K*_d_ (μM)^e^	*R*^2^^f^
DHAP	0.15 ± 0.02	0.014 ± 0.001	1.05 ± 0.51	0.98
GAP	0.14 ± 0.02	0.013 ± 0.001	1.21 ± 0.56	0.99

a Binding curves were analyzed using the total binding equation (*I*_0_ – *I*)/*I*_0_ = *B*_max_ [*R*]/(*K*_d_ + [*R*]) + NS [*R*], where *I* and *I*_0_ are the TPI amide envelope peak intensity with and without ribosomes, respectively.

b [DHAP] and [GAP] were 5 and 1 mM, respectively.

c *B*_max_ is the maximum specific binding ratio.

d NS is the slope of nonspecific binding.

e *K*_d_ is the apparent dissociation constant for the ribosome-TPI interaction.

f *R*^2^ is the coefficient of determination.

g Values are the mean ± standard error of the mean (SEM).

### Ribosomes Alter TPI Activity

TPI kinetics have been extensively studied for over half a century using an assay^71^ that is still widely employed today. The assay couples GAP dehydrogenase, GAPDH, with NAD^+^ when DHAP is used as the substrate or alpha-glycerol 3-phosphate dehydrogenase, GOPDH, with NADH when GAP is used as the substrate and follows the production or oxidation of NADH spectrophotometrically. However, GAPDH was identified as an RNA-binding protein^72^ and may therefore affect TPI activity by binding to ribosomes. To avoid this potential interference, a direct steady-state kinetic assay using NMR spectroscopy^16^ was employed. In this assay, 1D proton NMR spectra of the substrates were collected in pseudo-2D mode with time as the second dimension (Figure S7A). Forward and reverse reactions were quantified by monitoring the increase or decrease in the volume of the substrate peak over time (Figure S7B). Phosphate buffer at pH 7.5 was used to eliminate background signals that may overlap and obscure substrate proton cross peaks, and the assay temperature was fixed at 290 K to slow the reaction. Less than 10% of the starting substrate concentration was lost during the reaction dead time (Figure S7C,D). A ribosome concentration of 1 μM was used to minimize nonspecific binding.

**Figure 3:**
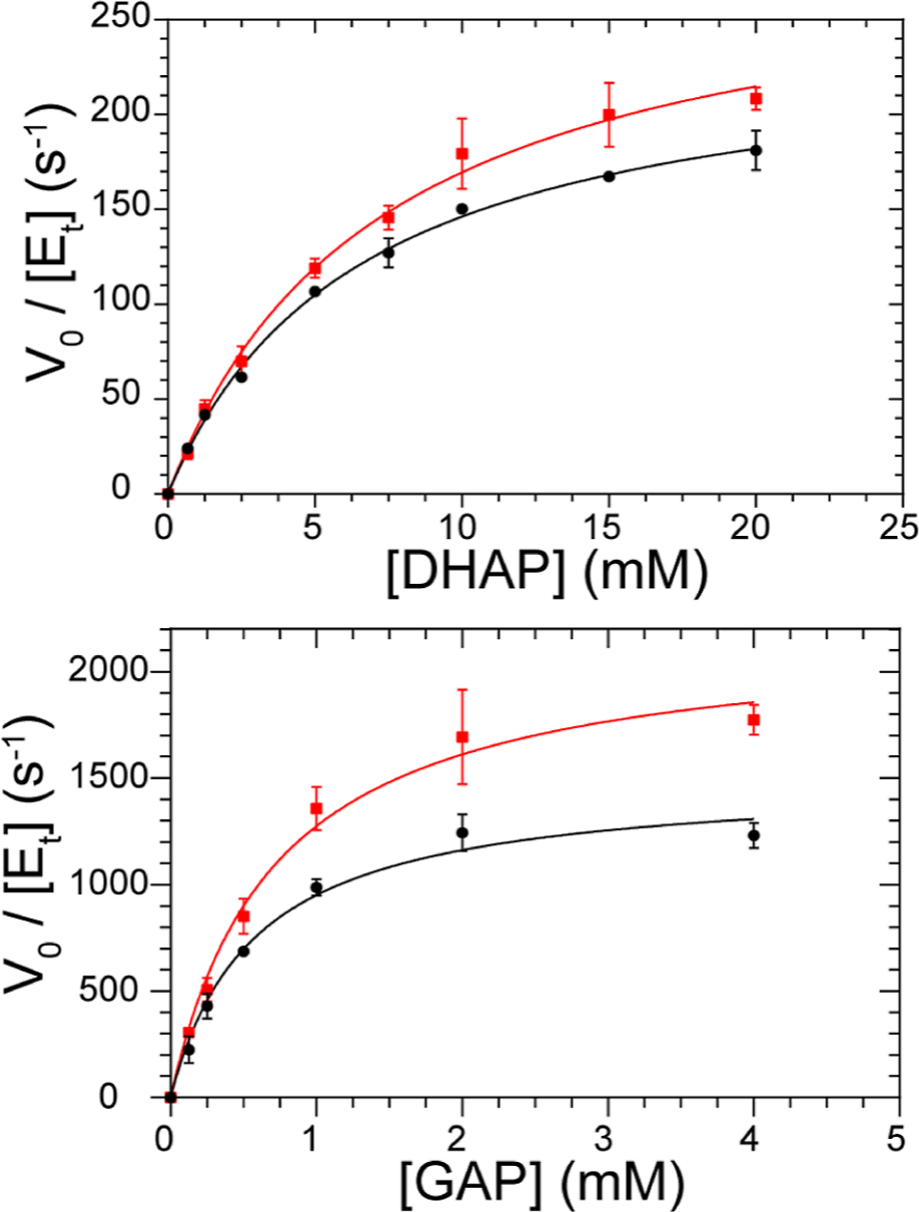
Ribosomes affect TPI kinetics. Saturation kinetic curves without (black) and with (red) 1 μM ribosomes using DHAP (top) and GAP (bottom) as substrates. Data were fit to the Michaelis–Menten equation (eq 1) using GraphPad Prism 9. Error bars show the mean ± SD from three independent trials.

Kinetic parameters of *K*_M_ = 6.5 mM and *k*_cat_ = 240 s^–1^ were resolved for the DHAP saturation curve, and *K*_M_ = 0.57 mM and *k*_cat_ = 1500 s^–1^ for the GAP saturation curve (Figure 3 and Table 2). The kinetic parameters are in general agreement with published kinetic data.^73−76^ This agreement is notable considering that the assay was shifted from conditions typically used, such as a decreased temperature leading to slower substrate diffusion and the likelihood of competitive binding by inorganic phosphate at the active sites facilitated by the ability of loop 6 to grip phosphate.^77^ In the presence of 1 μM ribosomes, *k*_cat_ increased by 22 and 46% when DHAP and GAP, respectively, were bound to TPI. The differences are statistically significant as determined from the independent samples *t*-test (*p* < 0.001) and Cohen’s *d* measures, which calculate the differences in the mean experimental values (*d*_DHAP_ = 1.04 and *d*_GAP_ = 2.00). However, the 12 and 26% increase in *K*_M_ resolved from these data were determined to be statistically insignificant (*p* > 0.05, *d*_DHAP_ = 0.26 and *d*_GAP_ = 0.42).

**Table 2:** Steady-State Kinetic Parameters with and without Ribosomes

substrate	no ribosomes			1 μM ribosomes		
	*K*_M_ (mM)	*k*_cat_ (s^–1^)	*k*_cat_*/K*_M_ (mM^–1^ s^–1^)	*K*_M_ (mM)	*k*_cat_ (s^–1^)	*k*_cat_/*K*_M_ (mM^–1^ s^–1^)
DHAP^a^	6.5 ± 0.4^b^	240 ± 6^c^	37	7.3 ± 0.7^b^	290 ± 12^c^	40
GAP^a^	0.57 ± 0.06^b^	1500 ± 50^c^	2600	0.72 ± 0.09^b^	2200 ± 90^c^	3000
DHAP^d^	0.97	430	450	N/A		
GAP^d^	0.47	4300	9100			
GAP^e^	0.46 ± 0.06	2000 ± 60	4300	N/A		
DHAP^f^	0.59 ± 0.05	340 ± 5	580	N/A		
GAP^f^	0.29 ± 0.02	3200 ± 100	11000			

a This work: 17 °C, pH 7.5, *G. gallus* TPI, direct NMR assay. Note: Values are the mean ± SEM.

b Differences between *K*_M_ in the absence and presence of ribosomes are statistically insignificant (*p* > 0.05, *d*_DHAP_ = 0.26 and *d*_GAP_ = 0.42).

c Differences between *k*_cat_ in the absence and presence of ribosomes are statistically significant (*p* < 0.001, *d*_DHAP_ = 1.04 and *d*_GAP_ = 2.00).

d 30 °C, pH 7.27 for DHAP, pH 7.42 for GAP, *G. gallus* TPI, coupled assay.^74^

e 25 °C, pH 7.6, *G. gallus* TPI, coupled assay.^73^

f 25 °C, pH 7.5,*G. gallus* TPI, coupled assay.

The tight dimerization constant estimated for TPI, 10^–12^–10^–16^ M,^78,79^ which is 3–6 orders of magnitude below the concentration of TPI used in these experiments; therefore, dimeric TPI is the sole species, and the change in activity cannot be attributed to ribosome-mediated alteration of the monomer–dimer equilibrium. Furthermore, based on the protocol used to purify intact ribosomes and supporting evidence from analytical methods, the ribosome preparations used in our experiments were free of chaperones and other ribosome-associated factors (see Materials and Methods). Thus, the increase in the rate of catalysis of TPI was attributed directly to the binding interaction between dimeric TPI and the ribosome.

### Ribosomal Binding Sites for TPI

**Figure 4:**
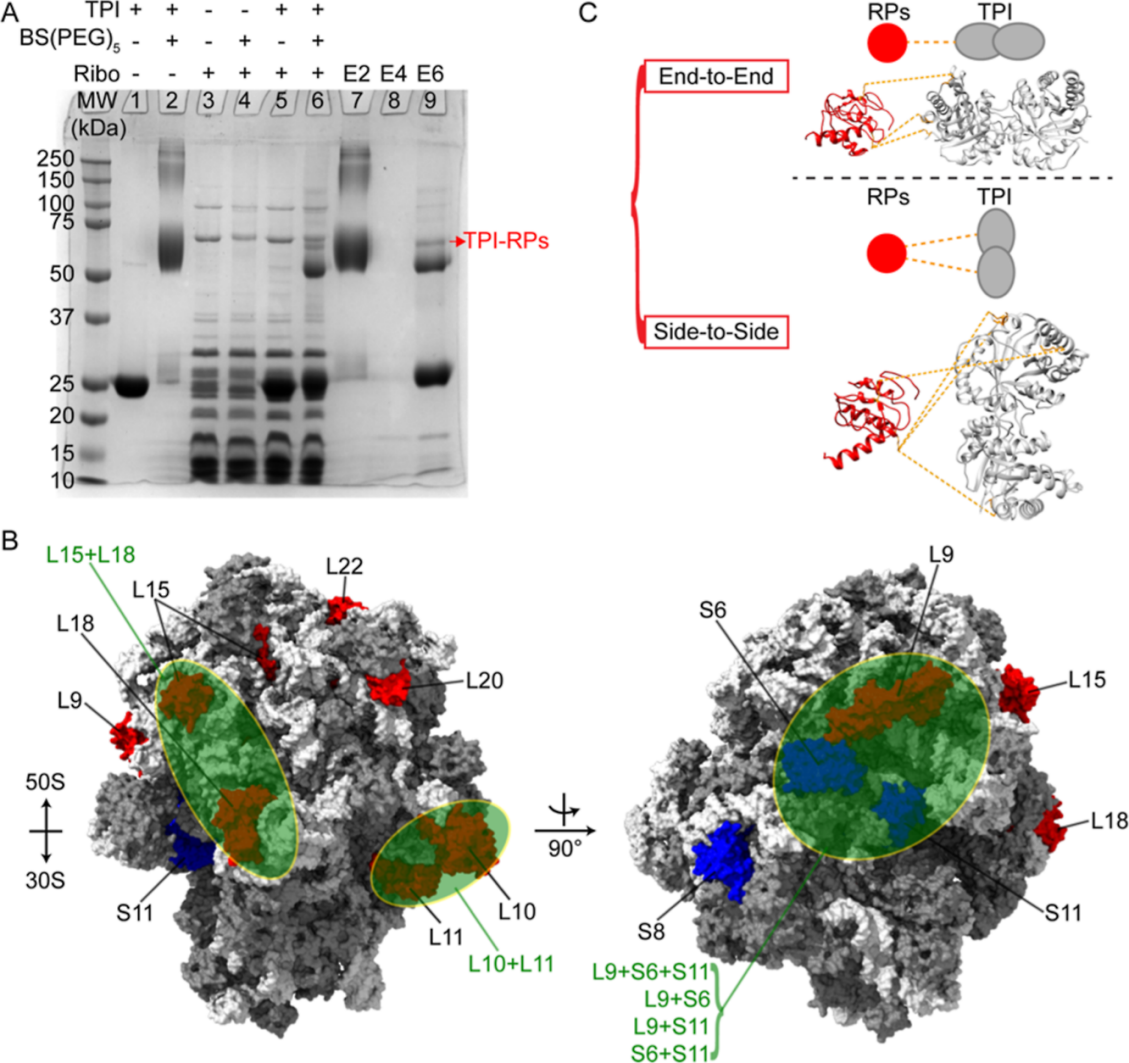
Putative TPI-ribosomal binding sites. (A) SDS-PAGE of cross-linking reactions. Purified TPI without (lane 1) with (lane 2) 2 mM BS(PEG)_5_, purified ribosomes without (lane 3) and with (lane 4) 2 mM BS(PEG)_5_, TPI and ribosomes without (lane 5) and with (lane 6) 2 mM BS(PEG)_5_, and the elution of cross-linked products from lane 2 (E2, lane 7), lane 4 (E4, lane 8), and lane 6 (E6, lane 9) after recapturing cross-linked His-tagged TPI. One band, denoted as TPI-RPs, is indicated in lane 9 was excised for MS analysis. (B) Possible TPI binding sites on the ribosome. Surface diagrams of intact ribosomes (PDB entry 5UYK, rRNA in light gray and RPs in dark gray) are shown with RPs L9, L10, L11, L15, L18, L20, and L22, from the 50S subunit, highlighted in red, and S6, S8, and S11, from the 30S subunit, highlighted in blue. Green ellipses indicate possible binding sites involving multiple RPs. (C) Two possible binding modes for TPI dimers (PDB entry 8TIM, gray) and L10 (red): end-to-end, in which only one subunit of the TPI dimer contacts the RP and side-to-side, in which both subunits contact the RP. Intermolecular cross-links are labeled as orange dashed lines.

Chemical XL-MS analysis was used to identify possible TPI binding sites on intact ribosomes. Three types of homobifunctional amine-to-amine cross-linkers were used with space arms ranging from 11.4 to 35.8 Å. Only BS(PEG)_5_ with a 21.7 Å space arm produced analyzable results (Figure 4A). The purified His-tagged 25 kDa TPI monomer is shown in Figure 4A, lane 1. When treated with 2 mM BS(PEG)_5_, TPI migrated as a cross-linked dimer with a MW of ∼50 kDa and several larger oligomers (Figure 4A, lane 2). Cross-linking ribosomes resulted in reduced band intensity at 70 and 100 kDa, eliminated a band at 25 kDa, and generated a faint band just under 100 kDa (Figure 4A, lane 4). BS(PEG)_5_ cross-linking between TPI and ribosomes yielded one major band at ∼65 kDa and several faint bands at higher MWs (Figure 4A, lane 6). To confirm that the band at 65 kDa contained cross-linked TPI and ribosomes, the reactions in lanes 2, 4, and 6 were loaded separately onto Ni-NTA beads under denaturing conditions to capture and elute His-tagged TPI. Lanes 2 and **7** (**E2**) were identical, confirming that only TPI was present. No TPI was evident in lane 8 (**E4**), which contained only ribosomes and BS(PEG)_5_. Lane 9 (**E6**) showed cross-linked TPI monomers, dimers, and tetramers, the band at 65 kDa, and several weak low MW bands. Because the 65 kDa band contains cross-linked TPI dimers, the most likely cross-linked RPs, are restricted to those with molecular weights less than ∼20 kDa (Table S7). RPs L7/L12 were excluded from the search because no changes were evident in [U–^15^N]-ribosome spectra upon TPI titration^16,80^ (Figure S1).

The 65 kDa band was excised and enzymatically digested in gel prior to analysis using a bottom-up proteomics strategy.^28^ To control for potential false positives, bands of cross-linked TPI, cross-linked RPs, and mock-loaded gel at ∼65 kDa were also isolated and processed as previously described.^38^ A typical MS spectrum is shown in Figure S8. RP candidates and TPI were input to a database for pLink2.0^39^ to search for intermolecular cross-links and identify solvent accessibility of cross-linked residues (Table S1). Ten RPs, L9, L10, L11, L15, L18, L20, L22, S6, S8, and S11 were identified as potential TPI-binding partners and highlighted on a model of intact ribosomes in Figure 4B. All ten candidates are concentrated in either the stalk region where elongation factor binds, the exit site of the ribosomal tunnel, the central protuberance of the 50S subunit, or the platform of the 30S subunit. The proximity of RPs in combination with the size of the TPI dimer (72 × 38 × 42 Å^3^, PDB entry 8TIM) identified 6 additional combinations of RPs, L10 + L11, L15 + L18, L9 + S6, S6 + S11, L9 + S11, and L9 + S6 + L11, that may also serve as possible binding sites for TPI despite the absence of chemical cross-links (Figure 4B). Note that it is not unexpected that multiple RPs may be cross-linked because the high concentrations of TPI and ribosomes used in the cross-linking reaction, 100 and 10 μM, respectively, increased the likelihood of cross-links resulting from nonspecific binding (Figure 2D). In summary, 29 distinct cross-links were identified by using MS and tandem MS/MS search and match, and all cross-linked peptides agree within 3 ppm with the theoretical masses. Because TPI is a homodimer, it cannot be determined from the primary structure whether one or both monomers bind to RPs. Two possible binding modes, end-to-end and side-to-side, in which one or both monomers interact with the RPs are shown in Figure 4C.

### TPI-Ribosome Model Complexes

**Figure 5:**
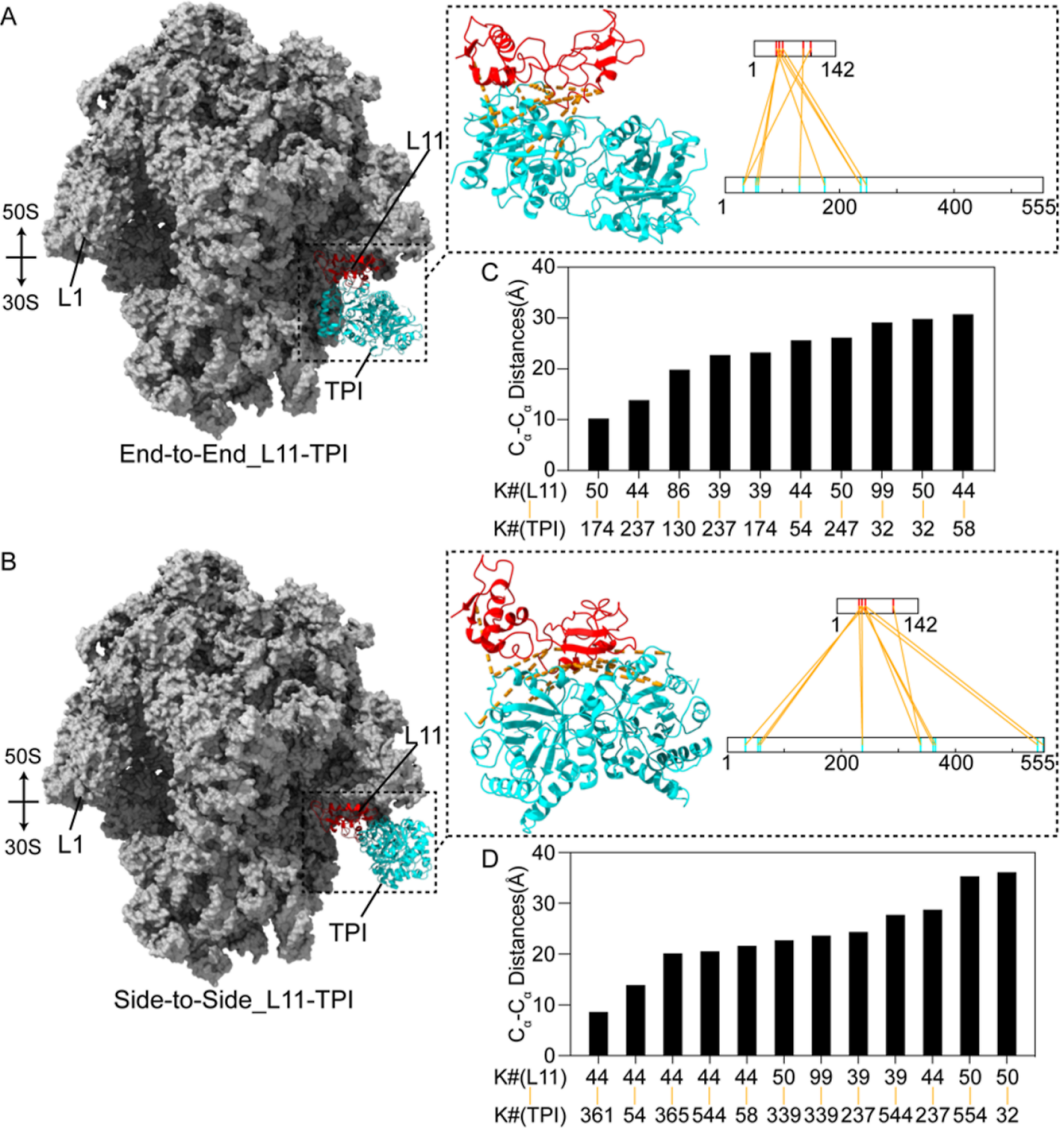
Structural model of TPI-ribosome interaction. Representative models showing end-to-end (A) and side-to-side (B) binding modes for the RP L11-TPI dimer interaction. RPs L1 and L11, and 50S and 30S subunits are shown for orientation. Dashed insets display ribbon diagrams of L11 (red) and the TPI dimer (cyan) with modeling distance constraints indicated by orange dashes (left), and cross-link network maps^82^ for L11 residues 1–142 and TPI residues 1–248 as subunit A and 308–555 as subunit B. (right) The image was prepared by using the MatchMaker tool of UCSF ChimeraX or Chimera^51,83^ for structural superimposition, matching, and replacing L11 of the intact ribosome. C_α_-C_α_ distances between each pair of cross-linked lysines are illustrated in ascending order for end-to-end (C) and side-to-side (D) modes. Lysine residues, K#, are indicated for L11 and TPI.

To assess the ribosome-dependent field-dipole interaction energy, each of the 31 possible TPI-ribosome interactions was modeled, and the resulting conformations were used to calculate substrate dipole moments and electric field vectors. Published structures for TPI (PDB 8TIM) and *E. coli* 70S ribosome (PDB 5UYK)^81^ were used as starting models for High Ambiguity Driven protein–protein DOCKing (HADDOCK)^29^ and DisVis^45^ analysis. DisVis was used to visualize and quantify distance restraints between the macromolecular complexes. To generate high-quality models of TPI dimer-RP interacting surfaces, the cross-link restraints and predicted interacting residues from DisVis analysis were imported to HADDOCK and analyzed using the standard (default) protocol with three modified docking settings. A model of the interaction between RP L11, which had the greatest number of cross-links, and the TPI dimer is shown in two possible binding modes in Figure 5. The distance restraints are satisfied in the modes: end-to-end binding mode C_α_-C_α_ restrained distances were 23.1 ± 2.2 Å, and side-to-side binding mode C_α_-C_α_ restrained distances were 23.6 ± 2.3 Å (Figure 5C,D). To generate models of ribosome-TPI complexes, the best docked models of the TPI-RP complexes from HADDOCK analysis were structurally superimposed over the original positions of RPs within intact ribosomes. Model complexes were subsequently generated by replacing the original RP with the RP input of the docked model. The overall rmsd of the structural superimpositions across all atom pairs was 0.37 ± 0.14 Å.

To position substrates within the catalytic sites of ribosome-bound TPI, a substrate analogue phosphoglycolohydroxamate, PGH, was modified to the native substrates DHAP or GAP. Edited models of TPI from PDB entry 1TPH were subjected to energy minimization using the YASARA Energy Minimization Server^53^ to optimize the bonding network around the catalytic sites and the dihedral angles of the introduced substrates (Figure S9). Compared to the PGH-bound TPI model, the bonding networks of GAP exhibited greater movements, particularly in relation to the catalytic base E165, than those of DHAP. This result is expected given the similarity in chemical structure between DHAP and PGH.^19,32^ The energy-minimized substrate-bound TPI models were used to replace apo-TPI in the TPI-ribosome complexes obtained by using structural superimposition. The resulting rmsd values of DHAP-bound TPI and GAP-bound TPI across all atom pairs were 1.29 ± 0.10 and 1.31 ± 0.10 Å, respectively. The matches were consistent with the flexibility of loop 6, which moves approximately 7 Å at the tip, and the global structural variation from an unbound to a bound state,^19,75^ both of which contribute to larger rmsd values compared to structural superimposition.

### Calculation of Substrate Dipole Moments

The dipole moments of substrate bound to TPI-ribosome complexes were calculated by using Cartesian coordinates from models of substrate-bound TPI and substrate-bound TPI-ribosome complexes (Figure S2A). Substrates situated in the first and second active sites of the TPI dimer were designated as GAP1, DHAP1, GAP2, and DHAP2, respectively, with the numbers indicating which active site was bound. The *x*, *y*, and *z* components of each dipole moment, originating at the center of mass, were calculated for the four substrate-TPI complexes bound to each potential site on the ribosome (Table S8). The average dipole magnitude in Debye, D, for each complex was 20.911 ± 0.001 for GAP1, 20.145 ± 0.001 for GAP2, 19.514 ± 0.001 for DHAP1, and 21.725 ± 0.001 for DHAP2. The dipole moment is highly sensitive to molecular geometry and substrates undergo a rearrangement of chemical bonds when bound to the active sites. The difference in the dipole moments calculated for the same substrate at the two active sites, particularly between DHAP1 and DHAP2, is also because the TPI homodimer is not perfectly symmetric (PDB 8TIM).

This asymmetry was quantified by calculating the rmsd, the average distance between paired atoms in superimposed subunits. rmsd calculations utilize atomic coordinates from energy minimization, a process that can result in distinct local energy minima for each active site once the system reaches a global minimum. The substrate-bound monomeric subunits display a comparable increase in symmetry relative to free TPI (Table S9). When only active site residues are compared, the overall symmetry of GAP-bound TPI shows an increase in symmetry comparable to that observed for the overall molecule, while that of DHAP-bound TPI shows much larger deviations (Table S9), consistent with greater asymmetry between the two active sites. This affects the final substrate geometry and results in large deviations in the calculated dipole moment for substrate at each active site. These observations are consistent with the weaker binding observed for DHAP, relative to GAP, which may result in more flexibility and subsequent divergent active site geometries.

### Calculation of Electric Field Vectors for TPI-Ribosome Complexes

The electrostatic potentials of the model TPI-ribosome complexes were calculated by solving the Poisson–Boltzmann equation, PBE.^3^ The PBE describes the relationship between the electrostatic potential, the dielectric constants of the solute and solvent, solvent ionic strength, the charge density distribution, and ion accessibility to the solute interior. PDB 2PQR(54) software was used to assign charges and atomic radii at pH 7.0 and generate APBS^31^ input files with AMBER as the force field^84^ to solve for the electric potentials of the TPI-ribosome complexes. The electric field describes the rate of change in potential with distance and points toward the negative potential.^3^ Electrostatic potentials were calculated by using the finite difference method in combination with multigrid and parallel focusing algorithms^31,85^ (Figure S2B). This process of discretization reduces the calculation of electric fields from continuous partial derivatives to approximations using direct potential differences over small grid spacings, where the electric field is assumed to be uniform.

Electric field calculations were applied to all model complexes of free and ribosome-bound TPI (Table S10). To minimize errors from charge discretization, the APBS grid settings, including the lengths of coarse and fine grid boxes and the grid spacings, were kept uniform for all calculations. Different binding modes result in particular centers of mass for each complex model, which require different APBS origins, and each boundary condition defined by the initial (coarse) calculation results in different arrangements of charge discretization for the fine solution. These dynamic binding conformations lead to distinct magnitudes and orientations for the molecular dipoles and electric fields calculated for each binding conformation (Table S11).

### TPI Binding to Ribosome Protein L11 Promotes the RAMBO Effect

With experimental and calculated estimates of substrate binding constants, TPI reaction rates, substrate dipole, and electric field vectors, the effect of the ribosome electric field on TPI activity was evaluated in terms of the RAMBO effect proposed in eq 9. Substrate dipole-electric field interaction energies for free and ribosome-bound TPI are tabulated in Table S12, and differences in substrate interaction energies between free and bound TPI for all model complexes are listed in Table S13. Equations 12a and 12b were used to calculate the catalytic activity of fully bound TPI (Table 3). The models that are most consistent with dipole-EEF enhancement of catalytic activity will have the same solution for both sides of the equation. The ratio of rate constants presents a single solution of 0.71 ± 0.30. This value, less than 1, is consistent with the greater enhancement in activity observed for GAP as the substrate versus that observed for DHAP.

**Table 3:** Kinetic Rate Constant Ratio and Interaction Energy Differences for TPI-RP Model Complexes^f^

RPs	possible binding modes	right-hand term of eq 9			left-hand term of eq 9	Cohen’s *d*^e^
		1st active site^a^	2nd active site^b^	average of two sites^c^	*k*_cat_ ratio^d^	
L9	end-to-end	1.19	2.09	1.64 ± 0.45		0.68
	side-to-side	0.88	0.97	0.92 ± 0.04		0.16
L10	end-to-end	1.06	0.86	0.96 ± 0.10		0.18
	**side-to-side**	1.28	1.04	1.16 ± 0.06		0.33
L11	**end-to-end**	0.62	0.68	0.65 ± 0.03	0.71 ± 0.30	–0.04
	**side-to-side**	0.72	0.92	0.82 ± 0.10		0.08
L15	end-to-end	0.99	0.88	0.93 ± 0.06		0.16
	side-to-side	1.59	0.90	1.25 ± 0.34		0.39
L18	end-to-end	0.88	1.21	1.04 ± 0.17		0.25
	**side-to-side**	1.03	0.88	0.96 ± 0.07		0.18
L20	end-to-end	0.80	0.89	0.84 ± 0.04		0.10
	**side-to-side**	1.05	1.02	1.03 ± 0.01		0.24
L22	end-to-end	1.13	0.99	1.06 ± 0.07		0.25
S6	end-to-end	1.24	0.99	1.11 ± 0.13		0.29
S8	end-to-end	0.97	0.77	0.87 ± 0.10		0.12
	side-to-side	1.05	0.87	0.96 ± 0.09		0.18
S11	end-to-end	0.47	0.86	0.66 ± 0.19		–0.03
	side-to-side	1.55	1.18	1.37 ± 0.18		0.48
L10 + L11	end-to-end	1.12	1.11	1.12 ± 0.03		0.30
	**side-to-side**	1.03	1.01	1.02 ± 0.01		0.23
L15 + L18	end-to-end	1.00	0.82	0.91 ± 0.09		0.15
	side-to-side	0.92	0.95	0.93 ± 0.02		0.16
L9 + S6	end-to-end	1.05	1.15	1.10 ± 0.05		0.29
	side-to-side	0.33	0.78	0.56 ± 0.23		–0.11
S6 + S11	end-to-end	1.03	0.86	0.94 ± 0.08		0.17
	side-to-side	0.71	1.26	0.98 ± 0.28		0.20
L9 + S11	end-to-end	0.93	1.01	0.97 ± 0.04		0.19
	side-to-side	1.31	0.93	1.12 ± 0.19		0.30
L9 + S6 + S11	end-to-end	0.90	0.88	0.89 ± 0.01		0.13
	side-to-side	0.83	1.11	0.97 ± 0.14		0.19
	side-to-side	2.49	1.06	1.77 ± 0.71		0.77

a Interaction energy differences at the first active site of TPI for DHAP1 and GAP1, with and without ribosome binding, were substituted into right-hand term of eq 9.

b Interaction energy differences at the second active site of TPI for DHAP2 and GAP2, with and without ribosome binding, were substituted into right-hand term of eq 9.

c Average of calculated exponential values from the two active sites were used because both sites are functional in the TPI dimer.

d *k*_cat,Ribo_ were obtained when *K*_d_ and 1 μM of ribosome concentration used for substrate titration were substituted into eqs 12a and 12b.

e Cohen’s *d* measures were used to measure the mean differences between the average of the two active sites for each structural model and the *k*_cat_ ratio. Cohen’s *d* measures of 0.2, 0.5, and 0.8 correspond to small, medium, and large differences in the mean experimental values, respectively. A negative Cohen’s *d* means that the average values of the two sites are lower than the *k*_cat_ ratio, whereas a positive Cohen’s *d* means that the average values are higher than the *k*_cat_ ratio.

f Values are expressed as the mean ± SEM.

**Figure 6:**
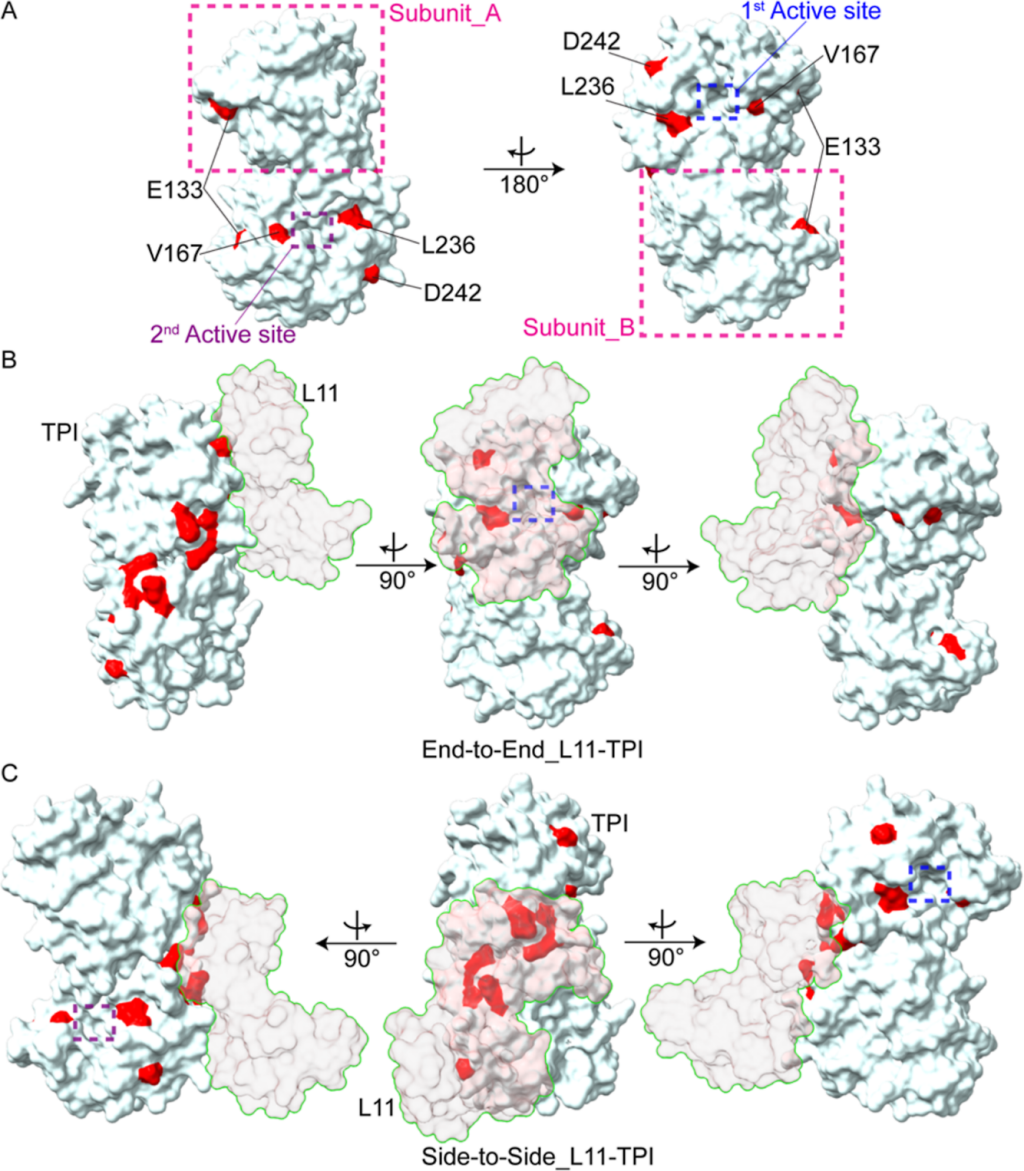
Quinary interaction surface modeling of a TPI dimer bound to RP L11. (A) TPI dimer is oriented to display views rotated 180° with respect to each other. Binding interface residues highlighted in Figure S5 are in red. (B) Residues L236 and D242 from subunit A contribute to the binding interface between L11 and TPI in the end-to-end mode. (C) Residues K18, G22, and K54 from both subunits contribute to the binding interface between L11 and TPI in side-to-side mode. TPI coordinates are from PDB entry 8TIM. TPI catalytic site is indicated by a dashed blue box for the same orientation in A, B, and C. End-to-end configuration occludes one active site (B) and the side-to-side configuration results in no steric occlusion of the active sites (C).

To identify the RP-TPI complex that conforms with the phenomenological equation and mediates EEF enhancement of catalytic activity, three criteria were used: first, each average value for the two sites was evaluated for energetic compliance with the model; second, Cohens’*d* measures were used to assess the difference between each average value and the *k*_cat_ ratio; and third, TPI-ribosome quinary interaction surfaces were examined to identify viable structural conformations. The values cited in Table 3 represent the mean for TPI sites 1 and 2 calculated from the right-hand terms of eq 9 for a TPI dimer bound in a given configuration and not between different binding configurations. About half of the average values were close to 1, indicating a similar contribution from the ribosome EEF on the interaction energies for DHAP and GAP, and about one-quarter exceeded 1.1, suggesting a larger interaction energy for DHAP than for GAP. The Cohen’s *d* measures were ≤0.2 for 18 of the 31 models, indicating small differences, and 4 had values ≤ 0.1 (Table 3). Finally, surface mapping of quinary interaction identified six viable structural conformations (Table 3, bold): side-to-side binding of L10, L18, L20, and L10 + L11 (Figure S10), as well as side-to-side and end-to-end binding of L11 (Figure 6).

L20 and L10 + L11 were eliminated because the right-hand terms for both sites and their average values were very close to 1.00. This condition indicates that either *U*_Ribo+TPI_^DHAP^ – *U*_TPI_^DHAP^ = 0 or *U*_Ribo+TPI_^GAP^ – *U*_TPI_^GAP^ = 0, and the ribosome EEF does not affect the interaction energy of either substrate or (*U*_Ribo+TPI_^DHAP^ – *U*_TPI_^DHAP^) – (*U*_Ribo+TPI_^GAP^ – *U*_TPI_^GAP^) = 0, and the ribosome EEF contributes the same interaction energy to each substrate bound to the active sites. The first case applies for L20 and the second site of L10 + L11, while the second case applies for the first site of L10 + L11 (Table S13). Only the two models involving RP L11 fulfill the energetic and structural conditions required to equate both sides of eq 9 and substantiate the RAMBO effect. Inspection of the structural models for TPI-L11 shows that L11 blocks one of the active sites in the end-to-end binding mode and does not sterically impede substrate binding in the side-to-side model (Figure 6). End-to-end binding of TPI to ribosome protein L11 thus appears to be the best candidate to satisfy eq 9 and validate the RAMBO effect.

**Figure 7:**
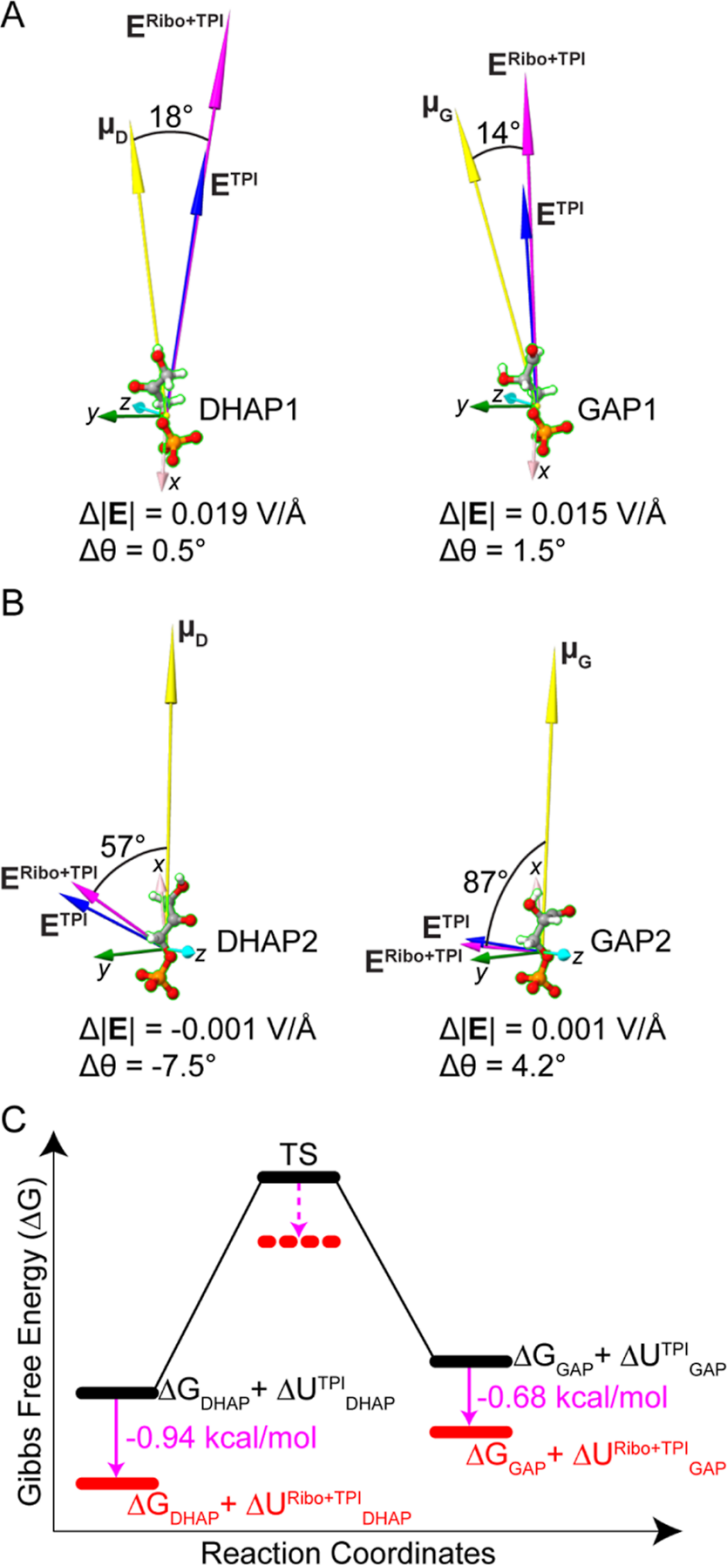
Substrate dipole and ribosome electric field vectors for the TPI-L11 end-to-end complex. (A) Substrate dipole and electric field vectors for TPI active site 1. Electric field vectors for free **E**^**TPI**^ (blue) and ribosome-bound TPI, **E**^**Ribo+TPI**^ (magenta) are shown at the center of mass of DHAP1 and GAP1 relative to the dipole vectors, **μ**_D_ and **μ**_G_ (yellow). (B) Substrate dipole and electric field vectors for TPI active site 2. Electric field vectors for free **E**^**TPI**^ (blue) and ribosome-bound TPI, **E**^**Ribo+TPI**^ (magenta) are shown at the center of mass of DHAP1 and GAP1 relative to the dipole vectors, **μ**_D_ and **μ**_G_ (yellow). Angle shown is between the electric field vector of ribosome-bound TPI and the substrate dipole. Δ|**E**| and Δθ are the changes in the magnitude and orientation of the electric field vectors between free and ribosome-bound TPI. Electric field axes originating from the center of mass of substrates are shown in *x* (pink), *y* (green), and *z* (cyan). Magnitude of the electric field in V/Å was enlarged 500-fold for easy viewing. Images were generated using UCSF ChimeraX.^51^ (C) Energy profile of TPI reaction coordinates showing the average difference in interaction energies (magenta) between free (black) and ribosome-bound TPI (red) for each substrate (Table S13). The resulting stabilization of the TS contributed by the ribosomal electric field, is indicated by a dashed red line and a dashed magenta arrow. Δ*G*_DHAP_ and Δ*G*_GAP_ are the Gibbs free energies of the substrates at the ground state. Δ*U*_DHAP_^TPI^ and Δ*U*_GAP_^TPI^ are substrate molecular dipole-electric field interaction energies when the substrate is bound to free TPI. Δ*U*_DHAP_^Ribo+TPI^ and Δ*U*_GAP_^Ribo+TPI^ are substrate molecular dipole-electric field interaction energies when the substrate is bound to ribosome-bound TPI.

The interaction between substrate dipole moments and electric field vectors for TPI binding in an end-to-end configuration with RP L11 is shown in Figure 7. For ribosome-bound TPI active site 1, the substrate dipole and electric field vectors are 18° apart for DHAP and 14° apart for GAP. This alignment promotes enhanced catalytic activity at this site. For ribosome-bound TPI active site 2, the vectors are 57° apart for DHAP2 and 87° apart for GAP2, which would reduce the effect of the EEF on catalytic activity. Changes in the orientation of **E** between free and ribosome-bound TPI, Δθ, for active site 1 were very small, 0.5 and 1.5°, but had comparatively large changes in magnitude, Δ|**E**|, of 0.015 to 0.02 V/Å, which is predicted to enhance catalytic activity. For substrates bound at active site 2, the changes in orientation were greater, +4.2 to −7.5°, but the changes in magnitude were small, −0.001 to +0.001 V/Å. Because the ribosome EEF affects the magnitude or orientation of the electric field vectors at the active sites of TPI when bound in the end-to-end configuration (Figure 7) but not in the side-to-side configuration (Table S11), side-to-side binding does not contribute to the observed RAMBO effect, although TPI may bind to L11 in this manner (Figure 6C). Therefore, the RAMBO effect depends solely on a single configuration of TPI-L11 in the end-to-end mode.

In the end-to-end model, positively charged K50 and K81 of L11 act in combination with K13 of TPI^22^ to contribute to a substantial increase in the magnitude of the electric field at the first active site upon ribosome binding. A smaller contribution arises from H95, consistent with previous computational analyses on the electrostatic contribution of individual residues.^21^ However, the electric field at the second active site is less affected due to its greater distance from the ribosome surface and the shielding effect of TPI (Figure S11). These configurations reveal that the substrates are asymmetrically stabilized, lowering the energy state of DHAP by 0.97 kcal/mol and that of GAP by 0.68 kcal/mol (Figure 7C). This unequal stabilization is consistent with the kinetic results that the enhanced *k*_cat_ observed for GAP is ∼2-fold greater than for DHAP. The stabilizations substantiate the basis for the RAMBO effect, reinforcing the mechanism of oriented EEF in catalysis.^9,10^

## Discussion and Conclusions

A mechanism for ribosome-mediated amplification of metabolic enzyme activity by coupling the TPI substrate dipole and ribosome EEF vectors was proposed and elucidated. The effect, dubbed RAMBO, increased the activity of the TPI at 1 μM ribosome, boosting the rate of isomerization of DHAP by 22% and by 46% for GAP. We showed that this enhancement can occur when the active TPI-ribosome complex is bound in a particular configuration, end-to-end, to a specific interaction surface on the ribosome, in this case, RP L11. In this configuration, the close alignment of the ribosome EEF and the electric field at the active site of the enzyme, i.e., the reaction center, increased the rate of catalysis.

Previous work from this laboratory has documented protein ribosome interactions and the effect of ribosome binding on the kinetics of pyruvate kinase^16^ and other enzymes,^17^ implying that ribosome-dependent quinary interactions and the associated RAMBO effect may be a general mechanism of regulation. The enhanced activity measured was observed at 1 μM ribosome concentration and is comparable to the enhanced activity observed for other enzymes binding to ribosomes also acquired at 0.5–1.0 μM ribosome concentration.^16,17^ This level of activity represents the experimental maximum and tacitly factors in the fractional occupancy of sites on L11, the fractional occupancy of each binding configuration, of which only end-to-end binding confers enhanced activity, and the fractional occupancy of the active sites. Because the ribosome concentration increases with cell growth^86−88^ and can reach 10 μM in prokaryotes^89^ or 1 μM in eukaryotes,^89^ which would result in more TPI being bound, the RAMBO effect may have an appreciable influence on metabolic fluxes inside the cell.

The comparatively weak interaction between TPI and the ribosome, ∼ 1 μM, contrasts sharply with the much tighter binding of essential translation factors to the ribosome. For example, elongation factor G (EF-G), which has a high intracellular concentration, binds to the ribosome with a dissociation constant of 80 nM;^90^ elongation factor Tu (EF-Tu) has a binding affinity of 250 nM^91^ and the release factors (RF) exhibit even stronger interactions, with a *K*_d_ of 2.5 nM for RF1 and a *K*_d_ of 36 nM for RF3.^92^ Further evidence of TPI-ribosome binding is observed in living cells. Comparing the in-cell NMR spectrum of uniformly ^15^N-labeled TPI (Figure S12, right) with the *in vitro*^1^H–^15^N HSQC spectrum of [U–^15^N] TPI in the presence of 5 μM ribosomes (Figure S12, left), revealed three sets of cross peaks corresponding to ^15^N-labeled amide protons from the side chains of glutamine, asparagine, and tryptophan of TPI that were visible in the *in vitro* spectrum and distinctly broadened in the in-cell spectrum (circled). This observation suggests that TPI is bound to the ribosome inside living cells and that the TPI-L11interaction may not affect ribosome activity, despite the fact that 80–90% of the ribosomes are in a translational state.^93,94^

**Figure 8:**
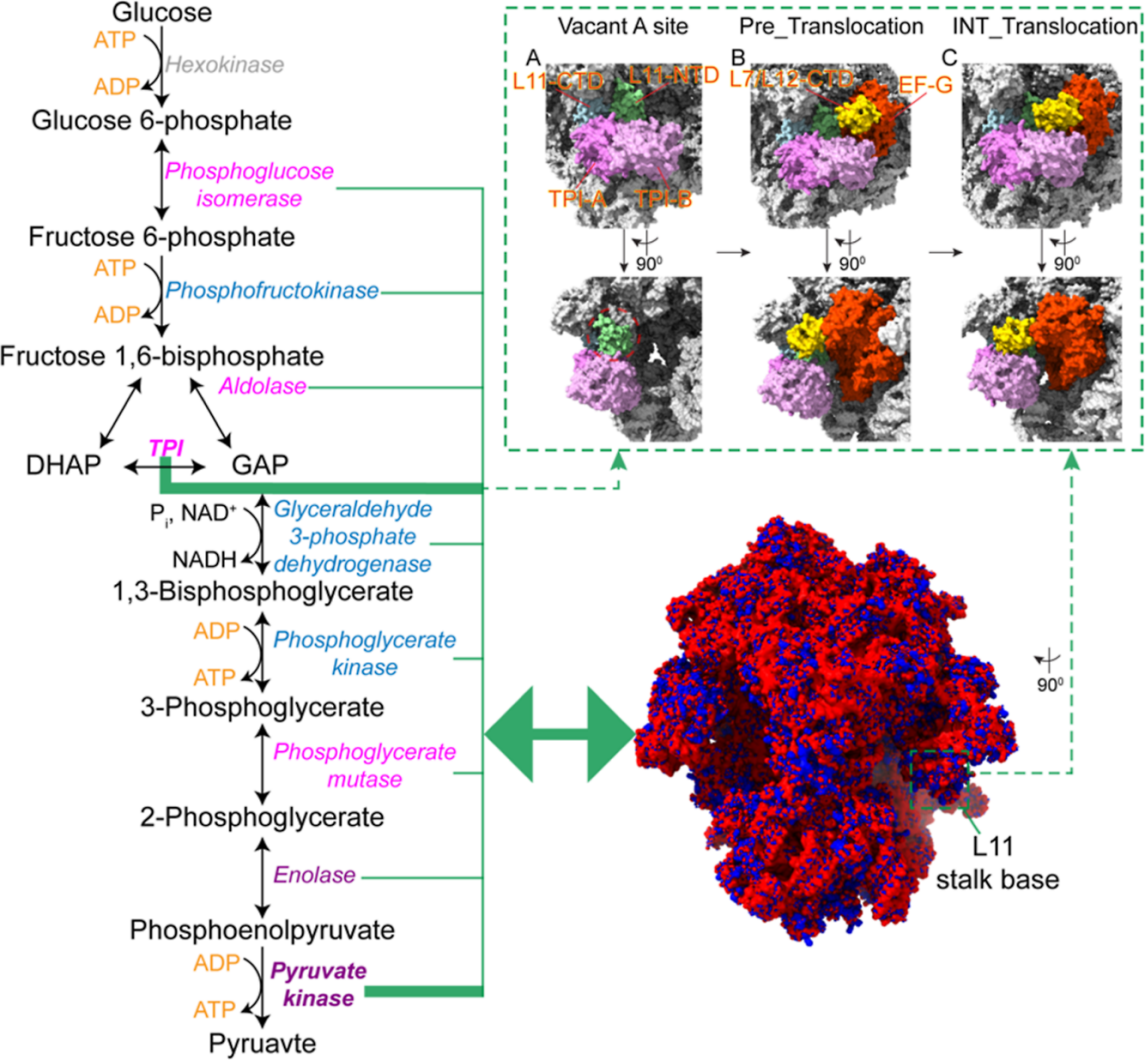
Glycolytic enzyme-ribosome interactions may regulate metabolism. Glycolytic pathway is depicted. All of the enzymes in the pathway except for hexokinase (gray) have been shown to be associated with the ribosome: magenta for mammalian enzymes; light blue for bacterial enzymes; and purple for enzymes from both. Ribosome binding and a consequent effect on enzyme kinetics have been demonstrated for TPI and pyruvate kinase (highlighted in bold). Positive potential (>5 *k*_B_*T/e*, blue) and negative potential (<−5 *k*_B_*T/e*, red) electrostatic iso-surfaces of the intact ribosome are highlighted. The dashed box shows two views of the L11-TPI complex in end-to-end binding mode aligned with L11 from cryo-EM structures of ribosomes undergoing translocation: L11-NTD (light green); L11-CTD (light blue); TPI subunit A (violet); and TPI subunit B (plum). (A) Vacant ribosomal A site (PDB entry 5UYK(81)). (B) CTD of one copy of L7/L12 (gold) bound to EF-G (orange) at the A site prior to translocation (PDB entry 4V7D(102)). (C) Intermediate state of translocation prior to GTP hydrolysis (PDB entry 7N2C(103)). All image were generated by using UCSF ChimeraX 1.3.^51^

L11 is a key RP that contributes to the GTPase-associated center that stimulates the GTPase activity associated with elongation, initiation, and release factors during protein synthesis.^95−97^ The binding sites for elongation factors EF-Tu and EF-G overlap:^98^ The G domains of EF-Tu and EF-G engage the C-terminal domains, CTD, of L7/L12 and they interact with the N-terminal domain, NTD, of L11 to activate the GTPase activity of these factors (Figure 8).^99^ Structural analysis of TPI-L11 end-to-end binding superimposed onto cryo-EM ribosomal structures bound to elongation factors indicated that TPI binding does not interfere with EF-Tu or EF-G binding as seen in time-course ribosome structures during translation (Figure 8).^81,100−103^ In the absence of a ribosome site A occupancy, the binding interface between L11 and TPI in end-to-end mode is close to the CTD of L11 (Figure 8A) and does not sterically occlude pretranslocation binding of EF-G or the CTD of L7/L12 to the NTD of L11 (Figure 8B). Prior to GTP hydrolysis, EF-G undergoes further rearrangement, bringing the CTD of L7/L12 into close contact with two side chain atoms in TPI subunit B but does not block the TPI binding site (Figure 8C). Note that although we observed that TPI does not interact with L7 or L12 *in vitro* (Figure S1) in the absence of EF-G, it remains unclear whether the presence of EF-G could alter this observation. RFs were not considered because there are significantly more ribosome molecules interacting with elongation factors during translation.

To elicit the RAMBO effect, dimeric TPI must be bound to a specific region of RP L11. The chemical driving force for the micromolar affinity binding interaction comes from the high concentration of ribosomes acting as ligands. In the absence of competition, the intracellular concentration of ribosomes is comparable to the estimated binding affinity between TPI and L11, sufficient to occupy >50% of the sites, provided viable TPI is available. The extent to which the overall level of TPI activity is affected is then governed by the fraction of TPI bound to the ribosome. Only dimeric TPI exhibits kinetic activity and the dimerization constant, which is mediated primarily by N-terminal loops Lys13-Asp17 and Gln66-Val79, is estimated to be picomolar or smaller. Under these circumstances, a nascent TPI monomer following translation and folding would be expected to engage in the high affinity dimerization reaction. It is therefore likely that TPI is recruited from a cytosolic pool of TPI dimers. Further investigations will be necessary to understand the interplay between TPI and the ribosome and their implications for the functional state of TPI during and after its synthesis by the ribosome.

Interactions between ribosomes and ATP, ADP^16,17^ and most glycolytic enzymes (Table S14 and Figure 8) have been identified from the mammalian ribosome-interactome,^13^ the *E. coli* glycolytic enzyme interactome,^104^*E. coli* rRNA affinity chromatography,^72^ and *in vivo* cross-linked interaction partners of glycolytic enzyme capture analysis.^105^ The substrates of these enzymes possess dipole moments comparable to those of TPI. These observations suggest the possibility of transient complexes between glycolytic enzymes and the highly charged ribosome surface^106^ forming under the conditions of a crowded cytosol, enhancing the response to local energy demand.^107^ Cytosolic arrays of ribosomes in prokaryotes could act as an organizing center analogous to that in eukaryotic supramolecular assemblies that act to compartmentalize metabolic pathways. The putative glycolytic metabolon,^108−110^ in conjunction with ribosome-dependent modulation of glycolysis, adds an additional layer of regulation to the metabolic flux in living organisms.

## Supporting Information Available

TPI does not interact with RPs L7 or L12; calculation of substrate dipole moment (**μ**) and electric field vectors (***E***); Matlab code for electric field calculations; [*U–*^15^N]-TPI purification; ribosome binding to free [*U–*^15^N]-TPI uniformly broadens cross peak intensities; binding of substrate-bound TPI to ribosomes; TPI activity assay; representative high-energy collision MS spectrum; substrate-bound TPI models; molecular surface mapping of TPI-RP quinary interactions; TPI interacts with ribosome inside living *E. coli* cells; active site electric fields for TPI bound end-to-end to L11; possible intermolecular cross-links; possible RP binding partners for TPI; filtered distance restraints and predicted interacting residues for TPI-ribosome docking; TPI-ribosome binding conformations; orthogonal axes originating at the C2 of substrates in Cartesian coordinates; orthogonal axes originating at the C2 atom of substrates in Q-Chem coordinates; database inputs for pLink; substrate dipole moments; atomic coordinates of TPI monomers within the homodimer; electric fields at the center of substrate mass for free and ribosome-bound TPI; magnitude and orientation of substrate dipole and electric field vectors; substrate dipole-electric field interaction energies for free and ribosome-bound TPI; differences in dipole-electric field interaction energies between free and ribosome-bound TPI for each site; and ribosome-binding glycolytic enzymes related to Figure 8 (PDF)

## Accession Codes

TPI, UniProt entry P00940, PDB entries 8TIM and 1TPH, and BMRB entries 15064 and 15065; 70S ribosome with EF-Tu and aminoacyl-tRNA in the preaccommodation state, PDB entry 5UYK. 70S Ribosome with EF-G in the pretranslocation state, PDB entry 4V7D; 70S ribosome with EF-G in the intermediate state of translocation, PDB entry 7N2C.
