# Supplementary material for: Ribosome External Electric Field Regulates Metabolic Enzyme Activity: The RAMBO Effect
Source: J Phys Chem B. 2024 Jul 16;128(29):7002–21. doi: 10.1021/acs.jpcb.4c00628 (PMC11284791; doi:10.1021/acs.jpcb.4c00628)
Supplement: Supplementary file 1 — jp4c00628_si_001.pdf [file jp4c00628_si_001.pdf]

Supplementary Material

Ribosome External Electric Field Regulates Metabolic Enzyme Activity: The RAMBO Effect.

*Jianchao Yu[1], Lisa M. Ramirez[1†], Qishan Lin[2], David S. Burz[1], and Alexander Shekhtman[1]**

[1]Department of Chemistry, University at Albany, State University of New York, Albany, New York, 12222, United States

[2]RNA Epitranscriptomics & Proteomics Resource, University at Albany, State University of New York, Albany, New York, 12222, United States

[†]Present address: Deutsches Zentrum für Neurodegenerative Erkrankungen (DZNE), von-Siebold-Str. 3a, 37075, Göttingen, Germany.

*Corresponding author, email: ashekhtman@albany.edu

KEYWORDS: enzyme kinetics, triosephosphate isomerase, ribosome, molecular docking, chemical crosslinking, dipole-field interaction energy, electric field, enzyme catalysis, moonlighting enzymes, macromolecular crowding, quinary structure.

Supplementary Information

Figure S1. TPI does not interact with ribosomal proteins L7 or L12.

Figure S*2*. Calculation of substrate dipole moment (**μ**) and electric field vectors (**E**).

Figure S3. Matlab code for electric field calculations.

Figure S4. [*U*-^{15}N]-TPI purification.

Figure S5. Ribosome binding to free [*U*-^{15}N]-TPI uniformly broadens cross peak intensities.

Figure S6. Binding of substrate-bound TPI to ribosomes.

Figure S7. TPI activity assay.

Figure S8. Representative high-energy collision MS spectrum.

Figure S9. Substrate-bound TPI models.

Figure S10. Molecular surface mapping of TPI-RP quinary interactions.

Figure S11. Active site electric fields for TPI bound end-to-end to L11.

Figure S12. TPI interacts with ribosome inside living *E.coli* cells.

Table S1. Possible intermolecular crosslinks

Table S2. Possible RP binding partners for TPI.

Table S3. Filtered distance restraints and predicted interacting residues for TPI-ribosome docking.

Table S4. TPI-ribosome binding conformations.

Table S5. Orthogonal axes originating at the C2 of substrates in Cartesian coordinates.

Table S6. Orthogonal axes originating at the C2 atom of substrates in Q-Chem coordinates.

Table S7. Database inputs for pLink.

Table S8. Substrate dipole moments.

Table S9. Atomic coordinates of TPI monomers within the homodimer.

Table S10. Electric fields at the center of substrate mass for free and ribosome-bound TPI.

Table S11. Magnitude and orientation of substrate dipole and electric field vectors.

Table S12. Substrate dipole-electric field interaction energies for free and ribosome-bound TPI.

Table S13. Differences in dipole-electric field interaction energies between free and ribosome-bound TPI for each site.

Table S14. Ribosome-binding Glycolytic Enzymes (related to Figure 8).

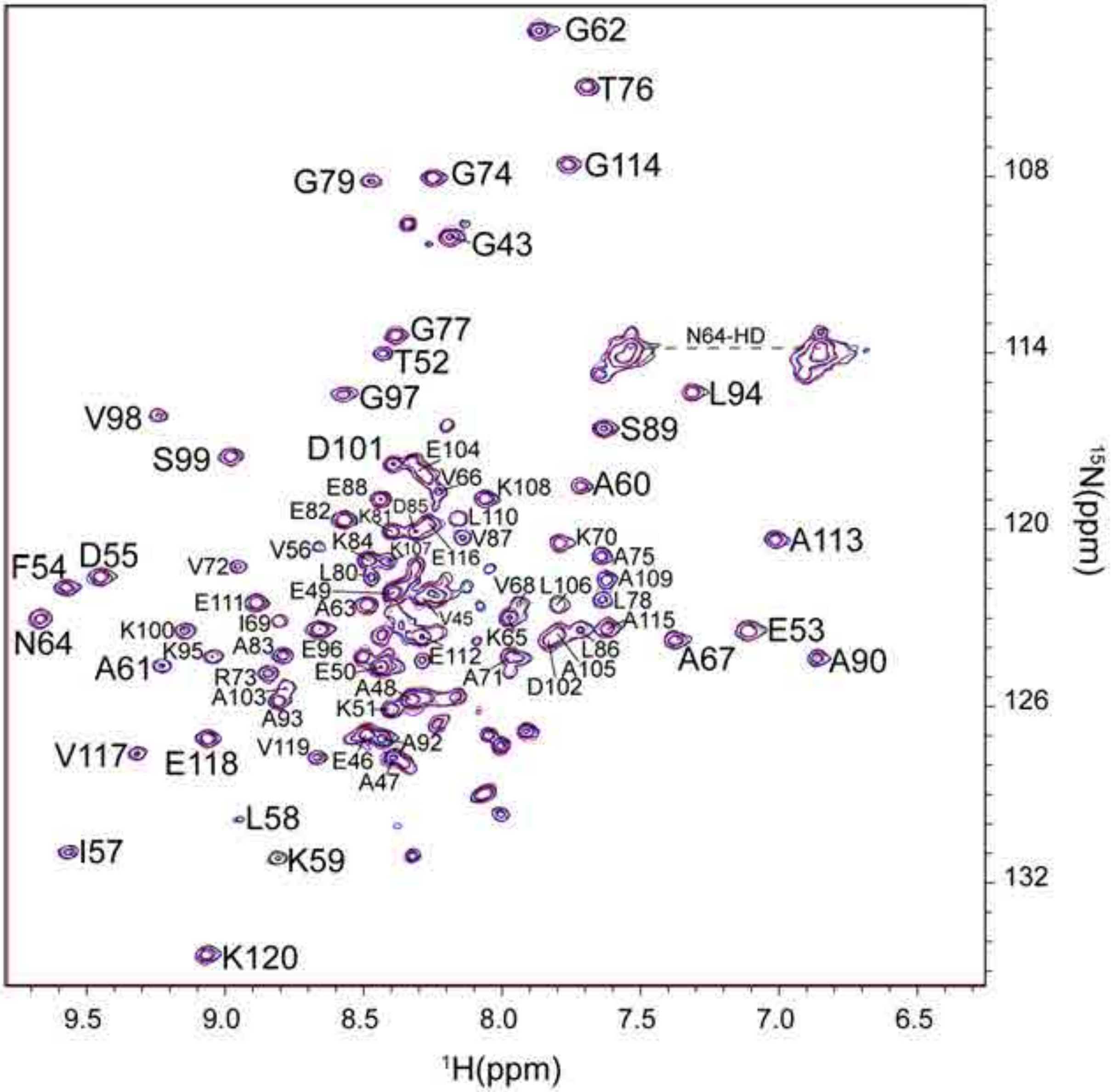

Figure S1. TPI does not interact with ribosomal proteins L7 or L12.
Overlay of ^{1}H-^{15}N HSQC spectra of 10 µM [U-^{15}N]-ribosomes titrated with 0 µM (black), 50 µM (blue) and 150 µM (red) His-tagged TPI. The well-resolved cross peaks are from the highly mobile L7/L12 RPs. Assignments were made based on isolated ribosomal L7/L12 protein[1] (BMRB entry 4429).

(1) Bocharov, Eduard; Gudkov, Anatolij; Arseniev, Alexandr. (1996) Topology of the secondary structure elements of ribosomal protein L7/L12 from *E.coli* in solution. *FEBS Lett.* 379, 291-294.

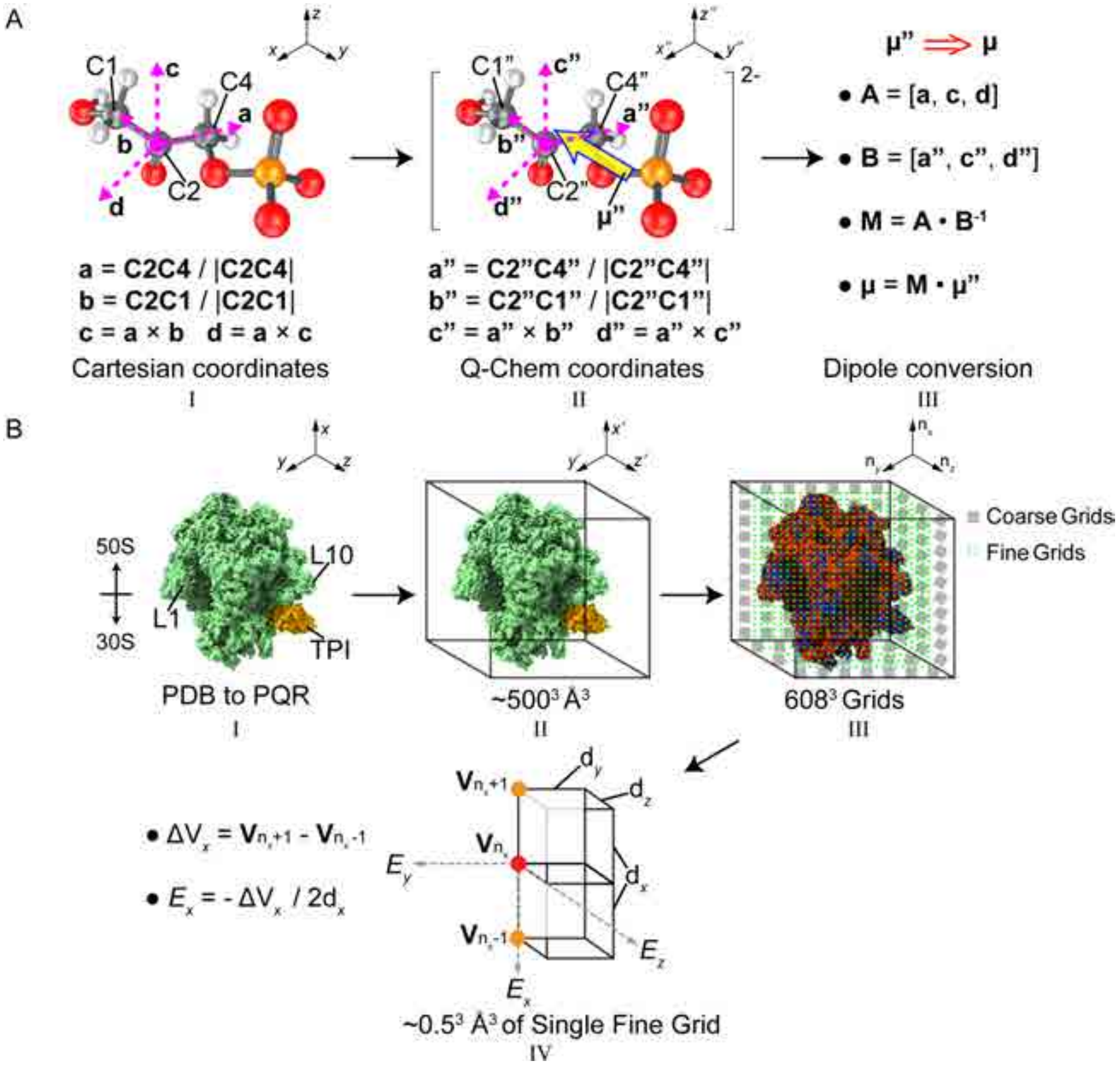

Figure S2. Calculation of substrate dipole moment (μ) and electric field vectors (E).

A) Workflow for calculating substrate dipole moments, **μ**. (left) DHAP is represented by a ball-and-stick model (H: white, C: grey, O: red, P: orange). **a** and **b** (magenta arrows) are the unit column vectors C2C4 and C2C1, respectively. **c** and **d** (magenta arrows) are orthogonal axes originating at C2 that establish the orientation of DHAP in Cartesian coordinates in matrix **A**. (center) The unit vectors and carbon atoms are converted to Q-chem coordinates to define matrix **B**, which is used to record the orientation of DHAP after calculating the molecular dipole (yellow arrow). (right) Rotation matrix **M** is used to convert the dipole vector into Cartesian coordinates (**μ"** → **μ**). Note that the dipole vector arrow goes from negative to positive. B) Workflow for calculating the electric field, **E**. (left) PDB files generated for substrate-bound TPI-ribosome models are converted from Cartesian coordinates (x, y, z) to PQR format at pH 7 to assign atomic radii and charges. Ribosomal proteins L1 and L10, and the 50S and 30S subunits are indicated for

orientation of the ribosome (light green) bound TPI dimer (orange). (center) The PQR model is oriented within an ~500^3 Å^3 volume and the origin of the Cartesian coordinates is shifted to the origin of the Adaptive Poisson-Boltzmann solver, APBS, coordinate system (x', y' z'). (right) The electric field, **V**, is calculated by solving the Poisson-Boltzmann equation in two steps: The initial calculation utilizes 608^3 grids within a 500^3 Å^3 potential grid box (n_x , n_y , n_z). Red and blue are positive and negative surface potentials. The final calculation also utilizes 608^3 grid points but in a reduced volume, equal to that of the TPI-ribosome complex, to achieve a resolution of ~0.5 Å. (bottom) The components of the electric field ($\boldsymbol{E_x}$, $\boldsymbol{E_y}$, $\boldsymbol{E_z}$) are resolved by determining the electric field at every point in the potential grid box (*e.g.* red solid circle at $\boldsymbol{E_x}$); d_x , d_y and d_z are the fine grid spacings measured in Å. For the x-component, the potential difference between $\boldsymbol{V}$ (n_x+1, n_y , n_z) and $\boldsymbol{V}$ (n_x-1, n_y , n_z), $\Delta \boldsymbol{V_x}$, is divided by twice the grid spacing along x, d_x , to yield a value for $\boldsymbol{E_x}$.

```
% Matrix Restructuring and Electric Field calculations
% Use matrix A stored in memory from dx file in 75288843*3, which has total 609*609*609 points
% Result is potential V(609,609,609)
% Electric field is stored in E(3, 608, 608, 608), where 1, 2, and 3 in E(3, n1, n2, n3) corresponds to
% x, y, and z components of the electric field
% Comments: dx file is built in the following way:
%       object 1 class gridpositions counts nx ny nz
%       origin xmin ymin zmin
%       delta dx 0.0 0.0
%       delta 0.0 dy 0.0
%       delta 0.0 0.0 dz
%       object 2 class gridconnections counts nx ny nz
%       object 3 class array type double rank 0 times n
%       u(0,0,0) u(0,0,1) u(0,0,2)
%       ...
%       u(0,0,nz-3) u(0,0,nz-2) u(0,0,nz-1)
%       u(0,1,0) u(0,1,1) u(0,1,2)
%       ...
%       u(0,1,nz-3) u(0,1,nz-2) u(0,1,nz-1)

V = zeros(609,609,609);
E = zeros(3, 608, 608, 608);
dx=0.4801105;
dy=0.5627240;
dz=0.5165428;
l1=0;
m1=0;
ii=0;
for k1 = 1:609
for j1 = 1:609
for i1 = 1:609
ii = (k1-1)*609*609 + (j1-1)*609 + i1;
l = int32(ii/3-0.51);
l1=l+1;
m1 = int32(ii -3*l);
k2= int32(k1);
j2= int32(j1);
i2= int32(i1);
V(k2,j2,i2) = A(l1,m1);
end
end
end
for n1=2:607
   for n2=2:607
      for n3=2:607
            E(1, n1, n2, n3) = - (V(n1+1, n2, n3)-V(n1-1, n2, n3))/(2*dx);
            E(2, n1, n2, n3) = - (V(n1, n2+1, n3)-V(n1, n2-1, n3))/(2*dy);
            E(3, n1, n2, n3) = - (V(n1, n2, n3+1)-V(n1, n2, n3-1))/(2*dz);
      end
   end
end
```

Figure S3. Matlab code for electric field calculations.

Input the APBS calculated potential file without headlines and footnotes, A = load (Potential_file_name.dx). Run the Matlab code (above) for calculations by code_file_name.m. Type E(i, n1, n2, n3) with the desired location number inside the grid box to obtain the *x*, *y*, *z* components of the electric field.

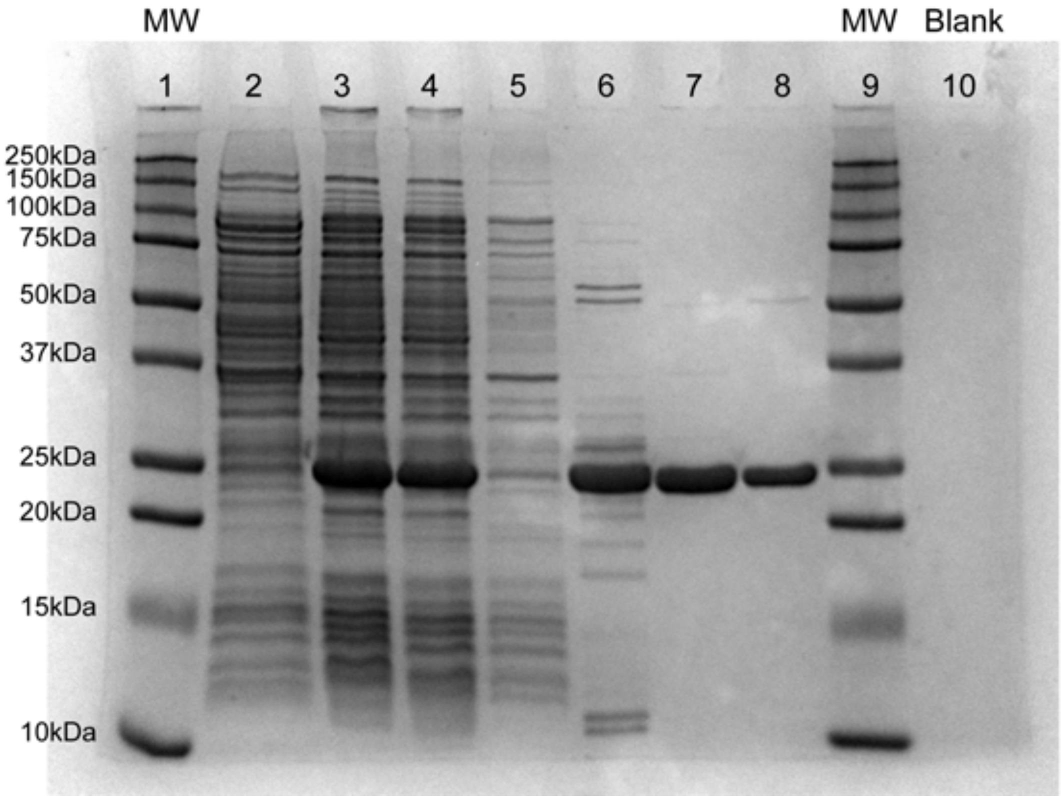

Figure S4. [*U*-^{15}N]-TPI purification.
Lanes 1 and 9: molecular weight makers (MW). Lane 2: whole cell lysis before IPTG induction. Lane 3: whole cell lysis 18 h post-induction. Lane 4: whole cell lysis supernatant. Lane 5: HiTrap DEAE FF column flow through. Lane 6: 1st HPLC elution of ~10 µM [*U*-^{15}N]-TPI. Lane 7: 2nd HPLC elution of ~10 µM [*U*-^{15}N]-TPI. Lane 8: 2nd HPLC elution of ~10 µM nonlabelled TPI. Lane 10: Laemmli sample buffer blank.

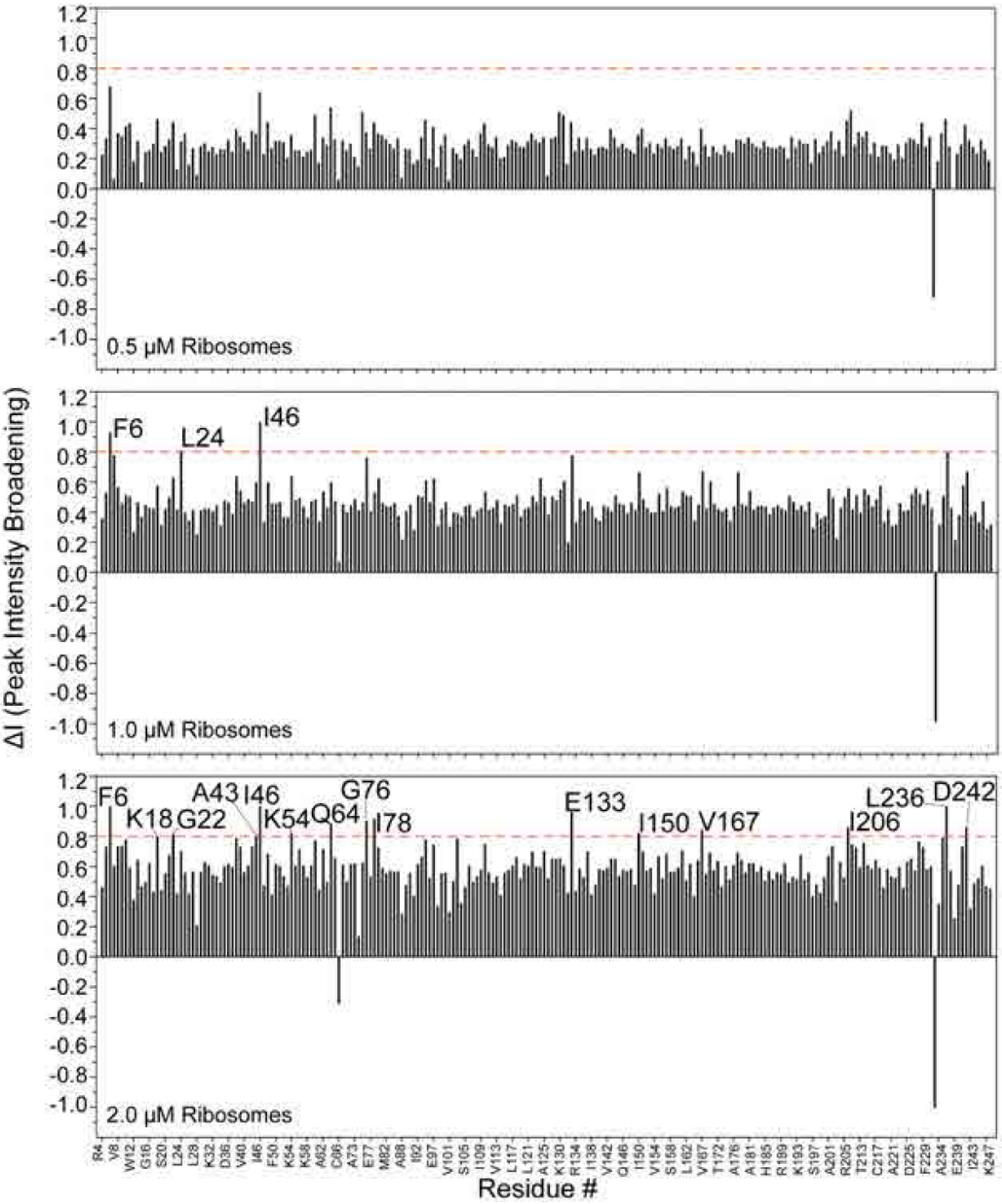

Figure S5. Ribosome binding to free [*U*-^{15}N]-TPI uniformly broadens cross peak intensities.
A) Changes in peak intensities, calculated by using $\Delta I = ((I_{free}/I_{ref}) - (I_{bound}/I_{ref}))/(I_{free}/I_{ref})$, where I_{free}/I_{ref} is the normalized intensity of each cross peak in the free ^{1}H-^{15}N HSQC TPI spectrum in the absence of ribosomes and I_{bound}/I_{ref} is the normalized intensity of each cross peak in the ^{1}H-^{15}N HSQC TPI spectrum in the presence of ribosomes. I_{ref} is the peak intensity of a glutamine side chain amide at 6.92 and 112.9 ppm in the proton and nitrogen dimensions respectively and does not shift upon ribosome titration. Residues that showed extreme broadening (ΔI >80%) are highlighted.

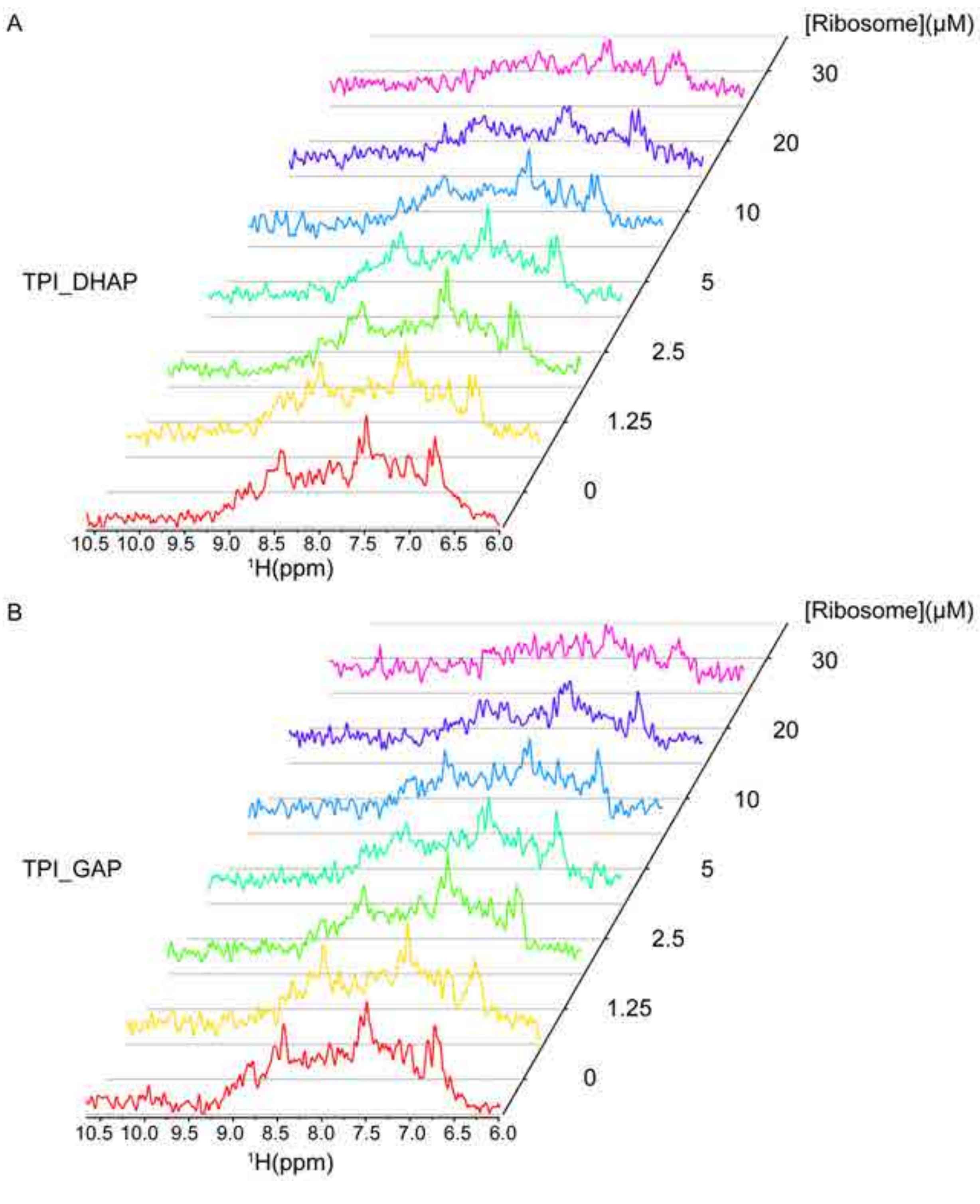

Figure S6. Binding of substrate-bound TPI to ribosomes.
30° stacked view of ^{1}H-^{15}N HSQC amide proton envelope spectra of A) DHAP bound and B) GAP bound TPI with increasing amounts of ribosomes. TPI was at 2.5 µM, DHAP and GAP were at 5 mM and 1 mM, respectively. Spectra were processed and exported by using MestReNova 14.0.0.

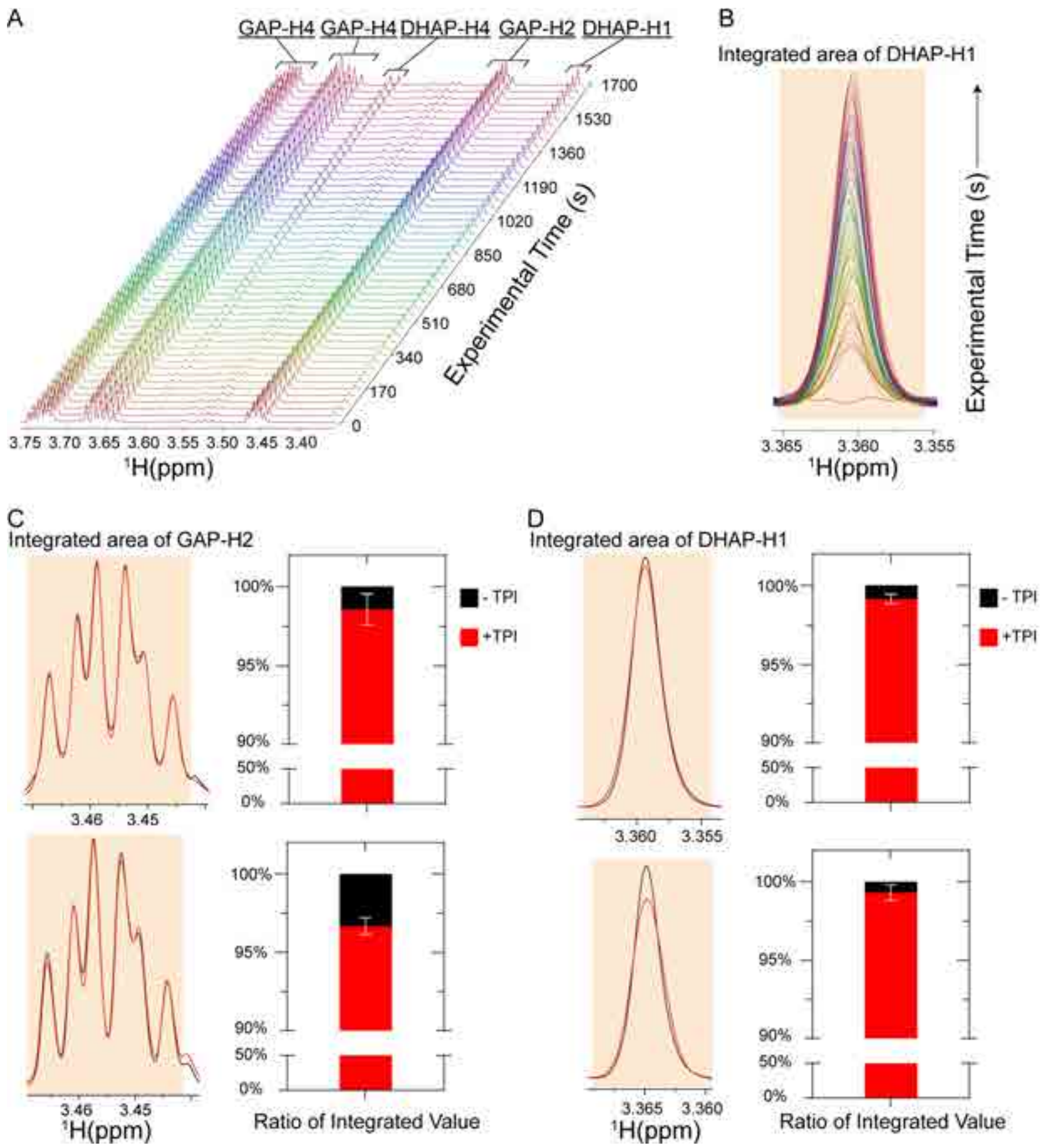

Figure S7. TPI activity assay.

A) Typical pseudo-2D NMR spectra of TPI activity displayed in 30° stacked view. 1D proton spectra were collected at 34 s intervals. Peaks correspond to H4 of GAP (3.74 ppm, 3.66 ppm), H2 of GAP (3.46 ppm), H4 of DHAP (3.60 ppm), and H1 of DHAP (3.36 ppm). B) Spectra were analyzed in a superposition mode by integrating the H1 proton peak of DHAP and converting the values to concentrations. C) Superimposed H2 proton peaks of 1 mM GAP integrated over the same range before (black) and after (red) the addition of TPI without (top) and with (bottom) 1 μM ribosomes. The ratios of integrated values show a loss of <10% GAP during the reaction dead time under both conditions. D) Superimposed H1 proton peaks of 1 mM DHAP integrated over the same range before (black) and after (red) the addition of TPI without (top) and with (bottom) 1 μM ribosomes. The ratios of integrated values show a loss of <10% DHAP during the reaction dead time under both conditions.

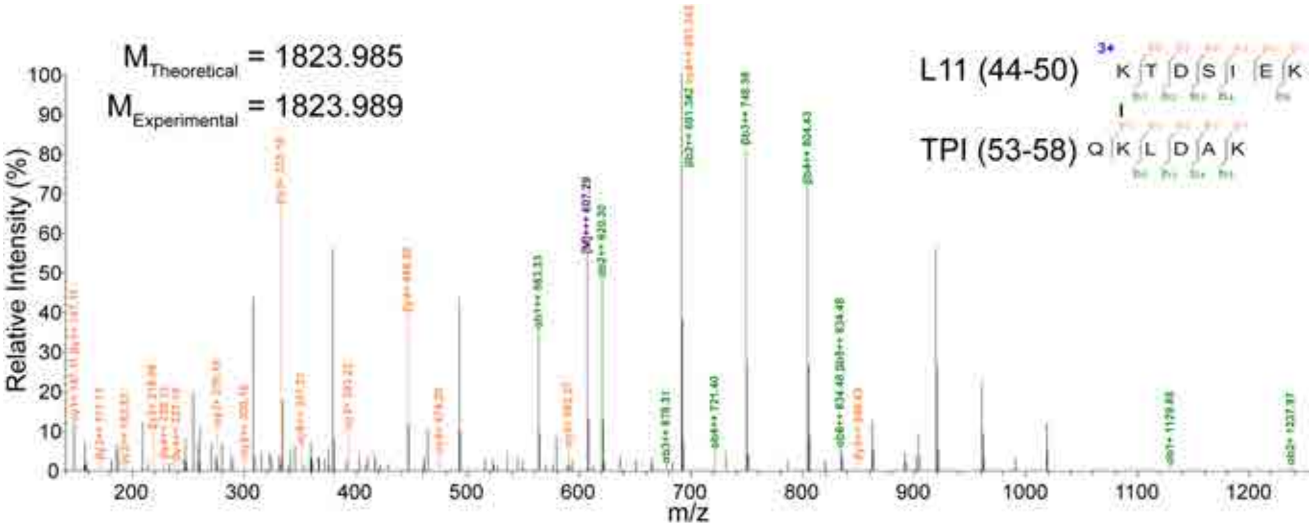

Figure S8. **Representative high-energy collision MS spectrum.**

The cross-linked peptide between ribosomal protein L11 and TPI is seen at m/z 607.29 (purple). The experimental mass ($M_{Experimental}$) of this triple charged precursor ion, 1823.989, matched the theoretical mass ($M_{Theoretical}$) of 1823.985. The peptide sequences with cross-linked lysines are shown (top right). The b fragmentation ions (green) that extend from the amino terminus and y fragmentation ions (orange) that extend from the carboxyl terminus are labeled. The image was prepared using pLabel (http://pfind.org/software/pLabel) with 5 Da tolerance.

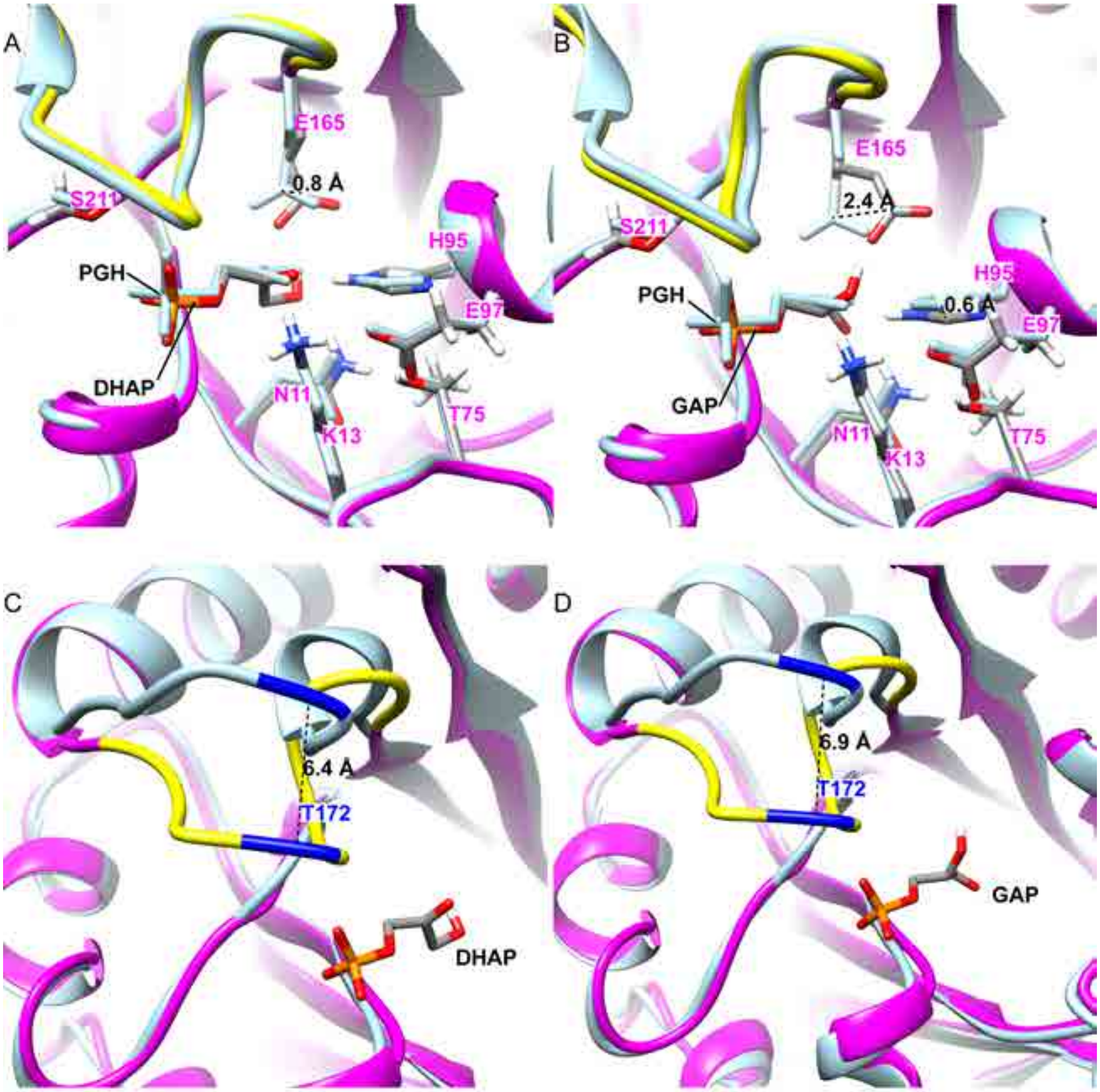

Figure S9. Substrate-bound TPI models.

Ribbon diagrams of (A) DHAP- and (B) GAP-bound TPI after energy minimization are highlighted in magenta with dynamic loop 6 in yellow; PGH-bound TPI (PDB entry 1TPH) prior to energy minimization is in light blue. Active site residues N11, K13, H95, E97, E165 and S211 from one monomer and T75 from the other monomer, are shown with color-coded side chains: hydrogen (white), carbon (gray), nitrogen (blue), oxygen (red) and phosphorus (orange). Ribbon diagrams of TPI bound with DHAP (C) and GAP (D) at the first active site after energy minimization are highlighted in magenta and free TPI (PDB entry 8TIM) is in light blue. The displacement of C_α of T172 (dark blue) at the tip of loop 6 (yellow) between bound (active) and unbound (inactive) TPI is indicated.

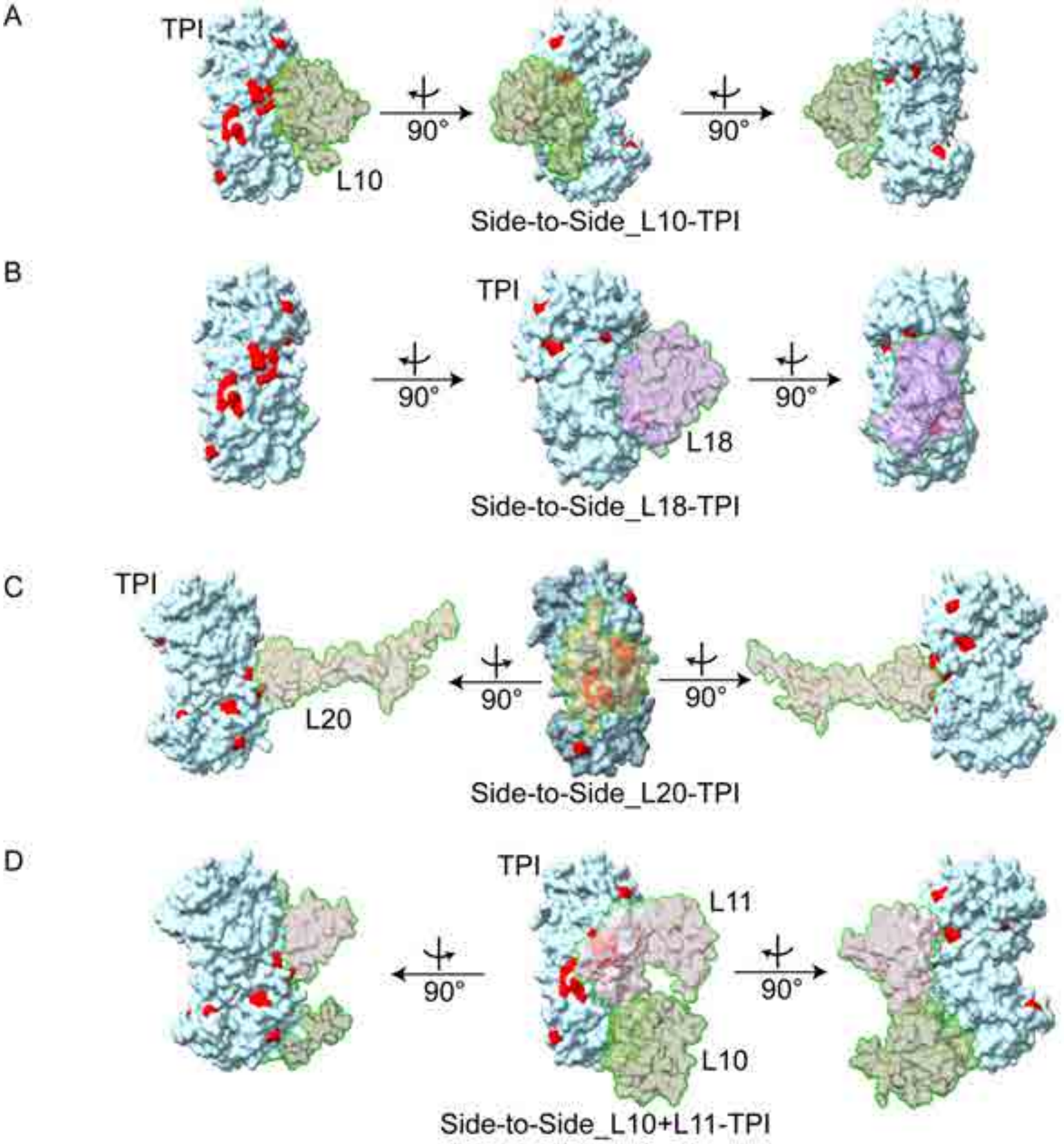

Figure S10. Molecular surface mapping of TPI-RP quinary interactions.

Red patches indicate binding interface residues exhibiting broadened [*U*-^{15}N]-TPI cross peaks in ^{1}H-^{15}N HSQC spectra. (A) Residues G22 and L236 from subunit A were involved in the binding interface between L10 and TPI under 'Side-to-Side' mode. (B) Residues E133 from both subunits were involved in the binding interface between L18 and TPI under 'Side-to-Side' mode. (C) Residues K18, G22, K54 from both subunits were involved in the binding interface between L20 and TPI under 'Side-to-Side' mode. (D) Residues K18, G22, K54, and L236 from subunit A were involved in the binding interface between L10 + L11, and TPI under 'Side-to-Side' mode. TPI was modelled using PDB entry 8TIM.

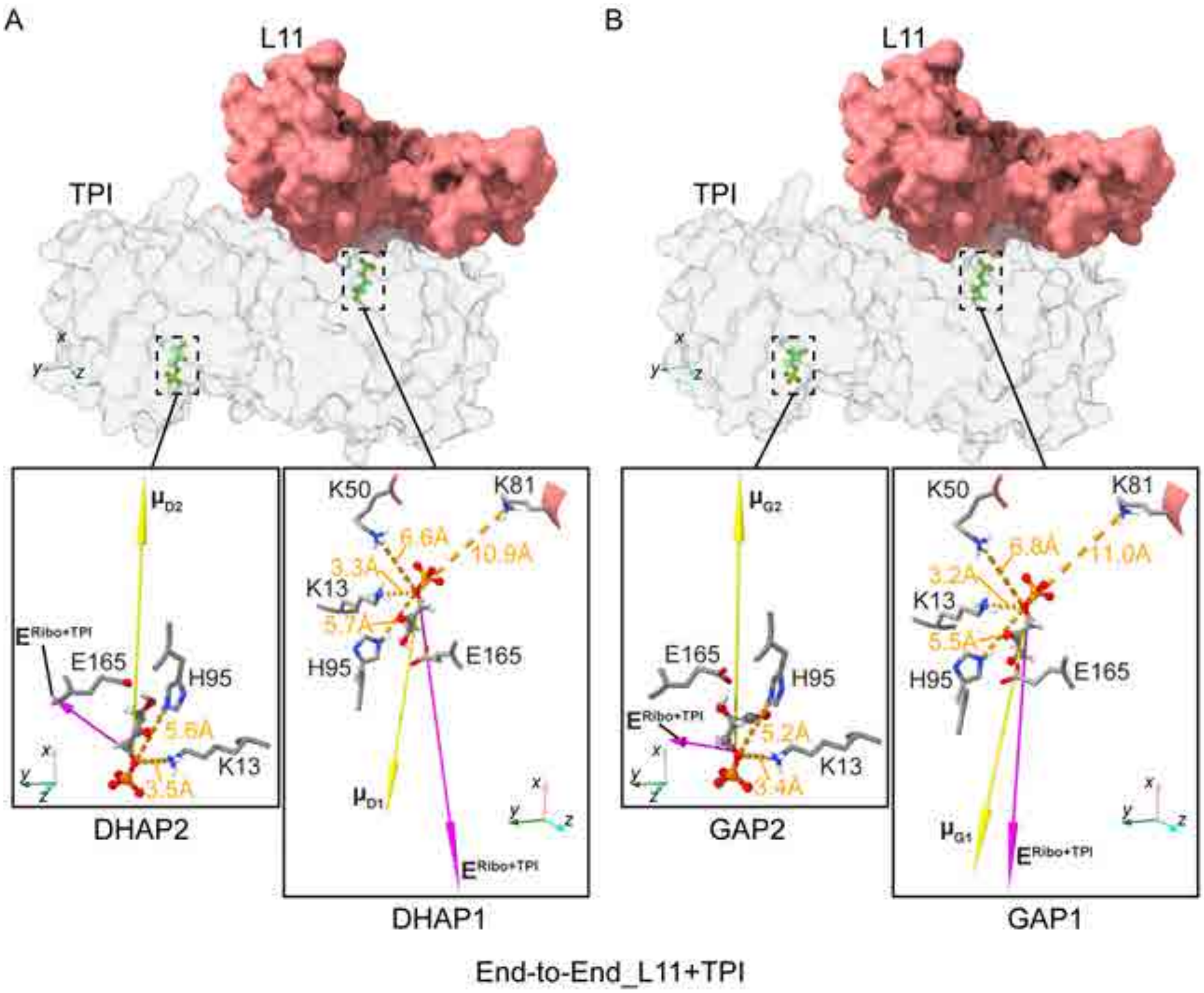

Figure S11. Active site electric fields for TPI bound end-to-end to L11.
Space filling models of TPI and RP L11 for DHAP (A) and GAP (B). (Insets) The electric field ($\mathbf{E}^{Ribo+TPI}$) at the center of mass of each substrate results primarily from K13 of TPI at active site 2 and from K50 and K81 of L11 at site 1. TPI active site residues H95 and E165 also contribute to the electric field. The distances between the terminal nitrogen, NZ, of each Lys or NE2 of His95 and O1P of DHAP (left) and GAP (right) are indicated by dashed lines.

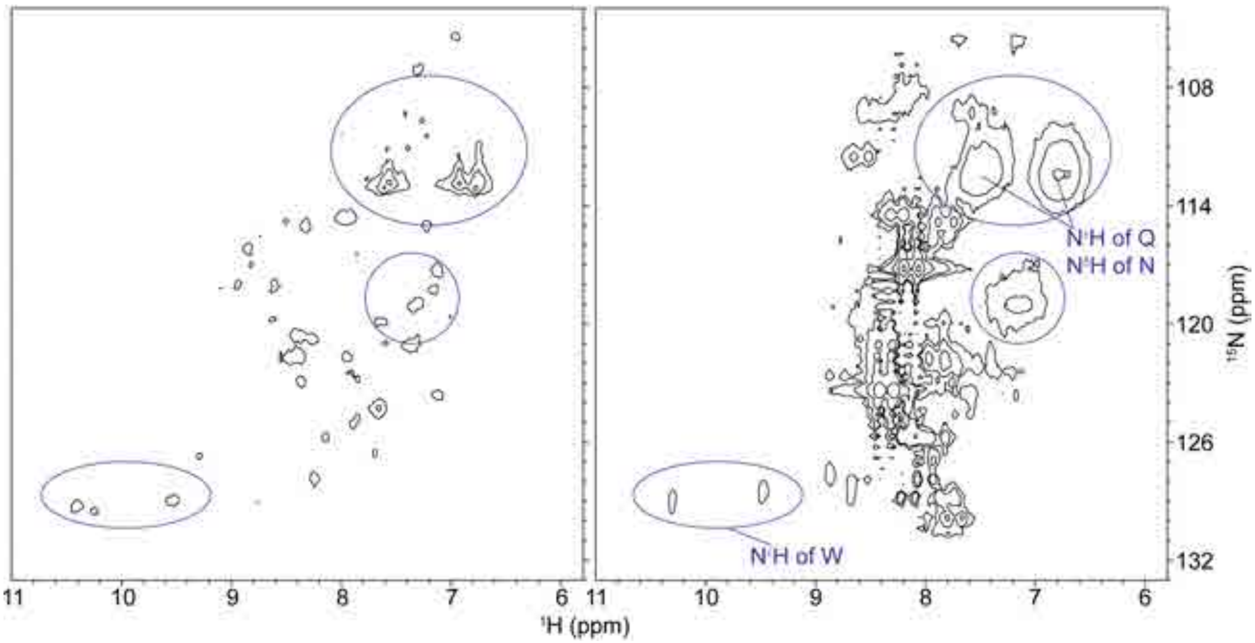

Figure S12. TPI interacts with ribosome inside living *E.coli* cells. Left, ^{1}H-^{15}N HSQC spectrum of 50 μM [U-^{15}N]-TPI in the presence of 5 μM intact ribosomes. Right, in-cell ^{1}H-^{15}N CRINEPT-HMQC-TROSY[1] spectrum of [U-^{15}N]-TPI. The peaks detected in the central region of the in-cell spectrum are mostly from the [U-^{15}N]-TPI and [U-^{15}N]-amino acids inside living cells. Peaks from the amide protons (circled) of glutamine, Q, asparagine, N, side chains and the imino proton of tryptophan, W, indoles in the *in vitro* spectrum are distinctly broadened in the in-cell spectrum (circled)..

(1) Riek, R.; Wider, G.; P. Vushin, K.; Wuthrich, K. Polarization transfer by cross-correlated relaxation in solution NMR with very large molecules. *Proc. Natl. Acad. Sci.* **1999**, *96*, 4918-4923.

Table S1. Possible intermolecular crosslinks.

RPs	Crosslinked Sequences: TPI-RP [a]	Crosslinked Residues	$M_{theoretical}$	$M_{experimental}$	Error (ppm)	TPI Residue Solvent Accessibility [b,c]	RPs Residue Solvent Accessibility [b]
**L9** [d]	HALAEGLGVIACIGEKLD-KAGDEGK	TPI(K130)-L9(K83)	2814.444	2814.452	2.9	52%	62%
**L10** [e]	WAIGTGKT-ECLKDAFVGPTLIAYSM	TPI(K174)-L10(K73)	3065.510	3065.507	-0.9	42%	66%
	NAKHLEHHHHH-VSEVAKGA	TPI(K247)-L10(K20)	2458.222	2458.227	2.0	57%	76%
	GNCKELASQH-VAKGALSAVVADSR	TPI(K218)-L10(K20)	2788.414	2788.422	2.8	82%	76%
**L11**	LDAKIG-AKTDSIEK [f]	TPI(K58)-L11(K44)	1865.995	1865.993	-1.0	63%	47%
	IGTGKT-SIEKG	TPI(K174)-L11(K50)	1410.757	1410.755	-1.6	42%	58%
	TLNGAKL-TDSIEKG [f]	TPI(K32)-L11(K50)	1766.927	1766.924	-1.4	98%	58%
	AIGTGKTATPQQAQEVHEK-IMEFCKAFNA [g]	TPI(K174)-L11(K39)	3484.697	3484.705	2.3	42%	81%
	ELASQHDVDGFLVGGASLKPEFVDIIN-IMEFCKAFNAKT [g]	TPI(K237)-L11(K44)	4590.267	4590.274	1.6	66%	47%
	GASLKPEFVDI-NIMEFCKAFNA [g]	TPI(K237)-L11(K39)	2780.341	2780.347	2.1	66%	81%
	GEKLDER-GIKSGSGKP	TPI(K130)-L11(K86)	1978.034	1978.029	-2.2	52%	89%
	QKLDAK-KTDSIEK	TPI(K54)-L11(K44)	1823.985	1823.989	2.2	55%	47%
	PEFVDIINAKHLEHHHHH-IEKGL	TPI(K247)-L11(K50)	3070.574	3070.576	0.5	57%	58%
	SLGELIHTLNGAKLS-GKISRA	TPI(K32)-L11(K99)	2485.387	2485.390	1.2	98%	71%
**L15**	AIGTGKTATPQ-AAIEAAGGKI	TPI(K174)-L15(K141)	2246.212	2246.209	-1.7	42%	79%
**L18**	IINAKHLEHHHHH-TGNKD [f]	TPI(K247)-L18(K68)	2458.222	2458.227	2.0	57%	65%

Continued page of Table S1

	GVIACIGEKL-GIKDV	TPI(K130)-L18(K88)	1892.030	1892.027	-2.1	52%	78%
	HALAEGLGVIACIGEKLD-AVGKAVAERA	TPI(K130)-L18(K76)	3138.671	3138.670	-0.5	52%	72%
	IIYGGSVTGGNCKE-AVGKAVAER	TPI(K218)-L18(K76)	2656.350	2656.351	0.6	82%	72%
**L20**	HTLNGAKL-VEKAKA	TPI(K32)-L20(K111)	1800.011	1800.008	-1.6	98%	53%
**L22** [h]	VTGGNCKE-ADIDDLKVT	TPI(K218)-L22(K70)	2155.032	2155.032	-0.05	82%	42%
**S6** [i]	TGGNCKELAS-HKAHYVLMN	TPI(K218)-S6(K56)	2409.142	2409.137	-2.4	82%	63%
**S8** [d]	DNVKDWSK-VTMPSSKLK	TPI(K155)-S8(K30)	2283.179	2283.175	-1.4	100%	100%
	ELASQHDVDGFLVGGASLKP-KDELPK	TPI(K237)-S8(K88)	3070.583	3070.580	-1.1	66%	70%
	VAHALAEGLGVIACIGEKLDEREA-GLGIAVVSTSKGV	TPI(K130)-S8(K107)	3953.115	3953.106	-2.4	52%	71%
	KVVFEQTK-KGVMTDRAARQA	TPI(K141)-S8(K107)	2583.381	2583.378	-1.1	63%	71%
**S11**	AKIGVAAQNCYKVPK-AVKEYGIKNLEVM	TPI(K68)-S11(K74)	3457.832	3457.831	-0.3	40%	91%
	VAHALAEGLGVIACIGEKLDEREAGITEKVVFE-GIKNLEVM[j]	TPI(K130)-S11(K79)	4687.446	4687.443	-0.6	52%	90%
	VAHALAEGLGVIACIGEKLDER-VKEYGIK[e]	TPI(K130)-S11(K74)	3458.845	3458.837	-2.4.	52%	91%

[a] Green-labeled M residues were modified by oxidation and C residues were modified by carbamidomethylation. Bold red-labeled K residues indicate crosslinked lysines.
[b] Residues are considered solvent exposed if the ratio value is over 40% and solvent inaccessible if the ratio is less than 20%.
[c] Solvent accessibility of residues from each monomer of TPI was calculated together and input as average values.
[d] L9 and S8 were identified using modified settings: nonspecific in enzyme channel; no fixed or variable modifications applied.
[e] L10 and one crosslinked peptide related to S11 were identified by pLink2.0 using modified settings: nonspecific in enzyme channel, and both carbamidomethyl [C] and oxidation [M] as a fixed modification.
[f] Two crosslinked peptides of L11 and one belonging to L18 were identified using modified settings: nonspecific in enzyme channel and carbamidomethyl [C] as a fixed modification.

[g] Three additional crosslinked peptides of L11 were identified using modified settings: nonspecific in enzyme channel and oxidation [M] as variable modification.

[h] L22 was identified by searching under modified settings: nonspecific in enzyme channel and carbamidomethyl [C] as variable modification.

[i] S6 was identified using modified settings: nonspecific in enzyme channel, and both carbamidomethyl [C] and oxidation [M] as a variable modification.

[j] One additional crosslinked peptide of S11 was identified using modified settings: nonspecific in enzyme channel and oxidation [M] as fixed modification.

Table S2. Possible RP binding partners for TPI.

RPs	# Crosslinks		
	pLink [a]	DisVis [b]	
		E-E	S-S
L9	1	1	2
L10	6	3	4
L11	17	10	12
L15	3	1	2
L18	4	4	6
L20	2	1	2
L22	2	1	[c]
S6[d]	6	1	[c]
S8	6	4	7
S11	5	3	6
L10+L11	23	12	11
L15+L18	7	5	5
L9+S6	7	2	2
S6+S11	11	4	4
L9+S11	6	4	4
L9+S6+S11	12	5	5/4[e]

[a] Number of solvent-accessible crosslinks identified following pLink[1] analysis.
[b] Number of crosslinks identified following DisVis[2] analysis filtering for End-to-End, E-E, and Side-to-Side, S-S, binding mode.
[c] No filtered crosslinks identified.
[d] Intact ribosome model (PDB entry 5UYK) lacks part of S6, including five crosslinked residues.
[e] In addition to one subunit binding to L9 and to S6 and both subunits binding to S11, a possible 'Side-to-Side' mode exists (Side-to-Side2), in which one subunit binds to L9 and both subunits bind to S6 and S11 when three RPs are involved in binding.

[1] Chen, Z.-L.; Meng, J.-M.; Cao, Y.; Yin, J.-L.; Fang, R.-Q.; Fan, S.-B.; Liu, C.; Zeng, W.-F.; Ding, Y.-H.; Tan, D.; Wu, L.; Zhou, W.-J.; Chi, H.; Sun, R.-X.; Dong, M.-Q.; He, S.-M., A high-speed search engine pLink 2 with systematic evaluation for proteome-scale identification of cross-linked peptides. *Nature Communications* **2019,** *10* (1).

[2] Van Zundert, G. C. P.; Bonvin, A. M. J. J., DisVis: quantifying and visualizing accessible interaction space of distance-restrained biomolecular complexes. *Bioinformatics* **2015,** *31* (19), 3222-3224.

Table S3. Filtered distance restraints and predicted interacting residues for TPI-ribosome docking.

RPs	Possible Binding Modes	Predicted Interacting Residues of RPs [a]	Predicted Interacting Residues of TPI [b]	Filtered Distance Restraints [c]
L9 [d]	End-to-End	K71, I72, A74, L75, T77, T79, A81, A105, A106, E149, P177, G178, E181, A185, T188, G189, E191	D132, E135, A136, G137, I138, K141, G173, K174, T175, Q179, T194	TPI(K130)-L9(K83)
	Side-to-Side	R68, E70, K71, I72, A74, L75, T77, T79, T104, A105, A106, E109, E149, E174, P177, G178, E181, T184, A185, T188, G189, E191, K193	E107, D132, E135, A136, G137, I138, K141, E145, D152, G173, E414, D439, E442, A443, G444, I445, K448, E452	TPI(K130)-L9(K83)<br>TPI(K437)-L9(K83)
L10 [e]	End-to-End	V12, S16, K20, A22, P68, F69, E70, L72, K73, A75, F76, P89, G90, A91, A120, Q122, I123, D124, L126, A127, T128, P130, T131	E135, A136, G137, G171, G173, K174, T175, T177, Q179, E183, T213, G215, K218, E219, S222, H224, P238, D242, K247, H248	TPI(K174)-L10(K73)<br>TPI(K247)-L10(K20)<br>TPI(K218)-L10(K20)
	Side-to-Side	L5, K8, Q9, A10, V12, A13, S16, K20, A22, A48, P68, F69, E70, L72, K73, P89, G90, A91, A120, T128, P130, T131	Q53, A57, P70, K71, A81, K84, G87, A89, A114, A118, G120, K155, D156, K159, D324, K326, S327, E330, H333, T334, G337, K361, K544, P545	TPI(K247)-L10(K20)<br>TPI(K481)-L10(K73)<br>TPI(K554)-L10(K20)<br>TPI(K525)-L10(K20)
L11 [e]	End-to-End	M156, A157, N158, K179, A183, K184, D186, S187, I188, E189, K190, K221, I225, K226, S227, A243, Q244, E247, Q250, T251	S20, E23, T27, K32, I170, G171, G173, K174, T175, T177, S211, T213, G215, K218, E219, S222, G233, K237, P238, D242, K247, H248, V376, P377, K378, A380	TPI(K58)-L11(K44)<br>TPI(K32)-L11(K50)<br>TPI(K174)-L11(K50)<br>TPI(K174)-L11(K39)<br>TPI(K237)-L11(K44)<br>TPI(K237)-L11(K39)<br>TPI(K130)-L11(K86)<br>TPI(K54)-L11(K44)<br>TPI(K247)-L11(K50)<br>TPI(K32)-L11(K99)
	Side-to-Side [f]	M156, A157, N158, S160, M175, E176, K179, A183, K184, D186, S187, I188, E189, K190, S205, T207, K221, Q244, E247, Q250, T251, A254	D17, K19, S20, E23, H26, G30, Q53, K54, D56, A57, K84, G87, D324, K326, S327, E330, H333, T334, G337, K339, Q360, K361, D363, A364, K365, K391, G394	TPI(K58)-L11(K44)<br>TPI(K32)-L11(K50)<br>TPI(K237)-L11(K44)<br>TPI(K237)-L11(K39)<br>TPI(K54)-L11(K44)<br>TPI(K365)-L11(K44)<br>TPI(K339)-L11(K50)<br>TPI(K544)-L11(K44)<br>TPI(K544)-L11(K39)<br>TPI(K361)-L11(K44)

Continued page of Table S3

				TPI(K554)-L11(K50)<br>TPI(K339)-L11(K99)
**L15** [g]	End-to-End	E86, G87, V89, E115, T117, T118, P119, T121, R123, R132, A133, E136, A137, G139, K141, E144	D132, E135, A136, G137, I138, K141, G173, K174, T175, Q179, T194, G215, E219, H248	TPI(K174)-L15(K141)
	Side-to-Side	K84, E86, G87, V89, E115, T117, T118, P119, T121, R123, K129, R132, A133, E136, A137, G139, K141, I142, E144	V101, G103, S105, D106, E107, Q111, A114, K130, D132, I136, I138, K141, E145, K148, D152, G410, S412, D413, E414, Q418, K437, D439, A443, I445, K448, E452, K455, D459	TPI(K174)-L15(K141)<br>TPI(K481)-L15(K141)
**L18** [h]	End-to-End	K56, A57, A59, E60, Q61, K63, K68, D69, A72, A73, K76, E80, R81, L83, E84, K85, G86, K88, D89, D108, E112, G114, Q116	P3, E135, A136, G137, G171, G173, K174, T175, T177, Q179, E183, E186, G190, K193, T194, D198, T213, G215, K218, E219, S222, H224, H248	TPI(K247)-L18(K68)<br>TPI(K130)-L18(K88)<br>TPI(K130)-L18(K76)<br>TPI(K218)-L18(K76)
	Side-to-Side	K56, A57, A59, E60, Q61, K63, K68, D69, A72, A73, K76, E80, R81, L83, E84, K85, G86, K88, D89, Q104, D108, E112, G114, Q116	P70, K71, A73, E107, Q111, A114, A118, D152, E414, K437, D439, E442, A443, G444, I445, E447, K448, E452, K455, D459, I477, G478, G480, H502	TPI(K130)-L18(K88)<br>TPI(K130)-L18(K76)<br>TPI(K554)-L18(K68)<br>TPI(K437)-L18(K88)<br>TPI(K437)-L18(K76)<br>TPI(K525)-L18(K76)
**L20** [i]	End-to-End	G72, V103, E110, K113, A114, A117	A2, P3, G30, K32, A35, H248	TPI(K32)-L20(K111)
	Side-to-Side	Q70, G72, K84, S86, K102, V103, T106, E110, K113, A114, A117	K19, Q53, K54, K326, Q360, K361	TPI(K32)-L20(K111)<br>TPI(K339)-L20(K111)
**L22** [j]	End-to-End	E59, H60, N61, D62, G63, D65, I66, D67, D68	A2, P3, G30, K32, A35, G215, H248	TPI(K218)-L22(K70)
**S6** [d]	End-to-End	L75, E174, P177, D199, ^{R2}02, T255, E256, S258	A2, K32, G215, E219, H248	TPI(K218)-S6(K56)
**S8** [k]	End-to-End	K30, V33, N37, K40, E41, D47, K49, V50, K107, R113, A114, Q117	A2, P3, K5, A35, D36, D198, A199, Q202, S203, S222, H224, K247, H248	TPI(K155)-S8(K30)<br>TPI(K237)-S8(K88)<br>TPI(K130)-S8(K107)<br>TPI(K141)-S8(K107)
	Side-to-Side	V33, N37, K40, E41, E46, D47, K49, V50, K107, R113, A114, Q117	K437, D439, E442, A443, G444, I445, K448, G480	TPI(K155)-S8(K30)<br>TPI(K130)-S8(K107)<br>TPI(K141)-S8(K107)<br>TPI(K462)-S8(K30)<br>TPI(K544)-S8(K88)<br>TPI(K437)-S8(K107)<br>TPI(K448)-S8(K107)

Continued page of Table S3

**S11** [l]	End-to-End	Q204, Q270, S272, R292, D327, K330, K335, N336, R353, N356, A357, G359, R361	D132, E135, A136, G137, I138, K141, E145, K148, D152, K155, G190, K193, T194, H195, D198, A199	TPI(K68)-S11(K74)<br>TPI(K130)-S11(K79)<br>TPI(K130)-S11(K74)
	Side-to-Side	D171, R202, Q204, L212, H213, K214, K269, Q270, S272, R292, Q293, E323, K335, N336, R353, N356, A357, G359, R361	E107, D132, E135, A136, I138, G171, E414, D439, E442, A443, I445	TPI(K68)-S11(K74)<br>TPI(K130)-S11(K79)<br>TPI(K130)-S11(K74)<br>TPI(K375)-S11(K74)<br>TPI(K437)-S11(K79)<br>TPI(K437)-S11(K74)
**L10+L11** [e]	End-to-End	R46, A183, K184, D186, S187, I188, E189, K221, R242, A243, Q244, E247, Q250, T251, A254, D260, I261, E262	S20, T27, G30, K32, G171, G173, K174, T175, T177, Q179, S211, T213, G215, K218, E219, S222, G233, K237, P238, D242, K247, H248, P377, K378	TPI(K247)-L10(K20)<br>TPI(K218)-L10(K20)<br>TPI(K58)-L11(K44)<br>TPI(K32)-L11(K50)<br>TPI(K174)-L11(K50)<br>TPI(K174)-L11(K39)<br>TPI(K237)-L11(K44)<br>TPI(K237)-L11(K39)<br>TPI(K130)-L11(K86)<br>TPI(K54)-L11(K44)<br>TPI(K247)-L11(K50)<br>TPI(K32)-L11(K99)
	Side-to-Side	R46, A48, P89, G90, A91, R94, K101, L126, A127, T128, P130, T131, A183, K184, D186, S187, I188, E189, K190, K221, A243, Q244, E247, Q250, T251, A254, D260, I261, E262	A2, P3, K5, K32, A35, D36, Q53, K54, D56, A57, K58, P70, K71, A81, K84, G87, A89, A118, G120, K155, D156, S158, K159, D198, A199, Q202, S203, D324, K326, S327, E330, H333, T334, G337, K339, Q360, K361, D363, K544, P545, D549	TPI(K247)-L10(K20)<br>TPI(K218)-L10(K20)<br>TPI(K365)-L11(K44)<br>TPI(K339)-L11(K50)<br>TPI(K481)-L11(K50)<br>TPI(K481)-L11(K39)<br>TPI(K544)-L11(K44)<br>TPI(K544)-L11(K39)<br>TPI(K361)-L11(K44)<br>TPI(K554)-L11(K50)<br>TPI(K339)-L11(K99)
**L15+L18** [m]	End-to-End	K228, E232, R233, L235, E236, K237, G238, K240, D241, D260, E264, G266, Q268	A2, P3, E186, K187, G190, K193, T194, H195, D198, A199, Q202, S203, H224	TPI(K174)-L15(K141)<br>TPI(K247)-L18(K68)<br>TPI(K130)-L18(K88)<br>TPI(K130)-L18(K76)<br>TPI(K218)-L18(K76)
	Side-to-Side	K129, R132, A133, E136, A137, K228, E232, R233, L235, E236, K237, G238, K240, D241, D260, E264, G266, Q268	A309, P310, E442, A443, G444, G480, T484, Q486, E490, E493, K494, K500,	TPI(K174)-L15(K141)<br>TPI(K554)-L18(K68)<br>TPI(K437)-L18(K88)

Continued page of Table S3

			T501, H502, S504, D505, A506, Q509, S510, G522, K525, S529, H531	TPI(K437)-L18(K76)<br>TPI(K525)-L18(K76)
**L9+S6** [d]	End-to-End	A74, L75, T77, T79, A81, A106, E127, K141, I143, N145, A148, E149, D171, E174, Q175, P177, G178, E181, T184, A185, T188, G189, D199, R202	D132, E135, A136, G137, I138, G171, G173, K174, T175, T177, Q179, E183, T194, G215, K218, E219, S222, H224, H248	TPI(K130)-L9(K83)<br>TPI(K218)-S6(K56)
	Side-to-Side	K71, A74, L75, T77, T79, A81, A105, A106, E127, K141, I143, N145, A148, E149, D171, E174, Q175, P177, G178, E181, T184, A185, T188, K193, D199, R202, Q204, L212, H213, K214, S258	P70, G103, S105, D106, E107, Q111, A114, A118, D132, E145, D152, K155, G410, S412, E414, K437, D439, E442, A443, G444, I445, K448, E452, G478, G480	TPI(K130)-L9(K83)<br>TPI(K525)-S6(K56)
**S6+S11** [d]	End-to-End	D171, R202, Q204, L212, H213, K214, K269, Q270, S272, R292, Q293, K335, N336, R361	A2, A136, G137, I138, K141, D152, K155, D156, G190, K193, T194, H195, D198, A199	TPI(K218)-S6(K56)<br>TPI(K68)-S11(K74)<br>TPI(K130)-S11(K79)<br>TPI(K130)-S11(K74)
	Side-to-Side	R202, Q204, L212, H213, K214, S258, S272, R292, K335, N336, A357, G359, R361	D132, E135, A136, G137, I138, T194, E414	TPI(K218)-S6(K56)<br>TPI(K375)-S11(K74)<br>TPI(K437)-S11(K79)<br>TPI(K437)-S11(K74)
**L9+S11** [d]	End-to-End	S14, P118, N119, E127, K141, D171, E174, R202, Q204, L212, H213, K214, K269, Q270, S272, R292, Q293, K335, N336	E135, A136, G137, G173, K174, T175, T177, Q179, E183, G190, K193, T194, H195, D198, D459, H502	TPI(K130)-L9(K83)<br>TPI(K68)-S11(K74)<br>TPI(K130)-S11(K79)<br>TPI(K130)-S11(K74)
	Side-to-Side	A74, L75, E127, K141, I143, D171, E174, R202, K214, S272, K335, N336, G359, R361	D132, E135, A136, G137, I138, E140, K141, Q179, E183, T501, H502	TPI(K130)-L9(K83)<br>TPI(K375)-S11(K74)<br>TPI(K437)-S11(K79)<br>TPI(K437)-S11(K74)
**L9+S6+S11** [d]	End-to-End	S14, P118, N119, E127, K141, D171, E174, R202, Q204, L212, H213, K214, K269, Q270, S272, R292, Q293, K335, N336	E135, A136, G137, G173, K174, T175, T177, Q179, E183, G190, K193, T194, H195, D198, D459, H502	TPI(K130)-L9(K83)<br>TPI(K218)-S6(K56)<br>TPI(K68)-S11(K74)<br>TPI(K130)-S11(K79)<br>TPI(K130)-S11(K74)
	Side-to-Side	E127, H169, D171, E174, Q175, P177, D199, R202, Q204, L212, H213, K214, S258, K335, G359, R361	D132, E135, A136, G137, I138, E140, K141, E145, Q179, K187, G190, T194, H195	TPI(K130)-L9(K83)<br>TPI(K218)-S6(K56)<br>TPI(K375)-S11(K74)<br>TPI(K437)-S11(K79)<br>TPI(K437)-S11(K74)
	Side-to-Side2	K71, I72, A74, L75, T77, T79, A81, A106, E127, I143, N145, A148, E149, E174, Q175, P177, G178, E181, D199, R202,	G103, S105, D106, E107, Q111, A114, A118, K130, D132, E135, A136, G137, I138, K141, E145, K148, D152, K155,	TPI(K130)-L9(K83)<br>TPI(K525)-S6(K56)

Continued page of Table S3

	Q204, K211, L212, H213, K214, S258, K269, Q270, S272, R292, Q293, N336	H195, D439, E442, A443, G444, I445, G478, G480, T484, Q486, E490	TPI(K437)-S11(K79) TPI(K437)-S11(K74)

[a] RP models including nearby rRNAs were obtained from ribosomal PDB file 5UYK and re-numbered into a single chain A for DisVis and HADDOCK analyses.
[b] Residue numbers of TPI from PDB file 8TIM were re-numbered into a single chain B (2–248 as monomer A and 309-555 as monomer B) to represent the TPI dimer for DisVis and HADDOCK analyses.
[c] All sets of Cα-Cα distance restraints were identified by an initial coarse scan of DisVis and ranged from 0 to 34.5 Å. A 3 Å upper limit was input as unambiguous restraints for HADDOCK analysis.
[d] Input residues: L9 (1-149) + S6 (159-258) + S11 (268-383) + S18 (393-457) + S21 (467-531) + 23S rRNA [151-172, 1411-1419, 1484-1506, 1576-1587, 2095-2109, 2134-2155, 2179-2191] + 16S rRNA [658-685, 707-716, 735-742].
[e] Input residues: L10 (1-131) + L11 (141-281) + 23S rRNA [1040-1115]
[f] Input residues: L10 (1-131) + L11 (141-281) + 23S rRNA [1040-1115] + 16S rRNA [511-544, 949-964, 983-1051, 1200-1221]
[g] Input residues: L15 (2-144) + 23S rRNA [226-262, 602-656, 2401-2412]
[h] Input residues: L18 (2-117) + 5S rRNA [1-64, 108-120] + 23S rRNA [2366-2381]
[i] Input residues: L20 (1-117) + L21 (127-229) + L30 (239-296) + 23S rRNA [538-550, 992-1081, 1142-1185, 2893-2903]
[j] Input residues: L22 (1-110) + L32 (120-175) + 23S rRNA [13-14, 303-316, 482-560, 1208-1239, 1279-1288, 2784-2811]
[k] Input residues: S8 (1-129) + S5 (139-295) + 16S rRNA [2-7,584-657, 742-759, 824-856]
[l] Input residues: L9 (1-149) + S6 (159-258) + S11 (268-383) + S17 (393-543) + S18 (553-617) + S21 (627-691) + L1 (701-923) + 23S rRNA [2089-2193] + 16S rRNA [664-725, 1531-1540]
[m] Input residues: L15 (2-144) + L18 (154-269) + 23S rRNA [602-656, 916-940, 2345-2381] + 16S rRNA [1-64, 108-120].
Re-numbered RPs residues are in parentheses and original rRNA residues are in square brackets.

Table S4. TPI-ribosome binding conformations.

RPs	Possible Binding Modes	HADDOCK Settings[a]	# of Structures Generated[b]	# of Clusters	Order of the Best Cluster	HADDOCK Score[c]	Cluster Size	RMSD from the Lowest-Energy Structure	ID of the Best Model	Fnat[d]	i-RMSD (Å)[e]	l-RMSD (Å)[f]	CAPRI Assessment[g]
**L9**	End-to-End	default no discard	200	3	1	-51.6 ± 2.5	123	1.9 ± 1.1	11	1.0	0.0	0.0	***
	Side-to-Side	it0 fixed no discard	186	8	1	7.2 ± 3.5	89	4.0 ± 1.4	115	0.6	0.9	5.0	**
**L10**	End-to-End	default no discard	200	5	2	-51.6 ± 2.5	75	0.6 ± 0.3	116	1.0	0.0	0.0	***
	Side-to-Side	it0 fixed no discard	198	4	2	-43.1 ± 1.2	60	0.3 ± 0.2	8	1.0	0.0	0.0	***
**L11**	End-to-End	default no discard	200	4	1	-33.6 ± 2.0	162	0.5 ± 0.3	113	1.0	0.0	0.0	***
	Side-to-Side	it0 fixed no discard	198	3	1	-32.9 ± 2.4	176	1.2± 0.1	149	0.9	0.1	1.0	***
**L15**	End-to-End	default no discard	200	3	2	-22.9 ± 6.0	38	0.7 ± 0.5	196	1.0	0.0	0.0	***
	Side-to-Side	it0 fixed no discard	198	8	3	-55.6 ± 3.4	33	0.6 ± 0.3	191	1.0	0.0	0.0	***
**L18**	End-to-End	it0 fixed no discard	193	2	1	-36.8 ± 5.1	151	0.9 ± 0.6	124	1.0	0.0	0.0	***
	Side-to-Side	default	152	12	1	-41.7 ± 6.7	79	1.6 ± 1.4	61	1.0	0.0	0.0	***
**L20**	End-to-End	default no discard	195	3	3	-40.9 ± 5.2	9	1.4 ± 0.8	91	1.0	0.0	0.0	***
	Side-to-Side	it0 fixed no discard	197	11	2	-76.7 ± 5.1	38	1.2 ± 0.7	198	1.0	0.0	0.0	***
**L22**	End-to-End	default no discard	189	3	1	-65.3 ± 5.0	168	2.1 ± 1.3	57	1.0	0.0	0.0	***
**S6**	End-to-End	default no discard	196	3	1	-101.0 ± 2.2	179	1.9 ± 1.1	85	1.0	0.0	0.0	***
**S8**	End-to-End	it0 fixed no discard	196	5	1	-93.1 ± 3.7	93	0.8 ± 0.5	87	1.0	0.0	0.0	***
	Side-to-Side	it0 fixed	197	3	1	-48.5 ± 0.9	101	0.9 ± 0.5	18	1.0	0.0	0.0	***
**S11**	End-to-End	default no discard	155	15	7	-76.5± 12.2	12	1.5 ± 1.0	89	1.0	0.0	0.0	***

Continued page of Table S4

	Side-to-Side	it0 fixed no discard	195	5	2	-113.9± 9.2	40	1.0 ± 0.6	41	1.0	0.0	0.0	***
**L10+L11**	End-to-End	it0 fixed	200	6	1	-62.4 ± 8.6	138	0.7 ± 0.4	3	1.0	0.0	0.0	***
	Side-to-Side	it0 fixed	180	9	1	-89.7± 3.6	65	0.4 ± 0.3	10	1.0	0.0	0.0	***
**L15+L18**	End-to-End	it0 fixed	182	7	2	-78.6 ± 4.4	31	1.3 ± 0.8	24	1.0	0.0	0.0	***
	Side-to-Side	default no discard	199	1	1	14.1 ± 0.5	199	0.9 ± 0.5	193	1.0	0.0	0.0	***
**L9+S6**	End-to-End	default	172	13	1	-87.0 ± 3.9	29	1.8 ± 1.0	56	1.0	0.0	0.0	***
	Side-to-Side	it0 fixed no discard	198	2	1	-54.7 ± 6.7	191	2.4 ± 0.2	193	0.8	0.7	3.0	**
**S6+S11**	End-to-End	it0 fixed no discard	199	3	1	-88.8 ± 6.8	159	2.0 ± 1.2	49	1.0	0.0	0.0	***
	Side-to-Side	default no discard	199	3	1	-108.0 ± 2.0	161	1.7 ± 1.0	124	1.0	0.0	0.0	***
**L9+S11**	End-to-End	it0 fixed no discard	200	1	1	-98.6 ± 10.8	200	1.8 ± 1.1	137	1.0	0.0	0.0	***
	Side-to-Side	it0 fixed no discard	194	3	2	-80.1 ± 4.7	61	1.8 ± 1.1	46	1.0	0.0	0.0	***
**L9+S6+S11**	End-to-End	it0 fixed no discard	200	2	1	-97.7 ± 13.3	189	1.9 ± 1.1	107	1.0	0.0	0.0	***
	Side-to-Side	it0 fixed no discard	196	4	4	-53.0 ± 1.7	17	1.9 ± 1.1	60	1.0	0.0	0.0	***
	Side-to-Side2	it0 fixed no discard	200	4	1	-141.5 ± 4.5	123	1.5 ± 0.9	83	1.0	0.0	0.0	***

[a] The HADDOCK setting results in the best cluster. Exported from HADDOCK Analysis.
[b] Maximum of generated structures is 200 after the water stage of HADDOCK.
[c] A weighted sum of various energies and buried surface area in the three docking stages (it0, it1, water)[1].
[d] Fraction of native contact
[e] Interface root mean square deviation
[f] Ligand root mean square deviation
[g] Critical assessment of predicted interactions assessment criteria[2]: High (***): Fnat ≥ 0.5 and i-RMSD ≤ 1 Å and l-RMSD ≤ 1 Å; Medium (**): 0.3 ≤ Fnat < 0.5 and (1 Å < i-RMSD ≤ 2 Å or 1 Å < l-RMSD ≤ 5 Å); Acceptable (*): 0.1 ≤ Fnat < 0.3 and (2 Å < i-RMSD ≤ 4 Å or 5 Å < l-RMSD ≤ 10 Å); Incorrect: Fnat < 0.1 or i-RMSD > 6 Å and l-RMSD > 10 Å.

1. De Vries, S. J.; Van Dijk, A. D. J.; Krzeminski, M.; Van Dijk, M.; Thureau, A.; Hsu, V.; Wassenaar, T.; Bonvin, A. M. J. J., HADDOCK versus HADDOCK: New features and performance of HADDOCK2.0 on the CAPRI targets. *Proteins: Structure, Function, and Bioinformatics* **2007,** *69* (4), 726-733.

Continued page of Table S4

2. Lensink, M. F.; Wodak, S. J., Docking and scoring protein interactions: CAPRI 2009. *Proteins: Structure, Function, and Bioinformatics* **2010,** *78* (15), 3073-3084.

Table S5. Orthogonal axes originating at the C2 of substrates in Cartesian coordinates.

RPs	Possible Binding Modes	DHAP1											
		a_x	a_y	a_z	b_x	b_y	b_z	c_x	c_y	c_z	d_x	d_y	d_z
**L9**	End-to-End	-0.9215	0.1539	-0.3567	0.0570	-0.4164	0.9074	-0.0089	0.8158	0.3750	0.3487	0.3487	-0.7503
	Side-to-Side	-0.0845	0.9022	-0.4230	-0.0227	-0.0207	0.9995	0.8930	0.0940	0.0222	0.0598	-0.3759	-0.8136
**L10**	End-to-End	0.5682	0.4323	0.7002	-0.0304	0.4604	-0.8872	-0.7059	0.4828	0.2748	-0.2193	-0.6504	0.5795
	Side-to-Side	-0.5567	-0.7700	0.3117	-0.1671	0.8755	0.4533	-0.6220	0.2003	-0.6161	0.4120	-0.5368	-0.5904
**L11**	End-to-End	0.7184	-0.6728	0.1769	-0.6017	-0.1917	-0.7753	0.5556	0.4505	-0.5426	0.2853	0.4881	0.6974
	Side-to-Side	0.7623	0.6099	-0.2165	0.0104	-0.4108	0.9117	0.4671	-0.6973	-0.3195	-0.3458	0.1424	-0.8164
**L15**	End-to-End	0.0878	0.9194	0.3835	-0.0790	-0.7466	0.6605	0.8936	-0.0883	0.0071	0.0404	0.3420	-0.8293
	Side-to-Side	0.3261	-0.4417	-0.8358	-0.2428	-0.6099	0.7543	-0.8430	-0.0431	-0.3062	0.0992	0.8044	-0.3864
**L18**	End-to-End	0.1083	0.9249	0.3644	-0.8831	-0.4383	0.1677	0.3148	-0.3400	0.7693	0.8354	0.0314	-0.3280
	Side-to-Side	0.4237	0.0198	-0.9056	0.3040	-0.7297	0.6124	-0.6487	-0.5348	-0.3152	-0.4905	0.7210	-0.2138
**L20**	End-to-End	-0.3366	0.9056	0.2581	0.4335	-0.5326	0.7270	0.7958	0.3566	-0.2132	-0.2851	0.1336	-0.8407
	Side-to-Side	-0.5599	-0.8286	0.0007	0.2760	0.3460	0.8967	-0.7432	0.5022	0.0350	-0.0293	0.0191	-0.8970
**L22**	End-to-End	-0.4338	0.8775	0.2047	-0.2804	-0.7733	0.5686	0.6572	0.1893	0.5815	0.4715	0.3868	-0.6588
**S6**	End-to-End	0.4607	-0.4923	-0.7385	0.5277	0.7264	0.4403	0.3197	-0.5925	0.5944	-0.7302	-0.5100	-0.1155
**S8**	End-to-End	0.9317	0.0244	-0.3625	-0.7325	-0.0894	-0.6749	-0.0489	0.8943	-0.0654	0.3226	0.0786	0.8344
	Side-to-Side	0.8538	0.0376	0.5193	-0.2998	0.8587	-0.4157	-0.4615	0.1993	0.7444	-0.0755	-0.8752	0.1875
**S11**	End-to-End	-0.6088	0.6240	-0.4899	-0.0609	-0.9650	-0.2552	-0.6320	-0.1255	0.6255	0.3288	0.6904	0.4708
	Side-to-Side	0.8546	-0.5181	0.0350	-0.4936	0.0933	0.8647	-0.4512	-0.7563	-0.1760	0.1176	0.1346	-0.8801
**L10+L11**	End-to-End	0.3411	-0.5628	0.7529	0.2610	-0.2908	-0.9205	0.7371	0.5105	0.0477	-0.4112	0.5386	0.5890
	Side-to-Side	-0.2620	-0.7973	-0.5438	0.7458	-0.1374	0.6518	-0.5944	-0.2348	0.6306	-0.6304	0.4885	-0.4124
**L15+L18**	End-to-End	0.5935	0.8035	-0.0462	-0.9734	0.1800	0.1418	0.1223	-0.0392	0.8890	0.7124	-0.5333	-0.1215
	Side-to-Side	0.9200	-0.3788	-0.1003	-0.7548	-0.6518	-0.0739	-0.0374	0.1437	-0.8856	0.3499	0.8185	0.1180
**L9+S6**	End-to-End	-0.2760	-0.6326	-0.7237	0.7236	-0.3187	0.6122	-0.6179	-0.3547	0.5457	-0.6019	0.5978	-0.2930
	Side-to-Side	0.7574	-0.1565	0.6339	-0.7600	0.5481	0.3492	-0.4021	-0.7463	0.2962	0.4267	-0.4793	-0.6282
**S6+S11**	End-to-End	0.7763	-0.6198	-0.1149	0.1885	0.8596	0.4748	-0.1956	-0.3903	0.7842	-0.5309	-0.5863	-0.4242
	Side-to-Side	0.8311	-0.5551	0.0343	-0.4116	0.2314	0.8815	-0.4973	-0.7467	-0.0362	0.0457	0.0130	-0.8966
**L9+S11**	End-to-End	0.4118	0.9113	0.0073	-0.6104	-0.2138	0.7627	0.6966	-0.3185	0.4682	0.4290	-0.1877	-0.7659
	Side-to-Side	0.8746	-0.3710	-0.3122	-0.3381	-0.3478	0.8745	-0.4330	-0.6592	-0.4296	-0.0464	0.5110	-0.7372
**L9+S6+S11**	End-to-End	0.4291	0.9031	-0.0178	-0.5727	-0.2002	0.7949	0.7143	-0.3309	0.4313	0.3836	-0.1978	-0.7871
	Side-to-Side	0.8869	-0.3368	-0.3163	-0.1244	0.0492	0.9910	-0.3182	-0.8396	0.0018	-0.2662	0.0991	-0.8517
	Side-to-Side2	0.4832	-0.3769	0.7902	-0.5392	0.8257	0.1659	-0.7150	-0.5063	0.1957	0.3263	-0.6596	-0.5141

Continued page of Table S5

RPs	Possible Binding Modes	DHAP2											
		a_x	a_y	a_z	b_x	b_y	b_z	c_x	c_y	c_z	d_x	d_y	d_z
**L9**	End-to-End	0.9065	-0.1617	0.3901	-0.5442	0.7154	0.4381	-0.3499	-0.6094	0.5606	0.1471	-0.6446	-0.6090
	Side-to-Side	0.0890	-0.8850	0.4569	0.7753	0.6237	0.0996	-0.3731	0.3454	0.7417	-0.8143	-0.2365	-0.2995
**L10**	End-to-End	-0.5641	-0.4011	-0.7218	-0.3262	0.8646	0.3822	0.4707	0.4510	-0.6185	0.5736	-0.6887	-0.0656
	Side-to-Side	0.5343	0.7935	-0.2914	-0.9716	0.0156	-0.2362	-0.1829	0.4093	0.7793	0.7376	-0.3631	0.3638
**L11**	End-to-End	-0.7250	0.6557	-0.2107	0.7500	-0.0495	-0.6595	-0.4429	-0.6362	-0.4559	-0.4330	-0.2372	0.7517
	Side-to-Side	-0.7439	-0.6208	0.2475	0.8988	-0.4204	-0.1243	0.1812	0.1300	0.8707	-0.5727	0.6926	0.0158
**L15**	End-to-End	-0.0818	-0.9336	-0.3489	0.8605	0.2226	0.4582	-0.3501	-0.2628	0.7851	-0.8246	0.1864	-0.3054
	Side-to-Side	-0.3365	0.4072	0.8491	-0.6717	-0.5103	-0.5370	0.2146	-0.7511	0.4452	0.8190	0.3321	0.1653
**L18**	End-to-End	-0.1414	-0.9282	-0.3443	0.0638	0.0930	0.9936	-0.8902	0.1185	0.0460	-0.0019	0.3130	-0.8430
	Side-to-Side	-0.4085	-0.0537	0.9112	-0.2480	-0.7315	-0.6352	0.7006	-0.4854	0.2855	0.4269	0.7550	0.2359
**L20**	End-to-End	0.3546	-0.9075	-0.2251	0.6848	0.7010	0.1992	-0.0230	-0.2248	0.8700	-0.8401	-0.3034	-0.1006
	Side-to-Side	0.5549	0.8311	0.0366	-0.9392	0.0853	0.3326	0.2733	-0.2190	0.8279	0.6961	-0.4494	-0.3487
**L22**	End-to-End	0.4189	-0.8914	-0.1728	0.2590	0.4470	0.8562	-0.6861	-0.4034	0.4181	-0.4424	-0.0566	-0.7806
**S6**	End-to-End	-0.4271	0.5090	0.7474	0.7522	-0.6071	0.2564	0.5842	0.6716	-0.1236	-0.5648	0.3838	-0.5842
**S8**	End-to-End	-0.9447	-0.0209	0.3271	0.2708	0.8195	-0.5051	-0.2575	-0.3886	-0.7685	0.1432	-0.8103	0.3618
	Side-to-Side	-0.8535	0.0007	-0.5210	-0.0169	0.4952	0.8686	0.2586	0.7502	-0.4226	0.3906	-0.4954	-0.6405
**S11**	End-to-End	0.5896	-0.6531	0.4751	-0.9771	-0.0638	0.2031	-0.1023	-0.5840	-0.6758	0.7188	0.3498	-0.4112
	Side-to-Side	-0.8624	0.5062	0.0007	-0.0709	-0.9867	0.1464	0.0748	0.1262	0.8868	0.4488	0.7649	-0.1467
**L10+L11**	End-to-End	-0.3180	0.5445	-0.7761	0.9808	0.0404	0.1907	0.1351	-0.7006	-0.5469	-0.8415	-0.2788	0.1492
	Side-to-Side	0.2835	0.7744	0.5656	-0.4634	-0.7459	0.4784	0.7924	-0.3977	0.1475	0.3391	0.4064	-0.7264
**L15+L18**	End-to-End	-0.6219	-0.7809	0.0583	0.1471	0.5070	0.8493	-0.6928	0.5367	-0.2004	0.1252	-0.1650	-0.8748
	Side-to-Side	-0.9342	0.3457	0.0877	0.2668	-0.3124	-0.9117	-0.2878	-0.8284	0.1996	0.1417	0.1612	0.8734
**L9+S6**	End-to-End	0.2958	0.6047	0.7395	-0.5012	-0.8195	0.2779	0.7741	-0.4528	0.0607	0.3716	0.5545	-0.6020
	Side-to-Side	-0.7781	0.1701	-0.6047	-0.1731	-0.6078	0.7750	-0.2356	0.7077	0.5024	0.5134	0.5334	-0.5106
**S6+S11**	End-to-End	-0.7553	0.6407	0.1381	0.3488	-0.4503	0.8219	0.5888	0.6689	0.1166	-0.0177	0.1694	-0.8825
	Side-to-Side	-0.8363	0.5483	0.0026	-0.1009	-0.9541	0.2818	0.1570	0.2354	0.8532	0.4672	0.7139	-0.2830
**L9+S11**	End-to-End	-0.4236	-0.9054	0.0282	0.6945	0.1803	0.6965	-0.6357	0.3146	0.5525	-0.5091	0.2161	-0.7088
	Side-to-Side	-0.8754	0.3438	0.3398	0.0104	-0.9549	-0.2968	0.2225	-0.2563	0.8323	0.3732	0.8042	0.1479
**L9+S6+S11**	End-to-End	-0.4392	-0.8968	0.0530	0.7344	0.1680	0.6576	-0.5986	0.3278	0.5848	-0.5419	0.2251	-0.6808
	Side-to-Side	-0.8776	0.3259	0.3514	0.1966	-0.9701	0.1426	0.3874	0.1942	0.7873	0.1883	0.8271	-0.2967
	Side-to-Side2	-0.5022	0.4000	-0.7667	-0.5564	-0.4243	0.7145	-0.0395	0.7853	0.4356	0.7764	0.2490	-0.3786

Continued page of Table S5

RPs	Possible Binding Modes	GAP1											
		a_x	a_y	a_z	b_x	b_y	b_z	c_x	c_y	c_z	d_x	d_y	d_z
**L9**	End-to-End	-0.9410	0.0476	-0.3352	0.2755	-0.8718	0.4051	-0.2729	0.2888	0.8072	0.1352	0.8510	-0.2588
	Side-to-Side	-0.1742	0.9152	-0.3634	-0.6157	-0.3072	0.7257	0.5526	0.3501	0.6170	0.6919	-0.0933	-0.5667
**L10**	End-to-End	0.6492	0.4306	0.6270	0.3285	-0.1538	-0.9319	-0.3049	0.8110	-0.2413	-0.6124	-0.0345	0.6577
	Side-to-Side	-0.5234	-0.7453	0.4131	0.4680	0.6107	0.6388	-0.7283	0.5277	0.0292	-0.2397	-0.2856	-0.8190
**L11**	End-to-End	0.6390	-0.7485	0.1773	-0.9511	-0.2657	-0.1577	0.1652	-0.0679	-0.8817	0.6720	0.5927	0.0803
	Side-to-Side	0.7382	0.6632	-0.1239	-0.5118	0.0713	0.8561	0.5766	-0.5685	0.3921	0.1896	-0.3609	-0.8020
**L15**	End-to-End	-0.0026	0.8973	0.4415	-0.6956	-0.6445	0.3174	0.5693	-0.3062	0.6258	0.6967	0.2530	-0.5100
	Side-to-Side	0.3980	-0.4939	-0.7731	0.3542	-0.2436	0.9029	-0.6342	-0.6332	0.0780	-0.5280	0.4593	-0.5652
**L18**	End-to-End	0.0150	0.9496	0.3131	-0.8068	-0.2744	-0.5232	-0.4110	-0.2447	0.7620	0.8003	-0.1401	0.3866
	Side-to-Side	0.5220	0.0176	-0.8528	0.5369	-0.0910	0.8387	-0.0628	-0.8956	-0.0570	-0.7648	0.0833	-0.4664
**L20**	End-to-End	-0.3907	0.8558	0.3392	-0.1951	-0.8157	0.5446	0.7427	0.1466	0.4856	0.3658	0.4416	-0.6929
	Side-to-Side	-0.4819	-0.8739	0.0639	0.8354	0.0786	0.5440	-0.4804	0.3156	0.6921	-0.6250	0.3029	-0.5719
**L22**	End-to-End	-0.5295	0.8255	0.1956	-0.5270	-0.8444	-0.0962	0.0857	-0.1540	0.8821	0.7583	0.4838	0.0108
**S6**	End-to-End	0.4800	-0.3952	-0.7832	-0.0046	0.9987	0.0511	0.7620	-0.0209	0.4775	-0.2051	-0.8260	0.2911
**S8**	End-to-End	0.9071	-0.0685	-0.4154	-0.6664	-0.6867	-0.2906	-0.2653	0.5404	-0.6685	0.2703	0.7166	0.4720
	Side-to-Side	0.8988	0.0822	0.4305	-0.0845	0.3968	-0.9140	-0.2459	0.7852	0.3636	-0.3081	-0.4327	0.7260
**S11**	End-to-End	-0.5675	0.5819	-0.5825	0.5563	-0.6826	-0.4738	-0.6734	-0.5930	0.0637	-0.3084	0.4284	0.7284
	Side-to-Side	0.8846	-0.4514	0.1168	-0.2147	0.7188	0.6612	-0.3824	-0.6100	0.5389	-0.1721	-0.5214	-0.7123
**L10+L11**	End-to-End	0.2981	-0.6490	0.6999	-0.4346	-0.3986	-0.8076	0.8032	-0.0634	-0.4009	0.3046	0.6816	0.5024
	Side-to-Side	-0.1558	-0.8059	-0.5712	0.9543	0.2775	0.1113	0.0688	-0.5277	0.7258	-0.8863	0.0738	0.1377
**L15+L18**	End-to-End	0.5258	0.8416	-0.1233	-0.8534	-0.0615	-0.5177	-0.4432	0.3774	0.6859	0.6238	-0.3060	0.5715
	Side-to-Side	0.8925	-0.4505	-0.0228	-0.6851	-0.4179	0.5967	-0.2783	-0.5169	-0.6816	0.2952	0.6146	-0.5867
**L9+S6**	End-to-End	-0.1695	-0.6389	-0.7504	0.9601	0.2048	0.1904	0.0320	-0.6882	0.5787	-0.8861	0.0740	0.1371
	Side-to-Side	0.7602	-0.0469	0.6480	-0.3922	0.9081	-0.1467	-0.5816	-0.1427	0.6719	0.0609	-0.8877	-0.1357
**S6+S11**	End-to-End	0.8299	-0.5350	-0.1585	0.0602	0.9744	-0.2167	0.2704	0.1703	0.8408	-0.4228	-0.7406	0.2860
	Side-to-Side	0.8709	-0.4802	0.1044	-0.1243	0.8081	0.5758	-0.3608	-0.5145	0.6441	-0.2556	-0.5986	-0.6213
**L9+S11**	End-to-End	0.3105	0.9504	0.0196	-0.9774	-0.1440	0.1545	0.1497	-0.0671	0.8842	0.8416	-0.2716	-0.1631
	Side-to-Side	0.9143	-0.3430	-0.2152	-0.1329	0.3340	0.9332	-0.2482	-0.8246	0.2598	-0.2666	-0.1841	-0.8391
**L9+S6+S11**	End-to-End	0.3295	0.9442	-0.0007	-0.9708	-0.1237	0.2055	0.1940	-0.0671	0.8758	0.8269	-0.2887	-0.2053
	Side-to-Side	0.9321	-0.2603	-0.2518	-0.0812	0.7025	0.7071	-0.0071	-0.6386	0.6337	-0.3257	-0.5889	-0.5971
	Side-to-Side2	0.5252	-0.2773	0.8045	0.0347	0.9723	-0.2311	-0.7181	0.1493	0.5203	-0.2644	-0.8510	-0.1207

Continued page of Table S5

RPs	Possible Binding Modes	GAP2											
		a_x	a_y	a_z	b_x	b_y	b_z	c_x	c_y	c_z	d_x	d_y	d_z
**L9**	End-to-End	0.8602	-0.3597	0.3616	-0.7262	-0.1230	0.6764	-0.1988	-0.8444	-0.3670	0.4373	0.2437	-0.7979
	Side-to-Side	-0.1087	-0.8555	0.5063	0.0825	0.7626	0.6415	-0.9350	0.1115	-0.0124	-0.0459	-0.4747	-0.8119
**L10**	End-to-End	-0.3833	-0.4429	-0.8105	0.3120	0.9204	-0.2355	0.8503	-0.3432	-0.2146	-0.1831	-0.7714	0.5082
	Side-to-Side	0.6223	0.7756	-0.1055	-0.7616	0.2591	0.5941	0.4881	-0.2894	0.7519	0.5527	-0.5194	-0.5587
**L11**	End-to-End	-0.8503	0.5057	-0.1454	0.1191	-0.6740	-0.7290	-0.4667	-0.6372	0.5129	0.1667	0.5040	0.7779
	Side-to-Side	-0.7991	-0.4696	0.3753	0.7486	-0.0308	0.6623	-0.2995	0.8102	0.3762	-0.4807	0.1882	-0.7881
**L15**	End-to-End	-0.2745	-0.9212	-0.2758	0.1721	0.0183	0.9849	-0.9022	0.2229	0.1535	-0.0799	0.2910	-0.8923
	Side-to-Side	-0.1559	0.3438	0.9260	-0.1289	-0.9915	-0.0164	0.9125	-0.1219	0.1989	0.1813	0.8760	-0.2947
**L18**	End-to-End	-0.2706	-0.8366	-0.4763	-0.7262	0.2794	0.6281	-0.3924	0.5159	-0.6832	0.8173	0.0021	-0.4679
	Side-to-Side	-0.2227	0.0059	0.9749	0.5372	-0.8152	-0.2166	0.7934	0.4754	0.1784	-0.4624	0.8132	-0.1106
**L20**	End-to-End	0.1998	-0.9739	-0.1074	0.3031	0.3076	0.9020	-0.8454	-0.2127	0.3566	-0.3702	0.0196	-0.8659
	Side-to-Side	0.7033	0.7039	0.0995	-0.3468	-0.2585	0.9016	0.6604	-0.6686	0.0623	0.1104	0.0219	-0.9351
**L22**	End-to-End	0.2319	-0.9426	-0.2404	-0.5170	0.0111	0.8559	-0.8041	-0.0742	-0.4847	0.4390	0.3057	-0.7751
**S6**	End-to-End	-0.4277	0.6642	0.6131	0.9795	0.1891	-0.0700	-0.1624	0.5706	-0.7314	-0.8356	-0.4124	-0.1362
**S8**	End-to-End	-0.9390	-0.2167	0.2672	0.0504	0.1079	-0.9929	0.1864	-0.9188	-0.0904	0.2651	-0.0351	0.9031
	Side-to-Side	-0.7297	0.0275	-0.6832	0.3338	0.9267	0.1728	0.6379	-0.1020	-0.6854	-0.0885	-0.9359	0.0568
**S11**	End-to-End	0.6847	-0.6670	0.2936	-0.7021	-0.4567	-0.5464	0.4985	0.1680	-0.7811	0.4717	0.6812	0.4476
	Side-to-Side	-0.7559	0.6459	0.1068	0.1440	-0.4941	0.8574	0.6065	0.6635	0.2805	0.1103	0.2768	-0.8933
**L10+L11**	End-to-End	-0.4355	0.3799	-0.8161	0.6952	-0.6664	-0.2697	-0.6462	-0.6848	0.0262	-0.5489	0.5388	0.5437
	Side-to-Side	0.4540	0.7685	0.4508	0.4036	-0.8596	0.3134	0.6283	0.0397	-0.7005	-0.5562	0.6013	-0.4649
**L15+L18**	End-to-End	-0.6912	-0.7121	-0.1232	-0.4423	0.8525	0.2787	-0.0935	0.2471	-0.9042	0.6743	-0.6134	-0.2374
	Side-to-Side	-0.9330	0.2424	0.2660	0.0203	-0.9426	-0.3332	0.1700	-0.3055	0.8746	0.2933	0.8612	0.2438
**L9+S6**	End-to-End	0.4690	0.6196	0.6294	0.3653	-0.9250	0.1041	0.6467	0.1811	-0.6602	-0.5230	0.7167	-0.3158
	Side-to-Side	-0.7079	0.3634	-0.6057	-0.1951	0.2684	0.9433	0.5054	0.7859	-0.1191	0.4327	-0.3904	-0.74
**S6+S11**	End-to-End	-0.6545	0.7561	0.0013	0.8085	0.2540	0.5309	0.4011	0.3485	-0.7775	-0.5883	-0.5083	-0.5313
	Side-to-Side	-0.7152	0.6942	0.0812	0.1957	-0.3881	0.9006	0.6567	0.6600	0.1417	0.0448	0.1547	-0.9279
**L9+S11**	End-to-End	-0.5967	-0.8024	-0.0124	-0.1224	0.4962	0.8595	-0.6835	0.5144	-0.3943	0.3228	-0.2268	-0.8554
	Side-to-Side	-0.7586	0.4376	0.4828	0.3187	-0.7911	0.5222	0.6104	0.5499	0.4607	-0.0639	0.6441	-0.6843
**L9+S6+S11**	End-to-End	-0.6130	-0.7898	0.0210	-0.0668	0.5009	0.8629	-0.6921	0.5276	-0.3598	0.2731	-0.2350	-0.8700
	Side-to-Side	-0.7692	0.4894	0.4108	0.5655	-0.4025	0.7199	0.5177	0.7861	0.0328	-0.3068	0.2379	-0.8580
	Side-to-Side2	-0.3640	0.5519	-0.7503	-0.2690	0.4038	0.8744	0.7856	0.5201	0.0015	0.3910	-0.5888	-0.6229

Table S6. Orthogonal axes originating at the C2 atom of substrates in Q-Chem coordinates.

RPs	Possible Binding Modes	DHAP1											
		a''_x	a''_y	a''_z	b''_x	b''_y	b''_z	c''_x	c''_y	c''_z	d''_x	d''_y	d''_z
**L9**	End-to-End	0.9132	0.0996	-0.3951	-0.5098	0.8472	0.1495	0.3497	0.3497	0.8244	0.1077	-0.8910	0.0244
	Side-to-Side	0.9130	0.1002	-0.3954	-0.5097	0.8472	0.1497	0.3500	0.0649	0.8246	0.1083	-0.8913	0.0241
**L10**	End-to-End	0.9132	0.1003	-0.3950	-0.5097	0.8473	0.1494	0.3497	0.0649	0.8249	0.1083	-0.8914	0.0242
	Side-to-Side	0.9130	0.0999	-0.3956	-0.5100	0.8472	0.1489	0.3500	0.0658	0.8244	0.1084	-0.8911	0.0251
**L11**	End-to-End	0.9134	0.0999	-0.3945	-0.5100	0.8470	0.1501	0.3492	0.0641	0.8246	0.1077	-0.8910	0.0237
	Side-to-Side	0.9132	0.0998	-0.3951	-0.5098	0.8472	0.1492	0.3497	0.0652	0.8246	0.1080	-0.8911	0.0246
**L15**	End-to-End	0.9132	0.1000	-0.3950	-0.5101	0.8471	0.1494	0.3495	0.0650	0.8246	0.1082	-0.8911	0.0244
	Side-to-Side	0.9131	0.0994	-0.3954	-0.5099	0.8472	0.1491	0.3498	0.0655	0.8243	0.1078	-0.8910	0.0250
**L18**	End-to-End	0.9131	0.0997	-0.3954	-0.5098	0.8473	0.1491	0.3499	0.0655	0.8245	0.1081	-0.8912	0.0249
	Side-to-Side	0.9132	0.0994	-0.3951	-0.5095	0.8473	0.1498	0.3497	0.0645	0.8245	0.1074	-0.8911	0.0242
**L20**	End-to-End	0.9134	0.0997	-0.3948	-0.5101	0.8470	0.1496	0.3493	0.0647	0.8244	0.1077	-0.8909	0.0243
	Side-to-Side	0.9132	0.0995	-0.3952	-0.5104	0.8470	0.1488	0.3496	0.0658	0.8242	0.1080	-0.8908	0.0253
**L22**	End-to-End	0.9130	0.0995	-0.3955	-0.5102	0.8470	0.1490	0.3499	0.0658	0.8241	0.1080	-0.8909	0.0252
**S6**	End-to-End	0.9131	0.1000	-0.3952	-0.5098	0.8473	0.1491	0.3498	0.0653	0.8246	0.1082	-0.8912	0.0246
**S8**	End-to-End	0.9132	0.0998	-0.3951	-0.5098	0.8472	0.1495	0.3496	0.0649	0.8246	0.1080	-0.8912	0.0244
	Side-to-Side	0.9131	0.1002	-0.3953	-0.5098	0.8472	0.1493	0.3499	0.0652	0.8247	0.1085	-0.8913	0.0245
**S11**	End-to-End	0.9133	0.0999	-0.3948	-0.5098	0.8472	0.1496	0.3494	0.0646	0.8247	0.1079	-0.8911	0.0241
	Side-to-Side	0.9132	0.0999	-0.3950	-0.5097	0.8473	0.1495	0.3496	0.0649	0.8247	0.1080	-0.8912	0.0243
**L10+L11**	End-to-End	0.9132	0.0996	-0.3951	-0.5100	0.8472	0.1491	0.3496	0.0653	0.8244	0.1079	-0.8910	0.0248
	Side-to-Side	0.9133	0.0997	-0.3949	-0.5099	0.8471	0.1499	0.3495	0.0645	0.8245	0.1077	-0.8910	0.0240
**L15+L18**	End-to-End	0.9131	0.1001	-0.3953	-0.5098	0.8473	0.1492	0.3498	0.0653	0.8247	0.1083	-0.8913	0.0246
	Side-to-Side	0.9133	0.0995	-0.3951	-0.5095	0.8474	0.1495	0.3496	0.0648	0.8245	0.1076	-0.8911	0.0244
**L9+S6**	End-to-End	0.9133	0.0990	-0.3950	-0.5102	0.8470	0.1494	0.3494	0.0650	0.8241	0.1073	-0.8907	0.0248
	Side-to-Side	0.9132	0.0999	-0.3952	-0.5097	0.8472	0.1498	0.3498	0.0646	0.8245	0.1079	-0.8912	0.0240
**S6+S11**	End-to-End	0.9133	0.0990	-0.3952	-0.5101	0.8470	0.1494	0.3495	0.0652	0.8241	0.1074	-0.8907	0.0249
	Side-to-Side	0.9133	0.0996	-0.3949	-0.5097	0.8472	0.1498	0.3495	0.0645	0.8245	0.1076	-0.8911	0.0241
**L9+S11**	End-to-End	0.9133	0.0995	-0.3950	-0.5101	0.8470	0.1495	0.3495	0.0649	0.8243	0.1077	-0.8909	0.0245
	Side-to-Side	0.9132	0.1002	-0.3951	-0.5097	0.8472	0.1497	0.3497	0.0647	0.8247	0.1082	-0.8913	0.0240
**L9+S6+S11**	End-to-End	0.9130	0.0995	-0.3955	-0.5101	0.8470	0.1498	0.3499	0.0650	0.8241	0.1078	-0.8908	0.0245
	Side-to-Side	0.9133	0.0996	-0.3950	-0.5099	0.8472	0.1496	0.3495	0.0647	0.8245	0.1077	-0.8910	0.0243
	Side-to-Side2	0.9133	0.0998	-0.3948	-0.5103	0.8468	0.1498	0.3493	0.0647	0.8244	0.1078	-0.8908	0.0242

Continued page of Table S6

**RPs**	**Possible Binding Modes**	**DHAP2**											
		**a”**$_x$	**a”**$_y$	**a”**$_z$	**b”**$_x$	**b”**$_y$	**b”**$_z$	**c”**$_x$	**c”**$_y$	**c”**$_z$	**d”**$_x$	**d”**$_y$	**d”**$_z$
**L9**	End-to-End	0.9080	0.3361	-0.2502	-0.6955	0.6991	0.1658	0.2306	0.0235	0.8685	0.2977	-0.8463	-0.0562
	Side-to-Side	0.9079	0.3364	-0.2502	-0.6953	0.6993	0.1659	0.2308	0.0233	0.8688	0.2981	-0.8465	-0.0565
**L10**	End-to-End	0.9081	0.3356	-0.2502	-0.6956	0.6990	0.1661	0.2307	0.0232	0.8682	0.2972	-0.8462	-0.0563
	Side-to-Side	0.9080	0.3362	-0.2500	-0.6954	0.6992	0.1662	0.2307	0.0229	0.8686	0.2978	-0.8464	-0.0568
**L11**	End-to-End	0.9081	0.3364	-0.2495	-0.6949	0.6995	0.1665	0.2305	0.0222	0.8690	0.2979	-0.8466	-0.0574
	Side-to-Side	0.9081	0.3359	-0.2501	-0.6955	0.6990	0.1661	0.2306	0.0232	0.8684	0.2975	-0.8463	-0.0564
**L15**	End-to-End	0.9079	0.3363	-0.2503	-0.6956	0.6989	0.1663	0.2308	0.0231	0.8685	0.2979	-0.8462	-0.0567
	Side-to-Side	0.9081	0.3362	-0.2494	-0.6952	0.6993	0.1663	0.2303	0.0224	0.8688	0.2977	-0.8465	-0.0571
**L18**	End-to-End	0.9081	0.3364	-0.2494	-0.6951	0.6994	0.1662	0.2303	0.0224	0.8689	0.2979	-0.8465	-0.0571
	Side-to-Side	0.9080	0.3360	-0.2502	-0.6955	0.6991	0.1662	0.2307	0.0231	0.8684	0.2976	-0.8463	-0.0565
**L20**	End-to-End	0.9081	0.3360	-0.2499	-0.6956	0.6990	0.1660	0.2304	0.0231	0.8685	0.2976	-0.8463	-0.0565
	Side-to-Side	0.9081	0.3360	-0.2500	-0.6953	0.6992	0.1664	0.2307	0.0227	0.8685	0.2975	-0.8464	-0.0569
**L22**	End-to-End	0.9080	0.3362	-0.2499	-0.6954	0.6991	0.1660	0.2306	0.0231	0.8686	0.2978	-0.8464	-0.0566
**S6**	End-to-End	0.9081	0.3361	-0.2498	-0.6959	0.6987	0.1663	0.2304	0.0228	0.8683	0.2976	-0.8461	-0.0567
**S8**	End-to-End	0.9082	0.3356	-0.2500	-0.6953	0.6993	0.1659	0.2305	0.0231	0.8685	0.2973	-0.8464	-0.0564
	Side-to-Side	0.9081	0.3360	-0.2500	-0.6952	0.6994	0.1660	0.2306	0.0230	0.8687	0.2976	-0.8465	-0.0566
**S11**	End-to-End	0.9081	0.3360	-0.2500	-0.6953	0.6993	0.1659	0.2306	0.0231	0.8686	0.2976	-0.8464	-0.0564
	Side-to-Side	0.9083	0.3357	-0.2496	-0.6952	0.6993	0.1663	0.2304	0.0225	0.8686	0.2972	-0.8464	-0.0570
**L10+L11**	End-to-End	0.9080	0.3361	-0.2504	-0.6954	0.6992	0.1660	0.2308	0.0234	0.8685	0.2978	-0.8464	-0.0563
	Side-to-Side	0.9082	0.3361	-0.2495	-0.6957	0.6988	0.1662	0.2302	0.0227	0.8685	0.2976	-0.8462	-0.0568
**L15+L18**	End-to-End	0.9080	0.3360	-0.2501	-0.6954	0.6993	0.1657	0.2306	0.0235	0.8686	0.2977	-0.8464	-0.0562
	Side-to-Side	0.9078	0.3368	-0.2498	-0.6953	0.6993	0.1662	0.2307	0.0228	0.8690	0.2983	-0.8465	-0.0569
**L9+S6**	End-to-End	0.9082	0.3360	-0.2496	-0.6955	0.6990	0.1662	0.2303	0.0226	0.8685	0.2975	-0.8463	-0.0568
	Side-to-Side	0.9079	0.3368	-0.2497	-0.6954	0.6992	0.1660	0.2305	0.0229	0.8689	0.2983	-0.8465	-0.0568
**S6+S11**	End-to-End	0.9083	0.3357	-0.2498	-0.6955	0.6991	0.1659	0.2303	0.0230	0.8684	0.2973	-0.8463	-0.0564
	Side-to-Side	0.9082	0.3358	-0.2498	-0.6954	0.6993	0.1657	0.2303	0.0232	0.8686	0.2975	-0.8464	-0.0563
**L9+S11**	End-to-End	0.9081	0.3362	-0.2498	-0.6953	0.6992	0.1660	0.2305	0.0229	0.8687	0.2978	-0.8464	-0.0567
	Side-to-Side	0.9081	0.3364	-0.2495	-0.6957	0.6988	0.1666	0.2304	0.0223	0.8685	0.2977	-0.8462	-0.0573
**L9+S6+S11**	End-to-End	0.9080	0.3361	-0.2502	-0.6957	0.6989	0.1661	0.2307	0.0232	0.8684	0.2977	-0.8462	-0.0565
	Side-to-Side	0.9082	0.3358	-0.2497	-0.6956	0.6989	0.1665	0.2305	0.0225	0.8683	0.2972	-0.8462	-0.0569
	Side-to-Side2	0.9081	0.3361	-0.2497	-0.6956	0.6991	0.1658	0.2303	0.0232	0.8686	0.2977	-0.8463	-0.0564

Continued page of Table S6

RPs	Possible Binding Modes	GAP1											
		**a”$_x$**	**a”$_y$**	**a”$_z$**	**b”$_x$**	**b”$_y$**	**b”$_z$**	**c”$_x$**	**c”$_y$**	**c”$_z$**	**d”$_x$**	**d”$_y$**	**d”$_z$**
**L9**	End-to-End	0.8638	0.1655	-0.4759	-0.7854	0.5268	-0.3250	0.1969	0.6545	0.5850	0.4083	-0.5991	0.5327
	Side-to-Side	0.8640	0.1651	-0.4756	-0.7858	0.5265	-0.3246	0.1968	0.6542	0.5846	0.4077	-0.5987	0.5328
**L10**	End-to-End	0.8639	0.1658	-0.4755	-0.7857	0.5268	-0.3243	0.1967	0.6538	0.5854	0.4080	-0.5993	0.5322
	Side-to-Side	0.8636	0.1661	-0.4761	-0.7855	0.5268	-0.3248	0.1969	0.6545	0.5854	0.4088	-0.5992	0.5325
**L11**	End-to-End	0.8639	0.1658	-0.4755	-0.7854	0.5272	-0.3245	0.1968	0.6538	0.5857	0.4080	-0.5996	0.5322
	Side-to-Side	0.8639	0.1657	-0.4756	-0.7853	0.5271	-0.3248	0.1969	0.6541	0.5855	0.4081	-0.5994	0.5325
**L15**	End-to-End	0.8637	0.1656	-0.4760	-0.7854	0.5270	-0.3248	0.1971	0.6544	0.5852	0.4084	-0.5992	0.5326
	Side-to-Side	0.8639	0.1655	-0.4756	-0.7854	0.5268	-0.3250	0.1968	0.6544	0.5851	0.4081	-0.5991	0.5328
**L18**	End-to-End	0.8638	0.1658	-0.4758	-0.7854	0.5268	-0.3250	0.1967	0.6544	0.5853	0.4084	-0.5992	0.5327
	Side-to-Side	0.8637	0.1658	-0.4759	-0.7856	0.5265	-0.3250	0.1967	0.6546	0.5850	0.4085	-0.5989	0.5328
**L20**	End-to-End	0.8640	0.1658	-0.4754	-0.7854	0.5272	-0.3243	0.1969	0.6536	0.5857	0.4078	-0.5997	0.5321
	Side-to-Side	0.8637	0.1656	-0.4761	-0.7855	0.5265	-0.3251	0.1968	0.6548	0.5848	0.4086	-0.5988	0.5329
**L22**	End-to-End	0.8637	0.1654	-0.4761	-0.7857	0.5265	-0.3248	0.1970	0.6546	0.5847	0.4083	-0.5988	0.5328
**S6**	End-to-End	0.8639	0.1650	-0.4759	-0.7854	0.5267	-0.3251	0.1970	0.6546	0.5846	0.4080	-0.5988	0.5330
**S8**	End-to-End	0.8637	0.1656	-0.4761	-0.7855	0.5269	-0.3246	0.1971	0.6543	0.5851	0.4084	-0.5992	0.5325
	Side-to-Side	0.8639	0.1657	-0.4756	-0.7854	0.5271	-0.3245	0.1970	0.6539	0.5856	0.4080	-0.5995	0.5322
**S11**	End-to-End	0.8637	0.1655	-0.4761	-0.7857	0.5267	-0.3245	0.1970	0.6543	0.5849	0.4084	-0.5990	0.5325
	Side-to-Side	0.8639	0.1652	-0.4757	-0.7857	0.5265	-0.3248	0.1968	0.6544	0.5847	0.4079	-0.5987	0.5328
**L10+L11**	End-to-End	0.8636	0.1662	-0.4760	-0.7854	0.5269	-0.3248	0.1969	0.6544	0.5855	0.4088	-0.5994	0.5324
	Side-to-Side	0.8637	0.1659	-0.4760	-0.7851	0.5272	-0.3250	0.1970	0.6544	0.5856	0.4087	-0.5995	0.5325
**L15+L18**	End-to-End	0.8636	0.1657	-0.4762	-0.7857	0.5267	-0.3246	0.1970	0.6544	0.5850	0.4086	-0.5990	0.5325
	Side-to-Side	0.8639	0.1658	-0.4756	-0.7854	0.5271	-0.3245	0.1969	0.6539	0.5856	0.4081	-0.5995	0.5322
**L9+S6**	End-to-End	0.8638	0.1661	-0.4758	-0.7855	0.5270	-0.3246	0.1968	0.6540	0.5856	0.4084	-0.5995	0.5322
	Side-to-Side	0.8636	0.1663	-0.4759	-0.7852	0.5272	-0.3249	0.1969	0.6543	0.5859	0.4088	-0.5997	0.5323
**S6+S11**	End-to-End	0.8639	0.1652	-0.4759	-0.7854	0.5269	-0.3248	0.1971	0.6544	0.5849	0.4080	-0.5990	0.5327
	Side-to-Side	0.8637	0.1661	-0.4758	-0.7854	0.5268	-0.3251	0.1967	0.6544	0.5855	0.4086	-0.5993	0.5326
**L9+S11**	End-to-End	0.8641	0.1655	-0.4753	-0.7856	0.5266	-0.3247	0.1966	0.6540	0.5851	0.4077	-0.5990	0.5326
	Side-to-Side	0.8638	0.1656	-0.4758	-0.7856	0.5268	-0.3245	0.1969	0.6542	0.5851	0.4082	-0.5992	0.5325
**L9+S6+S11**	End-to-End	0.8637	0.1650	-0.4763	-0.7856	0.5264	-0.3251	0.1971	0.6549	0.5843	0.4084	-0.5985	0.5331
	Side-to-Side	0.8636	0.1658	-0.4761	-0.7856	0.5266	-0.3248	0.1969	0.6545	0.5851	0.4086	-0.5990	0.5326
	Side-to-Side2	0.8640	0.1653	-0.4756	-0.7856	0.5266	-0.3247	0.1968	0.6542	0.5848	0.4078	-0.5989	0.5327

Continued page of Table S6

RPs	Possible Binding Modes	GAP2											
		**a”$_x$**	**a”$_y$**	**a”$_z$**	**b”$_x$**	**b”$_y$**	**b”$_z$**	**c”$_x$**	**c”$_y$**	**c”$_z$**	**d”$_x$**	**d”$_y$**	**d”$_z$**
**L9**	End-to-End	-0.9443	0.2466	-0.2179	0.2340	-0.8624	-0.4489	-0.2986	-0.4749	0.7567	0.0831	0.7796	0.5221
	Side-to-Side	-0.9442	0.2473	-0.2174	0.2340	-0.8622	-0.4493	-0.2986	-0.4752	0.7562	0.0837	0.7789	0.5225
**L10**	End-to-End	-0.9443	0.2467	-0.2176	0.2344	-0.8623	-0.4489	-0.2984	-0.4750	0.7564	0.0833	0.7793	0.5221
	Side-to-Side	-0.9445	0.2464	-0.2172	0.2336	-0.8625	-0.4489	-0.2980	-0.4748	0.7571	0.0834	0.7798	0.5218
**L11**	End-to-End	-0.9444	0.2465	-0.2177	0.2344	-0.8622	-0.4490	-0.2984	-0.4751	0.7565	0.0830	0.7794	0.5222
	Side-to-Side	-0.9443	0.2469	-0.2178	0.2334	-0.8620	-0.4500	-0.2988	-0.4758	0.7563	0.0831	0.7792	0.5230
**L15**	End-to-End	-0.9444	0.2464	-0.2179	0.2341	-0.8624	-0.4489	-0.2985	-0.4749	0.7567	0.0830	0.7796	0.5221
	Side-to-Side	-0.9444	0.2466	-0.2177	0.2342	-0.8622	-0.4493	-0.2984	-0.4752	0.7564	0.0831	0.7793	0.5224
**L18**	End-to-End	-0.9444	0.2468	-0.2174	0.2344	-0.8621	-0.4493	-0.2983	-0.4753	0.7563	0.0833	0.7791	0.5225
	Side-to-Side	-0.9445	0.2465	-0.2174	0.2336	-0.8624	-0.4490	-0.2981	-0.4749	0.7570	0.0833	0.7797	0.5220
**L20**	End-to-End	-0.9442	0.2469	-0.2180	0.2340	-0.8622	-0.4493	-0.2989	-0.4752	0.7563	0.0832	0.7792	0.5225
	Side-to-Side	-0.9442	0.2469	-0.2178	0.2342	-0.8621	-0.4495	-0.2987	-0.4754	0.7562	0.0831	0.7791	0.5227
**L22**	End-to-End	-0.9441	0.2472	-0.2179	0.2340	-0.8621	-0.4495	-0.2990	-0.4754	0.7561	0.0833	0.7790	0.5227
**S6**	End-to-End	-0.9441	0.2472	-0.2179	0.2341	-0.8621	-0.4495	-0.2990	-0.4754	0.7560	0.0834	0.7789	0.5227
**S8**	End-to-End	-0.9441	0.2470	-0.2183	0.2343	-0.8619	-0.4497	-0.2992	-0.4757	0.7559	0.0829	0.7790	0.5230
	Side-to-Side	-0.9443	0.2471	-0.2172	0.2336	-0.8623	-0.4493	-0.2983	-0.4750	0.7566	0.0838	0.7793	0.5223
**S11**	End-to-End	-0.9442	0.2469	-0.2180	0.2346	-0.8622	-0.4490	-0.2988	-0.4751	0.7562	0.0831	0.7791	0.5223
	Side-to-Side	-0.9444	0.2471	-0.2171	0.2339	-0.8623	-0.4491	-0.2982	-0.4749	0.7565	0.0838	0.7792	0.5222
**L10+L11**	End-to-End	-0.9443	0.2469	-0.2177	0.2338	-0.8623	-0.4491	-0.2986	-0.4750	0.7566	0.0834	0.7794	0.5223
	Side-to-Side	-0.9443	0.2469	-0.2174	0.2338	-0.8624	-0.4491	-0.2983	-0.4749	0.7566	0.0836	0.7794	0.5221
**L15+L18**	End-to-End	-0.9443	0.2468	-0.2177	0.2339	-0.8620	-0.4496	-0.2987	-0.4755	0.7563	0.0831	0.7792	0.5227
	Side-to-Side	-0.9441	0.2473	-0.2178	0.2338	-0.8622	-0.4494	-0.2989	-0.4752	0.7562	0.0835	0.7791	0.5226
**L9+S6**	End-to-End	-0.9443	0.2470	-0.2173	0.2341	-0.8621	-0.4493	-0.2983	-0.4752	0.7563	0.0835	0.7791	0.5224
	Side-to-Side	-0.9443	0.2473	-0.2173	0.2333	-0.8622	-0.4496	-0.2986	-0.4752	0.7565	0.0838	0.7792	0.5226
**S6+S11**	End-to-End	-0.9442	0.2472	-0.2176	0.2341	-0.8623	-0.4491	-0.2986	-0.4750	0.7563	0.0836	0.7791	0.5223
	Side-to-Side	-0.9442	0.2472	-0.2177	0.2342	-0.8618	-0.4499	-0.2988	-0.4758	0.7558	0.0833	0.7787	0.5231
**L9+S11**	End-to-End	-0.9442	0.2472	-0.2178	0.2338	-0.8620	-0.4497	-0.2989	-0.4756	0.7561	0.0833	0.7790	0.5229
	Side-to-Side	-0.9444	0.2465	-0.2173	0.2339	-0.8622	-0.4493	-0.2982	-0.4752	0.7567	0.0833	0.7794	0.5223
**L9+S6+S11**	End-to-End	-0.9443	0.2471	-0.2172	0.2342	-0.8620	-0.4496	-0.2984	-0.4754	0.7561	0.0836	0.7788	0.5227
	Side-to-Side	-0.9441	0.2473	-0.2180	0.2342	-0.8618	-0.4499	-0.2991	-0.4758	0.7558	0.0832	0.7787	0.5231
	Side-to-Side2	-0.9443	0.2467	-0.2179	0.2335	-0.8622	-0.4496	-0.2987	-0.4754	0.7565	0.0830	0.7795	0.5226

Table S7. Database inputs for pLink.

RPs	MW (kDa)	Searchable? (Y/N) [a]	Accessible? (Y/N) [b]
L7/L12 [c]	12.3	Y	Y
**L9**	**15.8**	**Y**	**Y**
**L10**	**17.7**	**Y**	**Y**
**L11**	**14.9**	**Y**	**Y**
L13	16	N	N/A
L14	13.5	N	N/A
**L15**	**15**	**Y**	**Y**
L16	15.3	Y	N
L17	14.4	Y	N
**L18**	**12.8**	**Y**	**Y**
L19	13.1	N	N/A
**L20**	**13.5**	**Y**	**Y**
**L22**	**12.2**	**Y**	**Y**
S5	12.3	Y	N
**S6**	**15.8**	**Y**	**Y**
**S8**	**17.7**	**Y**	**Y**
S9	14.9	Y	N
**S11**	**16**	**Y**	**Y**
S12	13.5	Y	N
S13	15	Y	N

[a] Intermolecular crosslinks were searchable through pLink2.0 software. (Y = Yes, N = No).
[b] Solvent accessibility of crosslinked residues was over 40% in the starting PDB models 5UYK of intact ribosome and 8TIM of TPI. (Y = Yes, N = No, N/A data unavailable).
[c] L7/L12 was eliminated from the plink2.0 outputs due to no obvious changes in the ^{1}H-^{15}N HSQC spectra of [U-^{15}N] intact ribosomes upon His-TPI titration. (Figure S1).
Bold RPs were used in subsequent analyses.

Table S8. Substrate dipole moments.

RPs	Possible Binding Modes	DHAP1					
		μ_x" (D) [a]	μ_y" (D) [a]	μ_z" (D) [a]	μ_x (D) [b]	μ_y (D) [b]	μ_z (D) [b]
**L9**	End-to-End	-19.0880	0.9527	-3.9369	13.3518	-13.7026	3.8375
	Side-to-Side	-19.0901	0.9563	-3.9270	-9.8026	-13.9812	9.4447
**L10**	End-to-End	-19.0908	0.9540	-3.9315	0.4725	-10.2914	-16.5737
	Side-to-Side	-19.0886	0.9548	-3.9360	14.8468	11.7068	4.8270
**L11**	End-to-End	-19.1017	0.9580	-3.9243	-19.2016	3.3123	1.2319
	Side-to-Side	-19.0997	0.9507	-3.9289	-16.4600	-1.6418	10.3688
**L15**	End-to-End	-19.0938	0.9522	-3.9237	-12.4492	-14.7176	-3.0469
	Side-to-Side	-19.0925	0.9474	-3.9300	4.7883	4.5039	18.3754
**L18**	End-to-End	-19.0961	0.9454	-3.9336	-8.6719	-10.5602	-13.9394
	Side-to-Side	-19.0962	0.9481	-3.9314	3.0605	3.5509	18.9484
**L20**	End-to-End	-19.0916	0.9636	-3.9282	-3.3402	-19.1547	1.6700
	Side-to-Side	-19.0832	0.9502	-3.9324	18.0269	6.8634	2.9089
**L22**	End-to-End	-19.0861	0.9558	-3.9263	-2.9512	-17.6038	-7.8744
**S6**	End-to-End	-19.0913	0.9474	-3.9307	-8.4555	16.9135	4.8233
**S8**	End-to-End	-19.0884	0.9574	-3.9324	-15.3106	-11.6075	3.4058
	Side-to-Side	-19.1037	0.9490	-3.9333	-7.5589	0.2389	-18.0035
**S11**	End-to-End	-19.0919	0.9480	-3.9346	16.1127	-10.8860	-1.6600
	Side-to-Side	-19.0848	0.9468	-3.9380	-8.4069	16.9176	4.8737
**L10+L11**	End-to-End	-19.0849	0.9636	-3.9307	-12.8499	0.6286	-14.6662
	Side-to-Side	-19.0915	0.9596	-3.9304	13.7511	13.6353	2.4165
**L15+L18**	End-to-End	-19.0921	0.9601	-3.9310	-13.5297	-10.2057	-9.6784
	Side-to-Side	-19.0918	0.9481	-3.9320	-15.3678	1.1841	11.9701
**L9+S6**	End-to-End	-19.0888	0.9582	-3.9331	14.1535	12.0960	5.8426
	Side-to-Side	-19.0817	0.9541	-3.9358	-8.6206	13.3769	-11.2807
**S6+S11**	End-to-End	-19.0787	0.9500	-3.9335	-7.8822	16.7276	-6.1993
	Side-to-Side	-19.0798	0.9490	-3.9301	-7.2082	17.8325	3.2295
**L9+S11**	End-to-End	-19.0824	0.9526	-3.9341	-16.6078	-9.7871	-2.9861
	Side-to-Side	-19.0890	0.9462	-3.9242	-8.3456	12.0042	12.9204
**L9+S6+S11**	End-to-End	-19.0929	0.9536	-3.9370	-16.9450	-9.4632	-2.0646
	Side-to-Side	-19.0897	0.9595	-3.9376	-9.1144	15.2113	8.1476
	Side-to-Side2	-19.0952	0.9528	-3.9404	-0.0995	14.6025	-12.9545

Continued page of Table S8

RPs	Possible Binding Modes	DHAP2					
		μ_x'' (D) [a]	μ_y'' (D) [a]	μ_z'' (D) [a]	μ_x (D) [b]	μ_y (D) [b]	μ_z (D) [b]
**L9**	End-to-End	-21.1571	2.8783	-4.0745	-13.5460	15.8681	-6.1005
	Side-to-Side	-21.1475	2.8769	-4.0616	10.8866	14.1617	-12.3660
**L10**	End-to-End	-21.1533	2.8736	-4.0766	-1.1819	9.4843	19.5189
	Side-to-Side	-21.1441	2.8748	-4.0642	-15.0663	-14.0678	-6.8525
**L11**	End-to-End	-21.1509	2.8782	-4.0682	21.6107	-2.2324	0.4314
	Side-to-Side	-21.1463	2.8820	-4.0651	16.9628	2.0593	-13.4170
**L15**	End-to-End	-21.1463	2.8818	-4.0732	13.7087	16.8200	1.1045
	Side-to-Side	-21.1489	2.8778	-4.0660	-5.0302	-2.7577	-20.9567
**L18**	End-to-End	-21.1487	2.8827	-4.0675	11.6377	11.4682	14.3237
	Side-to-Side	-21.1386	2.8774	-4.0647	-4.6917	-2.0043	-21.1096
**L20**	End-to-End	-21.1380	2.8891	-4.0700	2.9799	21.1296	-4.0477
	Side-to-Side	-21.1461	2.8765	-4.0731	-19.6953	-7.3231	-5.5217
**L22**	End-to-End	-21.1410	2.8754	-4.0733	4.5268	20.1045	6.8645
**S6**	End-to-End	-21.1567	2.8684	-4.0756	7.2589	-19.7474	-5.4581
**S8**	End-to-End	-21.1445	2.8723	-4.0763	17.4252	12.8876	-1.4931
	Side-to-Side	-21.1377	2.8725	-4.0659	7.9200	-2.5471	20.0592
**S11**	End-to-End	-21.1491	2.8749	-4.0771	-16.6544	13.6030	3.1244
	Side-to-Side	-21.1368	2.8874	-4.0744	9.3531	-18.0568	-7.6270
**L10+L11**	End-to-End	-21.1396	2.8808	-4.0719	12.9305	0.8027	17.4333
	Side-to-Side	-21.1548	2.8737	-4.0762	-16.6367	-13.5075	-3.6289
**L15+L18**	End-to-End	-21.1487	2.8715	-4.0726	16.5466	9.6402	10.2653
	Side-to-Side	-21.1365	2.8702	-4.0756	17.5544	0.9110	-12.7520
**L9+S6**	End-to-End	-21.1436	2.8765	-4.0682	-16.9886	-11.5678	-7.0320
	Side-to-Side	-21.1518	2.8709	-4.0760	10.4291	-15.8500	10.5957
**S6+S11**	End-to-End	-21.1532	2.8723	-4.0724	7.1188	-19.7290	5.6907
	Side-to-Side	-21.1415	2.8866	-4.0621	7.8615	-19.3808	-5.8631
**L9+S11**	End-to-End	-21.1374	2.8840	-4.0663	19.2036	10.0619	1.2738
	Side-to-Side	-21.1406	2.8712	-4.0672	8.8531	-11.7242	-15.9962
**L9+S6+S11**	End-to-End	-21.1405	2.8823	-4.0698	19.4445	9.6777	0.2120
	Side-to-Side	-21.1508	2.8792	-4.0720	9.1348	-16.3174	-11.0692
	Side-to-Side2	-21.1421	2.8747	-4.0804	0.8880	-17.6162	12.6801

Continued page of Table S8

RPs	Possible Binding Modes	GAP1					
		μ_x'' (D) [a]	μ_y'' (D) [a]	μ_z'' (D) [a]	μ_x (D) [b]	μ_y (D) [b]	μ_z (D) [b]
**L9**	End-to-End	-20.4749	-1.2882	-4.0304	15.8488	-13.5780	1.2561
	Side-to-Side	-20.4857	-1.2848	-4.0295	-10.4788	-16.6468	7.1156
**L10**	End-to-End	-20.4922	-1.2764	-4.0190	-0.2988	-13.7137	-15.7973
	Side-to-Side	-20.4816	-1.2771	-4.0292	17.7514	10.6410	3.0032
**L11**	End-to-End	-20.4837	-1.2808	-4.0231	-19.7765	5.4527	4.0699
	Side-to-Side	-20.4755	-1.2713	-4.0220	-19.2226	-1.1911	8.1309
**L15**	End-to-End	-20.4782	-1.2799	-4.0233	-13.4233	-14.6510	-6.5073
	Side-to-Side	-20.4823	-1.2853	-4.0290	5.6531	8.0294	18.4656
**L18**	End-to-End	-20.4740	-1.2823	-4.0265	-6.1989	-11.3082	-16.4542
	Side-to-Side	-20.4881	-1.2847	-4.0246	1.4210	6.7101	19.7627
**L20**	End-to-End	-20.4812	-1.2737	-4.0262	-4.7830	-20.3077	-1.4255
	Side-to-Side	-20.4777	-1.2854	-4.0230	19.5123	7.5062	-0.3166
**L22**	End-to-End	-20.4770	-1.2725	-4.0325	-1.4413	-17.6417	-11.1303
**S6**	End-to-End	-20.4706	-1.2782	-4.0328	-12.0059	16.4403	4.7453
**S8**	End-to-End	-20.4750	-1.2870	-4.0264	-15.3739	-12.3546	6.9355
	Side-to-Side	-20.4747	-1.2714	-4.0343	-8.4581	-3.1176	-18.8639
**S11**	End-to-End	-20.4894	-1.2682	-4.0308	18.8027	-9.1720	-0.0360
	Side-to-Side	-20.4758	-1.2777	-4.0211	-8.6606	18.9334	1.8928
**L10+L11**	End-to-End	-20.4709	-1.2763	-4.0255	-15.5908	2.7290	-13.6518
	Side-to-Side	-20.4879	-1.2740	-4.0208	12.5471	16.7065	1.0035
**L15+L18**	End-to-End	-20.4792	-1.2736	-4.0307	-11.9588	-13.1300	-11.0388
	Side-to-Side	-20.4733	-1.2775	-4.0241	-15.3319	4.4236	13.5036
**L9+S6**	End-to-End	-20.4825	-1.2838	-4.0200	13.0822	15.4695	5.1859
	Side-to-Side	-20.4823	-1.2713	-4.0258	-7.6979	12.7106	-14.7150
**S6+S11**	End-to-End	-20.4773	-1.2759	-4.0283	-10.5913	15.9422	-8.4170
	Side-to-Side	-20.4826	-1.2734	-4.0260	-7.6303	19.4715	0.0707
**L9+S11**	End-to-End	-20.4864	-1.2719	-4.0234	-16.4327	-11.3389	-6.2364
	Side-to-Side	-20.4757	-1.2724	-4.0241	-9.1905	15.0562	11.2210
**L9+S6+S11**	End-to-End	-20.4733	-1.2822	-4.0198	-16.9457	-11.0141	-5.3377
	Side-to-Side	-20.4818	-1.2710	-4.0224	-10.9127	16.9475	5.5672
	Side-to-Side2	-20.4766	-1.2740	-4.0198	1.1872	13.3396	-16.0536

Continued page of Table S8

RPs	Possible Binding Modes	GAP2					
		μ_x'' (D) [a]	μ_y'' (D) [a]	μ_z'' (D) [a]	μ_x (D) [b]	μ_y (D) [b]	μ_z (D) [b]
**L9**	End-to-End	19.9028	3.0615	0.5919	-11.9266	14.3431	-7.6077
	Side-to-Side	19.9094	3.0579	0.5785	9.0886	12.3375	-13.0863
**L10**	End-to-End	19.9090	3.0534	0.5889	-0.5898	6.9586	18.9015
	Side-to-Side	19.8990	3.0518	0.5849	-12.4165	-14.3741	-6.6965
**L11**	End-to-End	19.9002	3.0613	0.5906	19.9201	-1.7280	2.4376
	Side-to-Side	19.8916	3.0547	0.5875	14.5037	3.1013	-13.6153
**L15**	End-to-End	19.9055	3.0555	0.5788	11.6703	16.4134	-0.5521
	Side-to-Side	19.9035	3.0638	0.5844	-3.4333	-0.9986	-19.8266
**L18**	End-to-End	19.9020	3.0626	0.5835	11.9986	11.1654	11.7123
	Side-to-Side	19.9021	3.0557	0.5928	-4.4208	0.1636	-19.6523
**L20**	End-to-End	19.9093	3.0563	0.5808	1.1949	19.4622	-5.0846
	Side-to-Side	19.9121	3.0541	0.5914	-17.4192	-7.4468	-6.8766
**L22**	End-to-End	19.9104	3.0573	0.5849	4.2519	19.2066	4.3750
**S6**	End-to-End	19.9126	3.0650	0.5828	4.9479	-18.5752	-6.0602
**S8**	End-to-End	19.9007	3.0629	0.5890	16.8865	10.9787	0.2798
	Side-to-Side	19.8965	3.0537	0.5846	7.8253	-4.2939	18.0517
**S11**	End-to-End	19.9005	3.0547	0.5926	-14.0346	14.1371	2.9802
	Side-to-Side	19.9113	3.0582	0.5932	9.5352	-15.5722	-8.5295
**L10+L11**	End-to-End	19.9122	3.0519	0.5868	10.2928	1.0994	17.2917
	Side-to-Side	19.9076	3.0619	0.5884	-15.9049	-11.3221	-4.9879
**L15+L18**	End-to-End	19.8942	3.0576	0.5898	16.5852	7.9932	8.1559
	Side-to-Side	19.8967	3.0576	0.5846	17.0444	2.2173	-10.4948
**L9+S6**	End-to-End	19.9043	3.0541	0.5890	-16.1498	-9.1622	-7.8162
	Side-to-Side	19.9025	3.0583	0.5840	11.0204	-14.6773	8.3026
**S6+S11**	End-to-End	19.9046	3.0607	0.5883	5.8558	-18.9638	3.4627
	Side-to-Side	19.8985	3.0569	0.5904	8.0590	-17.0240	-7.1331
**L9+S11**	End-to-End	19.9025	3.0678	0.5846	17.7868	9.4195	-0.8747
	Side-to-Side	19.9036	3.0644	0.5814	8.6869	-9.1006	-15.7352
**L9+S6+S11**	End-to-End	19.8977	3.0563	0.5849	17.8944	9.0578	-1.8290
	Side-to-Side	19.9052	3.0591	0.5828	8.4015	-13.9001	-11.9213
	Side-to-Side2	19.8908	3.0567	0.5928	2.3749	-16.9739	10.5634

[a] Dipole moments in the center of mass of substrates calculated under Q-Chem's coordinates
[b] Dipole moments in the center of mass of substrates converted under Cartesian coordinates

Table S9. Atomic coordinates of TPI monomers within the homodimer.

	RMSD across all atom pairs of entire subunits (Å)	RMSD across all atom pairs of residues at the active sites (Å) [a]
Free TPI_subunit 1 vs 2 [b]	0.611	0.468
GAP-bound TPI_subunit 1 vs 2 [c]	0.321	0.300
DHAP-bound TPI_subunit 1 vs 2 [c]	0.326	0.793

[a] Active site residues N11, K13, H95, E165 were used to calculate RMSD.

[b] PDB entry 8TIM was used to calculate RMSD for free TPI.

[c] Energy minimized models were used to calculate RMSD.

Table S10. Electric fields at the center of substrate mass for free and ribosome-bound TPI.

RPs	Possible Binding Modes	DHAP1														
		x_c [a]	y_c [a]	z_c [a]	x_0 [b]	y_0 [b]	z_0 [b]	n_x [c]	n_y [c]	n_z [c]	E_x^R [d]	E_y^R [d]	E_z^R [d]	E_x^T [e]	E_y^T [e]	E_z^T [e]
**L9**	End-to-End	197.66	210.17	308.01	35.97	41.15	53.78	337	300	492	0.8886	-1.0256	0.4068	0.8857	-1.0318	0.4041
	Side-to-Side	195.11	214.80	307.82	35.97	41.15	49.96	331	309	499	-0.9108	-0.6183	0.6386	-0.9050	-0.5808	0.6407
**L10**	End-to-End	146.91	186.73	69.32	35.97	41.15	10.91	231	259	113	1.1201	-0.1815	-1.0702	1.1303	-0.1379	-0.9927
	Side-to-Side	146.50	148.78	83.74	35.97	41.15	9.55	230	191	144	-0.0206	0.2797	0.0561	-0.0132	0.3022	0.0526
**L11**	End-to-End	128.96	208.04	121.94	35.97	41.15	28.50	194	297	181	-2.1220	-0.2861	0.0888	-1.3763	-0.1748	0.0617
	Side-to-Side	141.07	241.83	95.12	35.97	41.15	26.12	219	357	134	-0.1750	0.6335	0.7860	-0.1872	0.6491	0.7719
**L15**	End-to-End	240.98	75.76	216.88	35.97	12.35	28.50	427	113	365	-1.2728	-0.5444	0.3147	-1.2820	-0.5155	0.3186
	Side-to-Side	227.07	70.16	202.77	35.97	19.83	28.50	398	89	337	0.1934	0.3124	0.1212	0.1769	0.3242	0.1157
**L18**	End-to-End	156.38	76.98	190.38	35.97	10.11	28.50	251	119	313	-1.1664	-0.0201	-1.1563	-1.1567	0.0010	-1.1477
	Side-to-Side	188.82	78.90	160.30	35.97	22.50	28.50	318	100	255	0.2347	0.9738	-0.2195	0.2193	0.9473	-0.2752
**L20**	End-to-End	254.23	163.40	87.57	35.97	41.15	17.28	455	217	136	-0.9831	-0.8531	0.7924	-0.9736	-0.8291	0.7906
	Side-to-Side	244.73	130.18	91.15	35.97	41.15	24.54	435	158	129	0.6074	0.4285	0.3518	0.5994	0.4388	0.3418
**L22** [f]	End-to-End	320.39	202.24	161.94	36.15	41.15	28.50	495	286	258	-0.9521	-1.5425	-0.7001	-0.9646	-1.5386	-0.7051
**S6**	End-to-End	167.96	193.93	302.81	35.97	41.15	57.09	275	272	476	-0.2790	1.0987	0.0476	-0.2558	1.0122	0.0450
**S8**	End-to-End	159.55	306.03	256.58	35.97	44.14	28.50	257	465	442	-0.9703	-1.3927	-0.2480	-0.9368	-1.3738	-0.2468
	Side-to-Side	191.56	300.60	263.30	35.97	41.15	28.50	324	461	455	0.6899	0.2972	-1.1707	0.7255	0.2642	-1.1597
**S11**	End-to-End	125.94	167.88	271.46	35.97	41.15	41.17	187	225	446	1.9653	-0.2486	-0.5017	1.6226	-0.1603	-0.4752
	Side-to-Side	166.86	170.20	288.42	35.97	41.15	36.45	273	229	488	0.4391	0.0426	0.2323	0.4258	0.0775	0.2468
**L10+L11**	End-to-End	134.47	214.02	105.01	35.97	41.15	24.14	205	307	157	-1.0784	-0.3413	-1.2286	-0.9827	-0.3538	-1.1434
	Side-to-Side	123.02	189.15	82.62	35.97	41.15	22.94	181	263	116	0.3081	0.1476	0.1826	0.2401	0.1312	0.1822
**L15+L18**	End-to-End	176.57	87.45	204.79	35.97	17.44	28.50	293	124	341	-0.9199	0.2411	-1.4036	-0.9091	0.2225	-1.3606
	Side-to-Side	200.17	53.41	202.17	35.97	11.59	28.50	342	74	336	-0.1188	0.7511	4.0405	-0.1657	0.7441	4.0159
**L9+S6**	End-to-End	189.65	195.08	307.09	35.97	41.15	59.80	320	274	479	0.5403	0.2630	0.2144	0.4652	0.2112	0.2278
	Side-to-Side	204.94	192.80	311.03	35.97	41.15	46.88	352	269	511	2.5909	4.5604	3.0827	2.5829	3.9551	3.0791
**S6+S11**	End-to-End	170.14	168.37	294.54	35.97	41.15	45.45	279	226	482	0.9290	0.9825	-0.6137	0.9500	1.0056	-0.6274
	Side-to-Side	162.81	173.68	288.33	35.97	41.15	36.71	264	236	487	1.1314	0.2294	0.9386	1.3251	0.2666	0.9835
**L9+S11**	End-to-End	185.46	174.72	289.10	35.97	41.15	38.08	311	237	486	-1.5607	0.5285	-0.1536	-1.5882	0.4737	-0.1600
	Side-to-Side	183.47	171.61	298.68	35.97	41.15	39.93	307	232	501	1.4189	0.4514	0.6846	1.1817	0.3382	0.5445
**L9+S6+S11**	End-to-End	185.94	173.04	289.18	35.97	41.15	38.24	312	234	486	-1.5514	0.5869	-0.0689	-1.4908	0.5683	-0.0753
	Side-to-Side	175.15	172.24	291.22	35.97	41.15	40.10	290	233	486	1.0631	0.2119	0.2847	1.0629	0.1779	0.2706
	Side-to-Side2	194.52	186.84	312.46	35.97	41.15	46.56	330	259	515	1.6158	0.9489	0.4215	1.5098	0.8813	0.3493

Continued page of Table S10

RPs	Possible Binding Modes	DHAP2														
		x_c [a]	y_c [a]	z_c [a]	x_0 [b]	y_0 [b]	z_0 [b]	n_x [c]	n_y [c]	n_z [c]	E_x^R [d]	E_y^R [d]	E_z^R [d]	E_x^T [e]	E_y^T [e]	E_z^T [e]
**L9**	End-to-End	230.69	217.99	321.97	35.97	41.15	53.78	406	314	519	0.9022	1.6533	1.7741	0.7703	1.5675	1.7900
	Side-to-Side	210.80	183.50	318.82	35.97	41.15	49.96	364	253	520	1.1334	0.3604	0.0094	1.1603	0.3311	0.0314
**L10**	End-to-End	116.54	175.69	51.91	35.97	41.15	10.91	168	239	79	0.3024	0.2305	0.0814	0.2993	0.2143	0.0767
	Side-to-Side	158.76	175.43	61.68	35.97	41.15	9.55	256	239	101	-0.7074	-0.4988	-0.6609	-0.7212	-0.5167	-0.6578
**L11**	End-to-End	113.53	239.66	111.51	35.97	41.15	28.50	162	353	161	0.3844	0.4752	0.0074	0.3336	0.5451	0.0177
	Side-to-Side	119.93	211.85	94.23	35.97	41.15	26.12	175	303	132	0.7836	0.3790	-0.3602	0.7818	0.3786	-0.3682
**L15**	End-to-End	250.72	44.67	199.99	35.97	12.35	28.50	447	57	332	-0.1796	0.0867	0.3760	-0.1761	0.0850	0.3799
	Side-to-Side	204.55	88.43	225.26	35.97	19.83	28.50	351	122	381	-0.6968	-0.0883	-0.9130	-0.6603	-0.0890	-0.8587
**L18**	End-to-End	160.99	40.71	187.17	35.97	10.11	28.50	260	54	307	-0.1939	0.0985	-0.3163	-0.1726	0.1048	-0.2932
	Side-to-Side	162.92	74.09	185.86	35.97	22.50	28.50	264	92	305	-0.2908	-0.1411	-1.1744	-0.2917	-0.1169	-1.1034
**L20**	End-to-End	275.60	138.06	71.81	35.97	41.15	17.28	499	172	106	0.0157	0.1020	0.2114	0.0232	0.1134	0.2138
	Side-to-Side	253.32	165.67	87.41	35.97	41.15	24.54	453	221	122	-0.9527	0.6418	-0.4523	-0.9600	0.6665	-0.4454
**L22** [f]	End-to-End	346.66	176.69	160.00	36.15	41.15	28.50	541	241	255	0.3561	-0.2448	-0.1273	0.2700	-0.2710	-0.1013
**S6**	End-to-End	153.28	200.09	335.88	35.97	41.15	57.09	244	282	540	0.0352	-1.1989	0.0775	0.0392	-1.1833	0.0723
**S8**	End-to-End	128.49	318.12	271.96	35.97	44.14	28.50	193	487	471	-0.0324	0.2504	1.4992	-0.0645	0.2765	1.4617
	Side-to-Side	155.52	298.03	256.84	35.97	41.15	28.50	249	456	442	0.4063	0.3039	0.4939	0.3906	0.2973	0.4565
**S11**	End-to-End	139.42	147.99	299.21	35.97	41.15	41.17	215	190	500	-0.6467	0.6227	0.4417	-0.6920	0.6438	0.4448
	Side-to-Side	131.83	177.93	280.64	35.97	41.15	36.45	200	243	473	0.3558	-0.9751	-0.3853	0.3178	-0.9610	-0.3721
**L10+L11**	End-to-End	131.22	242.96	82.68	35.97	41.15	24.14	198	359	113	-0.2622	-0.1923	-0.1853	-0.2353	-0.1650	-0.1508
	Side-to-Side	120.69	215.41	108.15	35.97	41.15	22.94	176	310	165	-1.1810	-0.2073	0.3350	-1.1861	-0.1924	0.3548
**L15+L18**	End-to-End	161.31	56.92	218.27	35.97	17.44	28.50	261	70	367	-0.2048	0.2655	-0.1873	-0.2196	0.2277	-0.2080
	Side-to-Side	169.80	72.20	193.72	35.97	11.59	28.50	279	108	320	0.7433	0.0958	-0.6040	0.7052	0.1226	-0.6139
**L9+S6**	End-to-End	187.58	214.54	338.14	35.97	41.15	59.80	316	308	539	-1.0062	-0.2887	0.4819	-1.0238	-0.3044	0.4713
	Side-to-Side	175.38	185.44	290.57	35.97	41.15	46.88	290	256	472	0.7893	-0.6072	0.7860	0.7439	-0.5312	0.7094
**S6+S11**	End-to-End	138.35	181.34	307.49	35.97	41.15	45.45	213	249	507	0.1121	-0.9817	0.4172	0.0760	-0.9473	0.4244
	Side-to-Side	127.61	182.24	282.45	35.97	41.15	36.71	191	251	476	0.3411	-0.9544	-0.2628	0.3443	-0.9489	-0.2676
**L9+S11**	End-to-End	183.17	138.19	291.85	35.97	41.15	38.08	307	172	491	0.2472	0.5436	0.2366	0.2450	0.5104	0.2338
	Side-to-Side	147.25	177.42	299.91	35.97	41.15	39.93	232	242	503	0.3823	-0.7896	-0.9596	0.3847	-0.7902	-0.9427
**L9+S6+S11**	End-to-End	183.07	136.57	292.14	35.97	41.15	38.24	306	170	492	-0.0001	0.1883	0.1173	-0.0247	0.1602	0.1212
	Side-to-Side	139.09	172.43	298.09	35.97	41.15	40.10	215	233	499	0.4833	-1.0605	-0.7417	0.4476	-1.0439	-0.7273
	Side-to-Side2	169.47	189.53	285.78	35.97	41.15	46.56	278	264	463	0.1857	-0.8679	0.8944	0.2031	-0.5944	0.6337

Continued page of Table S10

RPs	Possible Binding Modes	GAP1														
		x_c [a]	y_c [a]	z_c [a]	x_0 [b]	y_0 [b]	z_0 [b]	n_x [c]	n_y [c]	n_z [c]	E_x^{R} [d]	E_y^{R} [d]	E_z^{R} [d]	E_x^{T} [e]	E_y^{T} [e]	E_z^{T} [e]
**L9**	End-to-End	197.69	209.88	308.11	35.97	41.15	53.78	337	300	492	0.7373	-0.6762	0.1921	0.7239	-0.6357	0.1782
	Side-to-Side	194.89	214.74	308.00	35.97	41.15	49.96	331	308	500	-1.3151	-0.4236	0.1871	-1.2866	-0.4398	0.1967
**L10**	End-to-End	147.07	186.67	69.07	35.97	41.15	10.91	231	259	113	1.2047	-0.1704	-0.5486	1.2100	-0.1103	-0.4751
	Side-to-Side	146.65	148.91	83.95	35.97	41.15	9.55	231	191	144	0.0337	0.1975	0.1338	-0.0081	0.1962	0.1126
**L11**	End-to-End	128.70	207.92	121.93	35.97	41.15	28.50	193	296	181	-1.7412	0.1414	0.0807	-1.1583	0.1215	0.0688
	Side-to-Side	140.93	241.91	95.37	35.97	41.15	26.12	219	357	134	0.1531	1.0655	0.8638	0.1285	0.9702	0.9928
**L15**	End-to-End	240.75	75.63	216.97	35.97	12.35	28.50	427	112	365	-1.3638	-0.0580	0.3390	-1.3642	-0.0534	0.3714
	Side-to-Side	227.19	70.12	202.97	35.97	19.83	28.50	398	89	338	0.6608	0.4325	0.0026	0.6318	0.3631	-0.0863
**L18**	End-to-End	156.13	76.97	190.23	35.97	10.11	28.50	250	119	313	-0.7118	0.0494	-1.0429	-0.6912	0.0283	-1.0477
	Side-to-Side	189.05	78.89	160.53	35.97	22.50	28.50	319	100	256	0.6071	1.3670	0.0627	0.6141	1.3578	-0.0022
**L20**	End-to-End	254.12	163.20	87.73	35.97	41.15	17.28	454	217	136	-0.9754	-0.9516	0.1911	-0.9826	-0.9770	0.1560
	Side-to-Side	244.99	130.15	91.29	35.97	41.15	24.54	435	158	129	0.1321	0.2625	0.1954	0.1145	0.2647	0.1911
**L22** [f]	End-to-End	320.20	202.04	161.88	36.15	41.15	28.50	495	286	258	-0.6170	-0.9632	-0.8138	-0.6041	-0.9317	-0.8147
**S6**	End-to-End	167.95	194.22	302.76	35.97	41.15	57.09	275	272	476	-0.1814	0.6036	-0.0460	-0.0774	0.5178	-0.0524
**S8**	End-to-End	159.41	305.78	256.51	35.97	44.14	28.50	257	465	441	-0.7278	-0.9072	0.0858	-0.7116	-0.8788	0.0896
	Side-to-Side	191.59	300.69	263.02	35.97	41.15	28.50	324	461	454	0.6962	-0.2572	-1.4503	0.7499	-0.2327	-1.4401
**S11**	End-to-End	126.10	167.72	271.28	35.97	41.15	41.17	188	225	445	1.0510	-0.5062	-0.0467	0.9380	-0.4195	-0.0438
	Side-to-Side	166.85	170.41	288.61	35.97	41.15	36.45	273	230	488	0.8215	0.0495	0.7389	1.0223	0.0721	0.7230
**L10+L11**	End-to-End	134.32	213.86	104.82	35.97	41.15	24.14	205	307	156	-1.0437	-0.2930	-0.8170	-0.9625	-0.3346	-0.6959
	Side-to-Side	123.32	189.20	82.59	35.97	41.15	22.94	182	263	115	0.2903	0.3194	0.3234	0.2505	0.2706	0.3397
**L15+L18**	End-to-End	176.36	87.47	204.59	35.97	17.44	28.50	292	124	341	-0.4801	0.0587	-1.3804	-0.4749	0.0829	-1.3806
	Side-to-Side	200.02	53.28	202.39	35.97	11.59	28.50	342	74	337	-0.3963	0.0965	0.9807	-0.4314	0.1193	0.9944
**L9+S6**	End-to-End	189.93	195.13	307.08	35.97	41.15	59.80	321	274	479	0.3068	0.2815	0.0457	0.2246	0.2268	0.0579
	Side-to-Side	204.88	193.07	310.99	35.97	41.15	46.88	352	270	511	0.6656	4.0190	0.3722	0.7293	3.9598	0.4725
**S6+S11**	End-to-End	170.19	168.64	294.44	35.97	41.15	45.45	280	227	482	0.0111	0.6636	-0.6082	0.0005	0.6719	-0.6010
	Side-to-Side	162.83	173.92	288.51	35.97	41.15	36.71	264	236	487	4.2342	-0.1131	3.1836	4.2619	-0.0472	3.0870
**L9+S11**	End-to-End	185.18	174.73	289.14	35.97	41.15	38.08	311	237	486	-1.3738	0.5613	-0.5664	-1.4268	0.5114	-0.5533
	Side-to-Side	183.49	171.70	298.95	35.97	41.15	39.93	307	232	501	2.6080	-0.1758	0.1745	2.5986	-0.2685	0.0683
**L9+S6+S11**	End-to-End	185.67	173.06	289.19	35.97	41.15	38.24	312	234	486	-1.4363	0.6038	-0.4199	-1.3833	0.5588	-0.4319
	Side-to-Side	175.17	172.47	291.39	35.97	41.15	40.10	290	233	486	3.6886	-0.1499	0.6312	3.6500	-0.1527	0.6088
	Side-to-Side2	194.58	187.11	312.42	35.97	41.15	46.56	330	259	515	3.1241	1.3528	2.5279	3.0689	1.1094	2.5965

Continued page of Table S10

RPs	Possible Binding Modes	GAP2														
		x_c [a]	y_c [a]	z_c [a]	x_0 [b]	y_0 [b]	z_0 [b]	n_x [c]	n_y [c]	n_z [c]	E_x^{R} [d]	E_y^{R} [d]	E_z^{R} [d]	E_x^{T} [e]	E_y^{T} [e]	E_z^{T} [e]
**L9**	End-to-End	230.66	218.04	322.03	35.97	41.15	53.78	406	314	519	0.9221	0.6164	1.2514	0.9342	0.4045	1.2550
	Side-to-Side	210.87	183.52	318.85	35.97	41.15	49.96	364	253	521	0.1352	0.0416	0.1405	0.1293	0.0379	0.1505
**L10**	End-to-End	116.51	175.76	51.91	35.97	41.15	10.91	168	239	79	0.3594	0.2198	-0.2189	0.3319	0.2598	-0.2093
	Side-to-Side	158.69	175.47	61.66	35.97	41.15	9.55	256	239	101	-0.5742	-0.4304	-0.7512	-0.5740	-0.4507	-0.7441
**L11**	End-to-End	113.57	239.68	111.42	35.97	41.15	28.50	162	353	161	0.0755	0.4004	-0.1212	0.1007	0.3743	-0.1109
	Side-to-Side	119.99	211.78	94.23	35.97	41.15	26.12	175	303	132	0.7112	0.3135	-0.2327	0.7275	0.3168	-0.2481
**L15**	End-to-End	250.79	44.65	200.03	35.97	12.35	28.50	447	57	332	-0.2456	-0.1449	0.4780	-0.2164	-0.1254	0.4756
	Side-to-Side	204.44	88.38	225.25	35.97	19.83	28.50	351	122	381	-0.7753	0.2087	-0.6949	-0.7342	0.1804	-0.6585
**L18**	End-to-End	160.98	40.70	187.24	35.97	10.11	28.50	260	54	307	-0.4990	-0.1281	-0.4038	-0.5176	-0.1469	-0.3895
	Side-to-Side	162.89	73.99	185.81	35.97	22.50	28.50	264	91	305	-0.4837	-0.2843	-1.0953	-0.4385	-0.2349	-1.0574
**L20**	End-to-End	275.68	138.09	71.83	35.97	41.15	17.28	499	172	106	0.1052	-0.1416	0.2273	0.1188	-0.1008	0.2226
	Side-to-Side	253.25	165.71	87.45	35.97	41.15	24.54	453	221	122	-0.9058	0.8210	-0.6176	-0.8971	0.8210	-0.6159
**L22** [f]	End-to-End	346.69	176.69	160.08	36.15	41.15	28.50	541	241	255	0.0249	-0.6669	-0.0408	0.0142	-0.6919	-0.0747
**S6**	End-to-End	153.34	200.07	335.93	35.97	41.15	57.09	244	282	540	0.0087	-1.2094	0.0683	0.0019	-1.2031	0.0731
**S8**	End-to-End	128.48	318.18	271.92	35.97	44.14	28.50	193	487	471	-0.3832	-0.0903	0.6165	-0.3462	-0.0532	0.7398
	Side-to-Side	155.49	298.09	256.90	35.97	41.15	28.50	249	457	442	0.1339	0.1792	0.0248	0.1464	0.1956	0.0136
**S11**	End-to-End	139.35	147.95	299.24	35.97	41.15	41.17	215	190	500	-0.4523	0.4890	0.4600	-0.5199	0.5443	0.4808
	Side-to-Side	131.79	177.84	280.67	35.97	41.15	36.45	200	243	473	0.2809	-0.9255	-0.4823	0.2488	-0.8552	-0.4679
**L10+L11**	End-to-End	131.30	242.97	82.66	35.97	41.15	24.14	199	359	113	-0.2122	0.0425	-0.2560	-0.2002	0.0320	-0.2363
	Side-to-Side	120.67	215.36	108.21	35.97	41.15	22.94	176	310	165	-1.2675	-0.0550	0.3396	-1.2613	-0.0398	0.3285
**L15+L18**	End-to-End	161.28	56.95	218.34	35.97	17.44	28.50	261	70	368	-0.1487	0.3282	-0.1090	-0.1463	0.3323	-0.1014
	Side-to-Side	169.79	72.18	193.65	35.97	11.59	28.50	279	108	320	0.3614	0.1681	-0.3517	0.3393	0.1694	-0.3617
**L9+S6**	End-to-End	187.56	214.48	338.19	35.97	41.15	59.80	316	308	539	-1.0703	-0.0272	0.5587	-1.0606	-0.0252	0.5507
	Side-to-Side	175.31	185.37	290.63	35.97	41.15	46.88	290	256	472	0.6101	-0.4538	0.6889	0.5665	-0.4122	0.6609
**S6+S11**	End-to-End	138.35	181.34	307.58	35.97	41.15	45.45	213	249	507	0.1209	-0.9700	0.3991	0.1158	-0.9627	0.4015
	Side-to-Side	127.57	182.18	282.50	35.97	41.15	36.71	191	251	476	0.2632	-0.8919	-0.2983	0.2332	-0.8437	-0.2845
**L9+S11**	End-to-End	183.21	138.18	291.91	35.97	41.15	38.08	307	172	491	0.0747	0.4951	0.3044	0.0517	0.4912	0.3051
	Side-to-Side	147.23	177.35	299.92	35.97	41.15	39.93	232	242	503	0.2131	-0.6714	-0.9504	0.2271	-0.6572	-0.9566
**L9+S6+S11**	End-to-End	183.12	136.56	292.19	35.97	41.15	38.24	306	170	492	-0.1852	-0.0313	0.1720	-0.1948	-0.0282	0.1775
	Side-to-Side	139.08	172.35	298.13	35.97	41.15	40.10	215	233	500	0.2838	-0.8881	-0.5723	0.2539	-0.8374	-0.5481
	Side-to-Side2	169.40	189.52	285.82	35.97	41.15	46.56	278	264	463	0.0287	-0.8515	0.8685	0.1165	-0.4866	0.6405

[a] Coordinates at the center of mass for substrates in Cartesian coordinates.
[b] The new origin with APBS's grid setting corresponding to the minimum coordinates of input PDB file.

Continued page of Table S10

[c] The center of mass of substrates inside the potential grid box under different binding conformations.
[d] Electric fields of ribosome bound TPI at the center of mass positions of substrates in $k_BT(e\text{Å})^{-1}$ units.
[e] Electric fields of free TPI at the center of mass positions of substrates in $k_BT(e\text{Å})^{-1}$ units.
[f] A longer grid length of ~560 Å along x was set for the TPI-ribosome complexes at the binding interface with L22.

Table S11. Magnitude and orientation of substrate dipole and electric field vectors.

RPs	Possible Binding Modes	DHAP1					GAP1				
		|μ| (D)	$\|E^R\|$ (V/Å)	θ^R (°) [a]	$\|E^T\|$ (V/Å)	θ^T (°) [b]	|μ| (D)	$\|E^R\|$ (V/Å)	θ^R (°) [a]	$\|E^T\|$ (V/Å)	θ^T (°) [b]
**L9**	End-to-End	19.51	0.036	6.3	0.036	6.3	20.91	0.026	7.7	0.025	7.1
	Side-to-Side	19.51	0.033	18.1	0.032	19.4	20.92	0.036	40.5	0.035	39.4
**L10**	End-to-End	19.51	0.040	48.6	0.039	51.4	20.92	0.034	67.6	0.034	71.5
	Side-to-Side	19.51	0.007	54.5	0.008	53.1	20.91	0.006	52.0	0.006	61.2
**L11**	End-to-End	19.52	0.055	17.5	0.036	17.0	20.91	0.045	13.7	0.030	12.2
	Side-to-Side	19.52	0.026	60.0	0.026	60.0	20.91	0.035	84.4	0.036	81.2
**L15**	End-to-End	19.52	0.036	34.3	0.036	35.3	20.91	0.036	54.8	0.036	55.7
	Side-to-Side	19.52	0.010	52.8	0.010	54.1	20.91	0.020	63.9	0.019	71.4
**L18**	End-to-End	19.52	0.042	34.4	0.042	35.1	20.91	0.032	37.3	0.032	36.1
	Side-to-Side	19.52	0.026	90.0	0.026	93.4	20.92	0.038	68.9	0.038	71.4
**L20**	End-to-End	19.52	0.039	45.2	0.039	45.8	20.91	0.035	34.5	0.036	33.5
	Side-to-Side	19.51	0.021	21.6	0.021	21.8	20.91	0.009	52.6	0.009	54.9
**L22**	End-to-End	19.51	0.050	20.6	0.050	21.0	20.91	0.036	23.4	0.035	23.7
**S6**	End-to-End	19.51	0.029	17.0	0.027	17.0	20.90	0.016	25.9	0.014	33.3
**S8**	End-to-End	19.51	0.044	25.6	0.043	26.1	20.91	0.030	19.4	0.029	19.0
	Side-to-Side	19.53	0.036	54.1	0.036	55.4	20.91	0.042	49.2	0.042	51.1
**S11**	End-to-End	19.52	0.053	28.0	0.044	30.1	20.92	0.030	2.2	0.026	3.0
	Side-to-Side	19.51	0.013	100.9	0.013	96.3	20.91	0.028	101.9	0.032	103.5
**L10+L11**	End-to-End	19.51	0.043	13.6	0.040	15.1	20.90	0.035	20.2	0.032	23.8
	Side-to-Side	19.52	0.010	27.7	0.008	30.4	20.92	0.014	34.4	0.013	40.2
**L15+L18**	End-to-End	19.52	0.044	44.6	0.042	44.0	20.91	0.038	48.6	0.038	49.6
	Side-to-Side	19.52	0.106	50.5	0.105	49.8	20.90	0.027	27.2	0.028	25.5
**L9+S6**	End-to-End	19.51	0.016	14.0	0.014	16.4	20.91	0.011	10.8	0.008	6.0
	Side-to-Side	19.51	0.156	88.1	0.145	92.1	20.91	0.105	61.8	0.104	63.5
**S6+S11**	End-to-End	19.50	0.038	63.5	0.039	63.5	20.91	0.023	34.1	0.023	33.2
	Side-to-Side	19.50	0.038	92.0	0.043	92.9	20.91	0.136	108.0	0.135	107.6
**L9+S11**	End-to-End	19.51	0.043	48.9	0.043	46.8	20.92	0.041	53.5	0.041	51.3
	Side-to-Side	19.51	0.042	85.7	0.035	87.3	20.91	0.067	116.8	0.067	119.8
**L9+S6+S11**	End-to-End	19.52	0.043	49.9	0.041	50.0	20.90	0.041	53.8	0.040	52.9
	Side-to-Side	19.52	0.029	100.9	0.029	102.7	20.91	0.096	120.1	0.095	120.3
	Side-to-Side2	19.52	0.049	77.3	0.046	76.4	20.91	0.109	102.3	0.107	105.5

Continued page of Table S11

RPs	Possible Binding Modes	DHAP2					GAP2				
		|μ| (D)	$\|E^R\|$ (V/Å)	$θ^R$ (º) [a]	$\|E^T\|$ (V/Å)	$θ^T$ (º) [b]	|μ| (D)	$\|E^R\|$ (V/Å)	$θ^R$ (º) [a]	$\|E^T\|$ (V/Å)	$θ^T$ (º) [b]
**L9**	End-to-End	21.74	0.066	86.7	0.064	86.3	20.15	0.043	110.3	0.042	117.2
	Side-to-Side	21.73	0.031	47.9	0.031	49.8	20.15	0.005	91.4	0.005	94.6
**L10**	End-to-End	21.73	0.010	66.1	0.010	67.1	20.15	0.012	107.1	0.012	104.3
	Side-to-Side	21.72	0.028	20.2	0.028	19.3	20.14	0.027	28.7	0.027	27.7
**L11**	End-to-End	21.73	0.016	56.9	0.016	64.4	20.14	0.011	86.5	0.010	82.3
	Side-to-Side	21.73	0.024	22.5	0.024	22.2	20.13	0.021	27.8	0.021	27.0
**L15**	End-to-End	21.73	0.011	93.6	0.011	93.5	20.15	0.014	119.4	0.014	116.6
	Side-to-Side	21.73	0.030	23.9	0.028	24.1	20.15	0.027	40.6	0.026	40.3
**L18**	End-to-End	21.73	0.010	132.7	0.009	130.3	20.14	0.017	157.1	0.017	158.1
	Side-to-Side	21.72	0.031	1.9	0.029	2.3	20.14	0.032	17.7	0.030	15.5
**L20**	End-to-End	21.72	0.006	74.7	0.006	72.4	20.15	0.007	130.8	0.007	122.6
	Side-to-Side	21.73	0.032	51.9	0.032	52.6	20.15	0.035	59.7	0.035	60.0
**L22**	End-to-End	21.72	0.012	115.3	0.010	124.9	20.15	0.017	162.9	0.018	165.1
**S6**	End-to-End	21.74	0.031	25.9	0.030	25.6	20.16	0.031	25.2	0.031	25.6
**S8**	End-to-End	21.72	0.039	89.3	0.038	89.5	20.14	0.019	119.7	0.021	112.2
	Side-to-Side	21.72	0.018	36.6	0.017	37.9	20.14	0.006	80.8	0.006	83.6
**S11**	End-to-End	21.73	0.026	18.5	0.027	17.3	20.14	0.021	26.2	0.023	24.1
	Side-to-Side	21.72	0.028	6.9	0.028	8.5	20.15	0.028	13.2	0.026	13.9
**L10+L11**	End-to-End	21.72	0.010	146.5	0.008	145.4	20.15	0.009	166.3	0.008	166.9
	Side-to-Side	21.73	0.032	38.3	0.032	39.4	20.15	0.034	43.8	0.034	44.1
**L15+L18**	End-to-End	21.73	0.010	109.3	0.010	115.7	20.14	0.010	95.5	0.010	94.5
	Side-to-Side	21.72	0.025	4.5	0.024	7.1	20.14	0.014	17.2	0.013	19.4
**L9+S6**	End-to-End	21.72	0.030	47.1	0.030	46.1	20.15	0.031	57.2	0.031	57.2
	Side-to-Side	21.73	0.033	18.2	0.030	19.6	20.14	0.026	22.4	0.025	23.6
**S6+S11**	End-to-End	21.73	0.028	14.8	0.027	16.9	20.15	0.027	15.6	0.027	16.0
	Side-to-Side	21.72	0.027	2.6	0.027	2.2	20.14	0.025	8.9	0.024	9.7
**L9+S11**	End-to-End	21.72	0.017	41.1	0.016	40.3	20.15	0.015	61.0	0.015	63.2
	Side-to-Side	21.72	0.033	7.6	0.033	7.5	20.15	0.030	15.9	0.030	15.1
**L9+S6+S11**	End-to-End	21.72	0.006	67.5	0.005	75.6	20.14	0.007	139.7	0.007	139.6
	Side-to-Side	21.73	0.035	4.4	0.035	5.5	20.15	0.028	12.1	0.027	12.5
	Side-to-Side2	21.72	0.032	11.8	0.023	15.4	20.13	0.031	14.7	0.021	20.8

[a] Angles between molecular dipole and electric field vectors exerted by ribosome-bound TPI.
[b] Angles between molecular dipole and electric field vectors exerted by free TPI.

Table S12. Substrate dipole-electric field interaction energies for free and ribosome-bound TPI.

RPs	Possible Binding Modes	DHAP1									
		$E_x{}^R$ (V/Å)	$E_y{}^R$ (V/Å)	$E_z{}^R$ (V/Å)	$E_x{}^T$ (V/Å)	$E_y{}^T$ (V/Å)	$E_z{}^T$ (V/Å)	U^{Ribo}(eV) [a]	U^{TPI} (eV) [b]	U^{Ribo} (J) [a]	U^{TPI} (J) [b]
**L9**	End-to-End	0.0228	-0.0264	0.0105	0.0228	-0.0265	0.0104	-0.1470	-0.1472	-2.355E-20	-2.358E-20
	Side-to-Side	-0.0234	-0.0159	0.0164	-0.0233	-0.0149	0.0165	-0.1263	-0.1233	-2.023E-20	-1.975E-20
**L10**	End-to-End	0.0288	-0.0047	-0.0275	0.0290	-0.0035	-0.0255	-0.1077	-0.0985	-1.726E-20	-1.577E-20
	Side-to-Side	-0.0005	0.0072	0.0014	-0.0003	0.0078	0.0014	-0.0173	-0.0192	-2.776E-21	-3.082E-21
**L11**	End-to-End	-0.0545	-0.0074	0.0023	-0.0354	-0.0045	0.0016	-0.2135	-0.1387	-3.420E-20	-2.222E-20
	Side-to-Side	-0.0045	0.0163	0.0202	-0.0048	0.0167	0.0198	-0.0534	-0.0536	-8.562E-21	-8.587E-21
**L15**	End-to-End	-0.0327	-0.0140	0.0081	-0.0329	-0.0132	0.0082	-0.1225	-0.1208	-1.962E-20	-1.935E-20
	Side-to-Side	0.0050	0.0080	0.0031	0.0045	0.0083	0.0030	-0.0244	-0.0237	-3.908E-21	-3.799E-21
**L18**	End-to-End	-0.0300	-0.0005	-0.0297	-0.0297	0.0000	-0.0295	-0.1415	-0.1392	-2.266E-20	-2.230E-20
	Side-to-Side	0.0060	0.0250	-0.0056	0.0056	0.0243	-0.0071	-0.0001	0.0063	-1.454E-23	1.011E-21
**L20**	End-to-End	-0.0253	-0.0219	0.0204	-0.0250	-0.0213	0.0203	-0.1121	-0.1094	-1.795E-20	-1.753E-20
	Side-to-Side	0.0156	0.0110	0.0090	0.0154	0.0113	0.0088	-0.0798	-0.0792	-1.278E-20	-1.269E-20
**L22**	End-to-End	-0.0245	-0.0396	-0.0180	-0.0248	-0.0395	-0.0181	-0.1898	-0.1898	-3.040E-20	-3.041E-20
**S6**	End-to-End	-0.0072	0.0282	0.0012	-0.0066	0.0260	0.0012	-0.1132	-0.1043	-1.814E-20	-1.671E-20
**S8**	End-to-End	-0.0249	-0.0358	-0.0064	-0.0241	-0.0353	-0.0063	-0.1614	-0.1575	-2.586E-20	-2.524E-20
	Side-to-Side	0.0177	0.0076	-0.0301	0.0186	0.0068	-0.0298	-0.0852	-0.0827	-1.365E-20	-1.325E-20
**S11**	End-to-End	0.0505	-0.0064	-0.0129	0.0417	-0.0041	-0.0122	-0.1883	-0.1534	-3.017E-20	-2.458E-20
	Side-to-Side	0.0113	0.0011	0.0060	0.0109	0.0020	0.0063	0.0098	0.0057	1.576E-21	9.133E-22
**L10+L11**	End-to-End	-0.0277	-0.0088	-0.0316	-0.0252	-0.0091	-0.0294	-0.1694	-0.1561	-2.713E-20	-2.500E-20
	Side-to-Side	0.0079	0.0038	0.0047	0.0062	0.0034	0.0047	-0.0358	-0.0296	-5.734E-21	-4.740E-21
**L15+L18**	End-to-End	-0.0236	0.0062	-0.0361	-0.0234	0.0057	-0.0350	-0.1261	-0.1241	-2.020E-20	-1.988E-20
	Side-to-Side	-0.0031	0.0193	0.1038	-0.0043	0.0191	0.1032	-0.2732	-0.2755	-4.378E-20	-4.413E-20
**L9+S6**	End-to-End	0.0139	0.0068	0.0055	0.0120	0.0054	0.0059	-0.0646	-0.0560	-1.035E-20	-8.973E-21
	Side-to-Side	0.0666	0.1172	0.0792	0.0664	0.1016	0.0791	-0.0208	0.0219	-3.337E-21	3.508E-21
**S6+S11**	End-to-End	0.0239	0.0252	-0.0158	0.0244	0.0258	-0.0161	-0.0691	-0.0707	-1.107E-20	-1.133E-20
	Side-to-Side	0.0291	0.0059	0.0241	0.0340	0.0068	0.0253	0.0055	0.0087	8.856E-22	1.389E-21
**L9+S11**	End-to-End	-0.0401	0.0136	-0.0039	-0.0408	0.0122	-0.0041	-0.1134	-0.1188	-1.817E-20	-1.904E-20
	Side-to-Side	0.0365	0.0116	0.0176	0.0304	0.0087	0.0140	-0.0130	-0.0066	-2.076E-21	-1.057E-21
**L9+S6+S11**	End-to-End	-0.0399	0.0151	-0.0018	-0.0383	0.0146	-0.0019	-0.1117	-0.1072	-1.789E-20	-1.717E-20
	Side-to-Side	0.0273	0.0054	0.0073	0.0273	0.0046	0.0070	0.0222	0.0256	3.554E-21	4.094E-21
	Side-to-Side2	0.0415	0.0244	0.0108	0.0388	0.0226	0.0090	-0.0441	-0.0438	-7.058E-21	-7.022E-21

Continued page of Table S12

RPs	Possible Binding Modes	DHAP2									
		$E_x{}^R$ (V/Å)	$E_y{}^R$ (V/Å)	$E_z{}^R$ (V/Å)	$E_x{}^T$ (V/Å)	$E_y{}^T$ (V/Å)	$E_z{}^T$ (V/Å)	U^{Ribo}(*eV*) [a]	U^{TPI} (*eV*) [b]	U^{Ribo} (J) [a]	U^{TPI} (J) [b]
**L9**	End-to-End	0.0232	0.0425	0.0456	0.0198	0.0403	0.0460	-0.0171	-0.0188	-2.734E-21	-3.016E-21
	Side-to-Side	0.0291	0.0093	0.0002	0.0298	0.0085	0.0008	-0.0927	-0.0906	-1.485E-20	-1.451E-20
**L10**	End-to-End	0.0078	0.0059	0.0021	0.0077	0.0055	0.0020	-0.0183	-0.0170	-2.929E-21	-2.722E-21
	Side-to-Side	-0.0182	-0.0128	-0.0170	-0.0185	-0.0133	-0.0169	-0.1188	-0.1211	-1.903E-20	-1.940E-20
**L11**	End-to-End	0.0099	0.0122	0.0002	0.0086	0.0140	0.0005	-0.0388	-0.0321	-6.213E-21	-5.142E-21
	Side-to-Side	0.0201	0.0097	-0.0093	0.0201	0.0097	-0.0095	-0.1011	-0.1015	-1.620E-20	-1.627E-20
**L15**	End-to-End	-0.0046	0.0022	0.0097	-0.0045	0.0022	0.0098	0.0031	0.0030	5.044E-22	4.841E-22
	Side-to-Side	-0.0179	-0.0023	-0.0235	-0.0170	-0.0023	-0.0221	-0.1224	-0.1153	-1.961E-20	-1.848E-20
**L18**	End-to-End	-0.0050	0.0025	-0.0081	-0.0044	0.0027	-0.0075	0.0303	0.0268	4.849E-21	4.291E-21
	Side-to-Side	-0.0075	-0.0036	-0.0302	-0.0075	-0.0030	-0.0283	-0.1414	-0.1332	-2.266E-20	-2.134E-20
**L20**	End-to-End	0.0004	0.0026	0.0054	0.0006	0.0029	0.0055	-0.0072	-0.0086	-1.154E-21	-1.371E-21
	Side-to-Side	-0.0245	0.0165	-0.0116	-0.0247	0.0171	-0.0114	-0.0886	-0.0882	-1.419E-20	-1.413E-20
**L22**	End-to-End	0.0091	-0.0063	-0.0033	0.0069	-0.0070	-0.0026	0.0224	0.0263	3.585E-21	4.218E-21
**S6**	End-to-End	0.0009	-0.0308	0.0020	0.0010	-0.0304	0.0019	-0.1257	-0.1244	-2.015E-20	-1.993E-20
**S8**	End-to-End	-0.0008	0.0064	0.0385	-0.0017	0.0071	0.0376	-0.0023	-0.0014	-3.634E-22	-2.203E-22
	Side-to-Side	0.0104	0.0078	0.0127	0.0100	0.0076	0.0117	-0.0661	-0.0615	-1.058E-20	-9.850E-21
**S11**	End-to-End	-0.0166	0.0160	0.0113	-0.0178	0.0165	0.0114	-0.1103	-0.1159	-1.767E-20	-1.857E-20
	Side-to-Side	0.0091	-0.0251	-0.0099	0.0082	-0.0247	-0.0096	-0.1277	-0.1239	-2.046E-20	-1.985E-20
**L10+L11**	End-to-End	-0.0067	-0.0049	-0.0048	-0.0060	-0.0042	-0.0039	0.0362	0.0310	5.806E-21	4.974E-21
	Side-to-Side	-0.0303	-0.0053	0.0086	-0.0305	-0.0049	0.0091	-0.1136	-0.1126	-1.820E-20	-1.803E-20
**L15+L18**	End-to-End	-0.0053	0.0068	-0.0048	-0.0056	0.0059	-0.0053	0.0147	0.0191	2.358E-21	3.063E-21
	Side-to-Side	0.0191	0.0025	-0.0155	0.0181	0.0031	-0.0158	-0.1115	-0.1087	-1.786E-20	-1.741E-20
**L9+S6**	End-to-End	-0.0259	-0.0074	0.0124	-0.0263	-0.0078	0.0121	-0.0912	-0.0941	-1.461E-20	-1.508E-20
	Side-to-Side	0.0203	-0.0156	0.0202	0.0191	-0.0136	0.0182	-0.1401	-0.1267	-2.244E-20	-2.031E-20
**S6+S11**	End-to-End	0.0029	-0.0252	0.0107	0.0020	-0.0243	0.0109	-0.1206	-0.1158	-1.932E-20	-1.855E-20
	Side-to-Side	0.0088	-0.0245	-0.0068	0.0088	-0.0244	-0.0069	-0.1215	-0.1212	-1.947E-20	-1.942E-20
**L9+S11**	End-to-End	0.0064	0.0140	0.0061	0.0063	0.0131	0.0060	-0.0563	-0.0542	-9.014E-21	-8.689E-21
	Side-to-Side	0.0098	-0.0203	-0.0247	0.0099	-0.0203	-0.0242	-0.1497	-0.1484	-2.399E-20	-2.378E-20
**L9+S6+S11**	End-to-End	0.0000	0.0048	0.0030	-0.0006	0.0041	0.0031	-0.0099	-0.0059	-1.581E-21	-9.391E-22
	Side-to-Side	0.0124	-0.0272	-0.0191	0.0115	-0.0268	-0.0187	-0.1601	-0.1560	-2.565E-20	-2.500E-20
	Side-to-Side2	0.0048	-0.0223	0.0230	0.0052	-0.0153	0.0163	-0.1433	-0.1000	-2.296E-20	-1.601E-20

Continued page of Table S12

RPs	Possible Binding Modes	GAP1									
		$E_x{}^R$ (V/Å)	$E_y{}^R$ (V/Å)	$E_z{}^R$ (V/Å)	$E_x{}^T$ (V/Å)	$E_y{}^T$ (V/Å)	$E_z{}^T$ (V/Å)	U^{Ribo}(*eV*) [a]	U^{TPI} (*eV*) [b]	U^{Ribo} (J) [a]	U^{TPI} (J) [b]
**L9**	End-to-End	0.0189	-0.0174	0.0049	0.0186	-0.0163	0.0046	-0.1129	-0.1087	-1.809E-20	-1.742E-20
	Side-to-Side	-0.0338	-0.0109	0.0048	-0.0331	-0.0113	0.0051	-0.1186	-0.1188	-1.899E-20	-1.903E-20
**L10**	End-to-End	0.0310	-0.0044	-0.0141	0.0311	-0.0028	-0.0122	-0.0569	-0.0463	-9.121E-21	-7.419E-21
	Side-to-Side	0.0009	0.0051	0.0034	-0.0002	0.0050	0.0029	-0.0166	-0.0122	-2.658E-21	-1.956E-21
**L11**	End-to-End	-0.0447	0.0036	0.0021	-0.0298	0.0031	0.0018	-0.1901	-0.1276	-3.045E-20	-2.044E-20
	Side-to-Side	0.0039	0.0274	0.0222	0.0033	0.0249	0.0255	-0.0150	-0.0238	-2.409E-21	-3.811E-21
**L15**	End-to-End	-0.0350	-0.0015	0.0087	-0.0350	-0.0014	0.0095	-0.0907	-0.0892	-1.453E-20	-1.429E-20
	Side-to-Side	0.0170	0.0111	0.0001	0.0162	0.0093	-0.0022	-0.0388	-0.0262	-6.219E-21	-4.194E-21
**L18**	End-to-End	-0.0183	0.0013	-0.0268	-0.0178	0.0007	-0.0269	-0.1124	-0.1134	-1.801E-20	-1.817E-20
	Side-to-Side	0.0156	0.0351	0.0016	0.0158	0.0349	-0.0001	-0.0603	-0.0532	-9.662E-21	-8.519E-21
**L20**	End-to-End	-0.0251	-0.0244	0.0049	-0.0252	-0.0251	0.0040	-0.1269	-0.1301	-2.033E-20	-2.084E-20
	Side-to-Side	0.0034	0.0067	0.0050	0.0029	0.0068	0.0049	-0.0240	-0.0223	-3.845E-21	-3.566E-21
**L22**	End-to-End	-0.0159	-0.0247	-0.0209	-0.0155	-0.0239	-0.0209	-0.1441	-0.1411	-2.309E-20	-2.260E-20
**S6**	End-to-End	-0.0047	0.0155	-0.0012	-0.0020	0.0133	-0.0013	-0.0636	-0.0492	-1.018E-20	-7.879E-21
**S8**	End-to-End	-0.0187	-0.0233	0.0022	-0.0183	-0.0226	0.0023	-0.1230	-0.1199	-1.970E-20	-1.921E-20
	Side-to-Side	0.0179	-0.0066	-0.0373	0.0193	-0.0060	-0.0370	-0.1191	-0.1153	-1.909E-20	-1.847E-20
**S11**	End-to-End	0.0270	-0.0130	-0.0012	0.0241	-0.0108	-0.0011	-0.1305	-0.1149	-2.092E-20	-1.841E-20
	Side-to-Side	0.0211	0.0013	0.0190	0.0263	0.0019	0.0186	0.0256	0.0327	4.096E-21	5.245E-21
**L10+L11**	End-to-End	-0.0268	-0.0075	-0.0210	-0.0247	-0.0086	-0.0179	-0.1424	-0.1262	-2.282E-20	-2.022E-20
	Side-to-Side	0.0075	0.0082	0.0083	0.0064	0.0070	0.0087	-0.0498	-0.0428	-7.973E-21	-6.860E-21
**L15+L18**	End-to-End	-0.0123	0.0015	-0.0355	-0.0122	0.0021	-0.0355	-0.1081	-0.1061	-1.732E-20	-1.700E-20
	Side-to-Side	-0.0102	0.0025	0.0252	-0.0111	0.0031	0.0255	-0.1056	-0.1100	-1.692E-20	-1.763E-20
**L9+S6**	End-to-End	0.0079	0.0072	0.0012	0.0058	0.0058	0.0015	-0.0460	-0.0361	-7.375E-21	-5.782E-21
	Side-to-Side	0.0171	0.1033	0.0096	0.0187	0.1017	0.0121	-0.2165	-0.2020	-3.469E-20	-3.236E-20
**S6+S11**	End-to-End	0.0003	0.0170	-0.0156	0.0000	0.0173	-0.0154	-0.0833	-0.0843	-1.335E-20	-1.351E-20
	Side-to-Side	0.1088	-0.0029	0.0818	0.1095	-0.0012	0.0793	0.1834	0.1777	2.938E-20	2.847E-20
**L9+S11**	End-to-End	-0.0353	0.0144	-0.0146	-0.0367	0.0131	-0.0142	-0.1056	-0.1129	-1.692E-20	-1.808E-20
	Side-to-Side	0.0670	-0.0045	0.0045	0.0668	-0.0069	0.0018	0.1319	0.1453	2.113E-20	2.328E-20
**L9+S6+S11**	End-to-End	-0.0369	0.0155	-0.0108	-0.0355	0.0144	-0.0111	-0.1066	-0.1048	-1.708E-20	-1.679E-20
	Side-to-Side	0.0948	-0.0039	0.0162	0.0938	-0.0039	0.0156	0.2101	0.2088	3.366E-20	3.345E-20
	Side-to-Side2	0.0803	0.0348	0.0649	0.0788	0.0285	0.0667	0.1007	0.1243	1.613E-20	1.992E-20

Continued page of Table S12

RPs	Possible Binding Modes	GAP2									
		$E_x{}^R$ (V/Å)	$E_y{}^R$ (V/Å)	$E_z{}^R$ (V/Å)	$E_x{}^T$ (V/Å)	$E_y{}^T$ (V/Å)	$E_z{}^T$ (V/Å)	U^{Ribo}(*e*V) [a]	U^{TPI} (*e*V) [b]	U^{Ribo} (J) [a]	U^{TPI} (J) [b]
**L9**	End-to-End	0.0237	0.0158	0.0322	0.0240	0.0104	0.0322	0.0625	0.0796	1.001E-20	1.276E-20
	Side-to-Side	0.0035	0.0011	0.0036	0.0033	0.0010	0.0039	0.0005	0.0017	8.280E-23	2.800E-22
**L10**	End-to-End	0.0092	0.0056	-0.0056	0.0085	0.0067	-0.0054	0.0151	0.0125	2.417E-21	2.009E-21
	Side-to-Side	-0.0148	-0.0111	-0.0193	-0.0147	-0.0116	-0.0191	-0.0981	-0.0994	-1.572E-20	-1.593E-20
**L11**	End-to-End	0.0019	0.0103	-0.0031	0.0026	0.0096	-0.0028	-0.0028	-0.0058	-4.428E-22	-9.331E-22
	Side-to-Side	0.0183	0.0081	-0.0060	0.0187	0.0081	-0.0064	-0.0773	-0.0798	-1.239E-20	-1.278E-20
**L15**	End-to-End	-0.0063	-0.0037	0.0123	-0.0056	-0.0032	0.0122	0.0295	0.0259	4.721E-21	4.153E-21
	Side-to-Side	-0.0199	0.0054	-0.0179	-0.0189	0.0046	-0.0169	-0.0868	-0.0824	-1.391E-20	-1.319E-20
**L18**	End-to-End	-0.0128	-0.0033	-0.0104	-0.0133	-0.0038	-0.0100	0.0650	0.0664	1.041E-20	1.064E-20
	Side-to-Side	-0.0124	-0.0073	-0.0281	-0.0113	-0.0060	-0.0272	-0.1263	-0.1213	-2.024E-20	-1.944E-20
**L20**	End-to-End	0.0027	-0.0036	0.0058	0.0031	-0.0026	0.0057	0.0203	0.0158	3.245E-21	2.530E-21
	Side-to-Side	-0.0233	0.0211	-0.0159	-0.0230	0.0211	-0.0158	-0.0744	-0.0735	-1.192E-20	-1.178E-20
**L22**	End-to-End	0.0006	-0.0171	-0.0010	0.0004	-0.0178	-0.0019	0.0689	0.0725	1.104E-20	1.162E-20
**S6**	End-to-End	0.0002	-0.0311	0.0018	0.0000	-0.0309	0.0019	-0.1182	-0.1172	-1.893E-20	-1.878E-20
**S8**	End-to-End	-0.0098	-0.0023	0.0158	-0.0089	-0.0014	0.0190	0.0390	0.0333	6.247E-21	5.333E-21
	Side-to-Side	0.0034	0.0046	0.0006	0.0038	0.0050	0.0003	-0.0039	-0.0029	-6.222E-22	-4.724E-22
**S11**	End-to-End	-0.0116	0.0126	0.0118	-0.0134	0.0140	0.0124	-0.0783	-0.0879	-1.254E-20	-1.408E-20
	Side-to-Side	0.0072	-0.0238	-0.0124	0.0064	-0.0220	-0.0120	-0.1134	-0.1053	-1.817E-20	-1.687E-20
**L10+L11**	End-to-End	-0.0055	0.0011	-0.0066	-0.0051	0.0008	-0.0061	0.0351	0.0327	5.625E-21	5.238E-21
	Side-to-Side	-0.0326	-0.0014	0.0087	-0.0324	-0.0010	0.0084	-0.1021	-0.1010	-1.636E-20	-1.617E-20
**L15+L18**	End-to-End	-0.0038	0.0084	-0.0028	-0.0038	0.0085	-0.0026	0.0039	0.0032	6.272E-22	5.119E-22
	Side-to-Side	0.0093	0.0043	-0.0090	0.0087	0.0044	-0.0093	-0.0547	-0.0532	-8.762E-21	-8.531E-21
**L9+S6**	End-to-End	-0.0275	-0.0007	0.0144	-0.0272	-0.0006	0.0141	-0.0704	-0.0698	-1.128E-20	-1.119E-20
	Side-to-Side	0.0157	-0.0117	0.0177	0.0146	-0.0106	0.0170	-0.1022	-0.0951	-1.637E-20	-1.524E-20
**S6+S11**	End-to-End	0.0031	-0.0249	0.0103	0.0030	-0.0247	0.0103	-0.1096	-0.1087	-1.756E-20	-1.742E-20
	Side-to-Side	0.0068	-0.0229	-0.0077	0.0060	-0.0217	-0.0073	-0.1039	-0.0977	-1.665E-20	-1.566E-20
**L9+S11**	End-to-End	0.0019	0.0127	0.0078	0.0013	0.0126	0.0078	-0.0306	-0.0282	-4.907E-21	-4.525E-21
	Side-to-Side	0.0055	-0.0172	-0.0244	0.0058	-0.0169	-0.0246	-0.1226	-0.1231	-1.964E-20	-1.972E-20
**L9+S6+S11**	End-to-End	-0.0048	-0.0008	0.0044	-0.0050	-0.0007	0.0046	0.0209	0.0217	3.353E-21	3.485E-21
	Side-to-Side	0.0073	-0.0228	-0.0147	0.0065	-0.0215	-0.0141	-0.1153	-0.1086	-1.847E-20	-1.740E-20
	Side-to-Side2	0.0007	-0.0219	0.0223	0.0030	-0.0125	0.0165	-0.1267	-0.0819	-2.031E-20	-1.311E-20

[a] Interaction energies of ribosome-bound TPI at the center of mass of substrates in *e*V or J.
[b] Interaction energies of free TPI at the center of mass of substrates in *e*V or J.

Table S13. Differences in dipole-electric field interaction energies between free and ribosome-bound TPI for each site.

RPs	Possible Binding Modes	DHAP			GAP		
		$U_1^{Ribo+TPI} - U_1^{TPI}$ (kcal/mol) [a]	$U_2^{Ribo+TPI} - U_2^{TPI}$ (kcal/mol) [b]	Average of two active sites (kcal/mol) [c]	$U_1^{Ribo+TPI} - U_1^{TPI}$ (kcal/mol) [d]	$U_2^{Ribo+TPI} - U_2^{TPI}$ (kcal/mol) [e]	Average of two active sites (kcal/mol) [f]
L9	End-to-End	0.0044	0.0405	0.0224	-0.0961	-0.3958	-0.2460
	Side-to-Side	-0.0692	-0.0486	-0.0589	0.0048	-0.0284	-0.0118
L10	End-to-End	-0.2131	-0.0298	-0.1214	-0.2449	0.0587	-0.0931
	Side-to-Side	0.0439	0.0541	0.0490	-0.1010	0.0298	-0.0356
L11	End-to-End	-1.7237	-0.1540	-0.9389	-1.4404	0.0705	-0.6849
	Side-to-Side	0.0036	0.0094	0.0065	0.2016	0.0562	0.1289
L15	End-to-End	-0.0398	0.0029	-0.0184	-0.0336	0.0816	0.0240
	Side-to-Side	-0.0156	-0.1627	-0.0892	-0.2913	-0.1029	-0.1971
L18	End-to-End	-0.0526	0.0802	0.0138	0.0234	-0.0327	-0.0047
	Side-to-Side	-0.1475	-0.1902	-0.1689	-0.1645	-0.1154	-0.1400
L20	End-to-End	-0.0610	0.0313	-0.0149	0.0740	0.1028	0.0884
	Side-to-Side	-0.0126	-0.0093	-0.0110	-0.0401	-0.0201	-0.0301
L22	End-to-End	0.0009	-0.0910	-0.0450	-0.0696	-0.0831	-0.0763
S6	End-to-End	-0.2061	-0.0309	-0.1185	-0.3315	-0.0222	-0.1768
S8	End-to-End	-0.0898	-0.0206	-0.0552	-0.0707	0.1315	0.0304
	Side-to-Side	-0.0586	-0.1057	-0.0821	-0.0891	-0.0215	-0.0553
S11	End-to-End	-0.8046	0.1296	-0.3375	-0.3599	0.2210	-0.0695
	Side-to-Side	0.0953	-0.0876	0.0038	-0.1653	-0.1878	-0.1766
L10+L11	End-to-End	-0.3066	0.1197	-0.0934	-0.3738	0.0558	-0.1590
	Side-to-Side	-0.1429	-0.0232	-0.0831	-0.1600	-0.0265	-0.0933
L15+L18	End-to-End	-0.0459	-0.1013	-0.0736	-0.0466	0.0166	-0.0150
	Side-to-Side	0.0515	-0.0639	-0.0062	0.1016	-0.0331	0.0342
L9+S6	End-to-End	-0.1986	0.0684	-0.0651	-0.2291	-0.0139	-0.1215
	Side-to-Side	-0.9846	-0.3069	-0.6458	-0.3351	-0.1632	-0.2491
S6+S11	End-to-End	0.0377	-0.1103	-0.0363	0.0227	-0.0197	0.0015
	Side-to-Side	-0.0725	-0.0066	-0.0395	0.1313	-0.1431	-0.0059
L9+S11	End-to-End	0.1248	-0.0468	0.0390	0.1670	-0.0550	0.0560
	Side-to-Side	-0.1466	-0.0298	-0.0882	-0.3083	0.0111	-0.1486
L9+S6+S11	End-to-End	-0.1033	-0.0923	-0.0978	-0.0417	-0.0190	-0.0303
	Side-to-Side	-0.0777	-0.0932	-0.0855	0.0307	-0.1534	-0.0613
	Side-to-Side2	-0.0051	-0.9995	-0.5023	-0.5441	-1.0347	-0.7894

[a] Interaction energy difference between free and ribosome-bound TPI for DHAP1.
[b] Interaction energy difference between free and ribosome-bound TPI for DHAP2.
[c] Average of interaction energy difference between the two active sites for DHAP.
[d] Interaction energy difference between free and ribosome-bound TPI for GAP1.
[e] Interaction energy difference between free and ribosome-bound TPI for GAP2.
[f] Average of interaction energy difference between the two active sites for GAP.

Table S14. Ribosome-binding Glycolytic Enzymes (related to Figure 8).

<table>
<thead>
<tr><th>Glycolytic Enzymes</th><th>Gene Name</th><th>Uniport Entry</th><th>Enzyme Source</th><th>Involved Ribosome</th><th>Reference</th></tr>
</thead>
<tbody>
<tr><td>Pyruvate kinase PKM</td><td>Pkm</td><td>P52480</td><td rowspan="7">Mus musculus (Mouse)</td><td rowspan="7">80S Eukaryotic Ribosome</td><td rowspan="7">[1]</td></tr>
<tr><td>α-Enolase</td><td>Eno1</td><td>P17182</td></tr>
<tr><td>Triosephosphate isomerase</td><td>Tpi1</td><td>P17751</td></tr>
<tr><td>Glucose 6-phosphate isomerase</td><td>Gpi</td><td>P06745</td></tr>
<tr><td>Phosphoglycerate mutase 1</td><td>Pgam1</td><td>Q9DBJ1</td></tr>
<tr><td>Fructose-bisphosphate aldolase A</td><td>Aldoa</td><td>P05064</td></tr>
<tr><td>Lactate dehydrogenase A</td><td>Ldha</td><td>P06151</td></tr>
<tr><td>ATP-dependent 6-phosphofructokinase</td><td>pfkA</td><td>O34529</td><td rowspan="4">Bacillus subtilis</td><td rowspan="8">70S Prokaryotic Ribosome</td><td rowspan="4">[2]</td></tr>
<tr><td>Glyceraldehyde-3-phosphate dehydrogenase 1</td><td>gapA</td><td>P09124</td></tr>
<tr><td>Phosphoglycerate kinase</td><td>pgk</td><td>P40924</td></tr>
<tr><td>Enolase</td><td>eno</td><td>P37869</td></tr>
<tr><td>Phosphoglycerate kinase</td><td>pgk</td><td>P0A799</td><td rowspan="2">Escherichia coli (strain K12)</td><td rowspan="2">[3]</td></tr>
<tr><td>Pyruvate kinase I</td><td>pykF</td><td>P0AD61</td></tr>
<tr><td>Pyruvate kinase</td><td>pyk</td><td>Q02499</td><td>Bacillus stearothermophilus</td><td>[4]</td></tr>
<tr><td>Glyceraldehyde-3-phosphate dehydrogenase</td><td>GAPD</td><td>P46406</td><td>Oryctolagus cuniculus (Rabbit)</td><td>[5]</td></tr>
</tbody>
</table>

[1] Simsek, D.; Tiu, G. C.; Flynn, R. A.; Byeon, G. W.; Leppek, K.; Xu, A. F.; Chang, H. Y.; Barna, M., The Mammalian Ribo-interactome Reveals Ribosome Functional Diversity and Heterogeneity. *Cell* **2017,** *169* (6), 1051-1065.

[2] Commichau, F. M.; Rothe, F. M.; Herzberg, C.; Wagner, E.; Hellwig, D.; Lehnik-Habrink, M.; Hammer, E.; Volker, U.; Stulke, J., Novel activities of glycolytic enzymes in Bacillus subtilis: interactions with essential proteins involved in mRNA processing. *Mol Cell Proteomics* **2009,** *8* (6), 1350-1360.

[3] Chowdhury, S.; Hepper, S.; Lodi, M. K.; Saier, M. H., Jr.; Uetz, P., The Protein Interactome of Glycolysis in Escherichia coli. *Proteomes* **2021,** *9* (2), 16

[4] Yu, J.; Ramirez, L. M.; Premo, A.; Busch, D. B.; Lin, Q.; Burz, D. S.; Shekhtman, A., Ribosome-Amplified Metabolism, RAMBO, Measured by NMR Spectroscopy. *Biochemistry* **2021,** *60* (24), 1885-1895.

[5] Ryazanov, A. G., Glyceraldehyde-3-phosphate dehydrogenase is one of the three major RNA-binding proteins of rabbit reticulocytes. *FEBS Lett* **1985,** *192* (1), 131-134.
